# FeNb_2_O_6_ as a High‐Performance Anode for Sodium‐Ion Batteries Enabled by Structural Amorphization Coupled with NbO_6_ Local Ordering

**DOI:** 10.1002/adma.202504100

**Published:** 2025-07-29

**Authors:** Yanchen Liu, Ana Guilherme Buzanich, Paola Alippi, Luciano A. Montoro, Kug‐Seung Lee, Taeyeol Jeon, Kilian Weißer, Martin A. Karlsen, Patrícia A. Russo, Nicola Pinna

**Affiliations:** ^1^ Department of Chemistry and The Center for the Science of Materials Berlin Humboldt‐Universität zu Berlin Brook‐Taylor‐Str. 2 12489 Berlin Germany; ^2^ Bundesanstalt für Materialforschung und ‐prüfung (BAM) Richard‐Willstätter‐Straße 11 12489 Berlin Germany; ^3^ CNR‐ISM Consiglio Nazionale delle Ricerche Istituto di Struttura della Materia Via Salaria Km 29.3 Monterotondo Stazione Roma I‐00015 Italy; ^4^ Department of Chemistry Universidade Federal de Minas Gerais Belo Horizonte MG 31270‐901 Brazil; ^5^ PLS‐II Beamline Depart. / Pohang Accelerator Laboratory POSTECH 80 Jigokro‐127‐beongil Nam‐gu Pohang Gyeongbuk 37673 South Korea; ^6^ Deutsches Elektronen‐Synchrotron DESY Notkestr. 85 22607 Hamburg Germany

**Keywords:** high‐rate capability, intercalation‐type anodes, iron niobate, operando studies, sodium‐ion batteries

## Abstract

Pseudocapacitance‐type transition metal oxides have been extensively investigated as anodes for lithium‐ion batteries (LIBs). Currently, they are also gaining attention for sodium‐ion batteries (SIBs) due to their low volume change and safety. However, their performance in sodium storage remains limited, primarily due to the larger Na^+^ ion radius. Here, for the first time, an iron niobate is reported with a columbite structure as a high‐performance sodium storage anode. The presence of iron triggers the loss of long‐range order through disorder of the FeO_6_ octahedra local structure, subsequently allowing reversible sodium storage in an amorphous phase. Simultaneously, the formation of short‐range ordered zigzag‐chain structures within the NbO_6_ planes creates a “skeleton” that offers abundant active sites for pseudocapacitive ion storage and enhanced ion diffusion pathways. These characteristics of FeNb_2_O_6_ make it an effective intercalation host, offering high capacity along with fast Na^+^ kinetics, as demonstrated through operando and ex situ characterizations. It leads to an applicable reversible capacity (>300 mAh g^−1^) with a favorable average voltage of ≈0.6 V and excellent rate capability (180.4 mAh g^−1^ at a current density of 2 A g^−1^). This study provides insights into the development of intrinsically active transition metal oxides for Na^+^‐ion intercalation.

## Graphical abstract

FeNb_2_O_6_ is applied for the first time as a high‐performance anode material for sodium‐ion batteries. The presence of iron disrupts the long‐range order by local disorder of the FeO_6_ octahedra during initial sodiation. Simultaneously, the formation of short‐range ordered zigzag‐chain structures within the NbO_6_ planes establishes a robust intercalation host that maintains abundant active sites for pseudocapacitive intercalation and enhanced ion‐diffusion pathways.

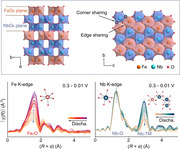

## 1 Introduction

Sodium‐ion batteries are considered promising for large‐scale electric energy storage due to the high abundance and widespread distribution of sodium.^[^ ^1^ ^]^ However, their practical application is hindered by their low energy density and unsatisfactory long‐cycling life, primarily due to the large radius and mass of the Na^+^ ion.^[^ ^2^ ^]^ Battery performance largely depends on the properties of the cathode and anode materials. Recently, cathode materials, such as transition‐metal oxides, Prussian blue analogs, and NASICON‐type phosphates, have been extensively studied as reversible host materials for Na^+^ ions, leading to substantial progress in the field.^[^ ^3^ ^]^ In contrast, developing anodes for high‐performance SIBs is still challenging. Hard carbon, a common carbon‐based material used in SIBs, offers an excellent overall storage performance in terms of capacity and cycling lifespan.^[^ ^4^ ^]^ However, its low operating voltage (close to 0 V versus Na^+^/Na) hardly satisfies the safety and high power density requirements for practical application, due to the risk of Na‐dendrite formation. One of the most urgent tasks for facilitating the practical application of SIBs is therefore to explore suitable electrode materials that exhibit long‐term cycling stability, high capacity, and rate capability. Additionally, maintaining a moderate average sodiation voltage ranging from 0.3 to 1.0 V is crucial for balancing Na‐dendrite formation and minimizing the voltage penalty of SIBs.^[^ ^5^ ^]^

Transition‐metal oxides, such as titanium‐ and niobium‐based materials, have been investigated as anode materials for constructing safe, durable lithium‐ion batteries with high power density.^[^ ^6^ ^]^ However, when used in sodium‐ion batteries, these materials do not fully reach their potential performance and can even become inactive or rapidly inactive. This is due to the larger radius of the Na^+^ ions compared to the Li^+^ ions, which affects the intercalation kinetics and restricts the degree of sodiation,^[^ ^7^ ^]^ thus limiting the utilization of the inherent electrochemical properties of these materials in SIBs. For example, anatase TiO_2_ has a favorable working voltage of 0.8 V during Na^+^‐ion insertion and extraction, but its performance strongly depends on particle size.^[^ ^8^ ^]^ Only when the particle size is reduced to ≈10 nm does the material become almost completely amorphous during sodiation, and is therefore able to function as a reversible host for Na^+^‐ion intercalation and deliver optimal electrochemical performance. In contrast, larger particles remain predominantly crystalline, which limits their performance. Similarly, it was found that Li_4_Ti_5_O_12_ only exhibits acceptable electrochemical activity as an anode in SIBs when its particle size is ≈44 nm, delivering a higher capacity than Li_4_Ti_5_O_12_ with particle sizes of ≈120 or 440 nm.^[^ ^9^ ^]^ Nb‐based oxides have not been widely investigated as anode materials for SIBs.^[^ ^10^ ^]^ Kim et al. were the first to report a mesoporous Nb_2_O_5_/carbon composite as a sodium insertion material.^[^ ^11^ ^]^ This composite delivers a reversible capacity of 175 mAh g^−1^ at a moderate operating voltage of ≈0.7 V and maintains a capacity of ≈60 mAh g^−1^ at 1 A g^−1^. Micron‐sized TiNb_2_O_7_ is electrochemically inactive for Na^+^‐ion intercalation. However, its initial capacity can be increased to 175 mAh g^−1^ through a ball‐milling process that decreases the size of the particles to the nanoscale.^[^ ^12^ ^]^ Despite the improvement, this material still shows limited rate capability, with the capacity decreasing to 60 mAh g^−1^ when cycled at 0.5 A g^−1^. Although employing nanostructures and porous materials can shorten Na^+^‐ion diffusion paths and improve material utilization,^[^ ^13^ ^]^ these approaches tend to raise costs, result in low tap density, and promote side reactions during the electrochemical process. Therefore, exploring and developing anode materials that are intrinsically active for Na^+^‐ion intercalation, along with gaining a thorough understanding of their sodium storage mechanisms, is essential for advancing the development of SIBs anodes.

Ternary niobates with columbite structure, *M*Nb_2_O_6_ (*M* = Mn, Co, Ni, Fe, Cu, Zn, Cd, Ca, Mg, etc.), have attracted attention as anode materials for lithium‐ion batteries due to their unique crystalline structure that contains 1D channels, which facilitate rapid Li^+^ insertion and extraction.^[^ ^14^ ^]^ Previous studies showed that the Li‐ion storage performance is affected by the *M* species incorporated in *M*Nb_2_O_6_. Xia et al. demonstrated that NiNb_2_O_6_ served as an intrinsic high‐rate material and that the oxidation of all the transition metal ions (Ni, Nb) enables the formation of up to Li_3_NiNb_2_O_6_ after full lithiation.^[^ ^14c^ ^]^ Moreover, the expansion of the NiO_6_ octahedra in NiNb_2_O_6_ almost fully offsets the shrinkage of the NbO_6_ octahedra, ensuring a “zero strain” behavior for excellent stability.^[^ ^14d^ ^]^ A series of single‐crystal *M*Nb_2_O_6_ (*M* = Cr, Co, Zn, Mn, Mg, Ca) has been developed, showing *M*‐dependent electron/ion transport capabilities.^[^ ^14e^ ^]^ CdNb_2_O_6_ was found to exhibit the highest electronic and Li^+^ diffusion rates (102.8 mAh g^−1^ at 10 A g^−1^), with the lithiation process involving a solid‐solution charge storage mechanism (volumetric change < 0.59%). Carbon‐coated FeNb_2_O_6_ exhibited a reversible capacity of 139 mAh g^−1^ along with an average capacity of 125.5 mAh g^−1^ over the first 50 cycles as an insertion‐type electrode material for LIBs.^[^ ^14g^ ^]^ Even though the performance of FeNb_2_O_6_ in LIBs is not particularly remarkable, its intrinsic structural and electrochemical characteristics, as well as the earth‐abundance and non‐toxicity of iron, still make it a promising candidate for energy storage applications. Herein, we propose for the first time an iron niobate with columbite structure as a high‐performance sodium storage anode. A combination of characterization techniques, including operando X‐ray diffraction (XRD), ex situ pair distribution function (PDF), operando X‐ray absorption spectroscopy (XAS), and Density Functional Theory (DFT), was employed to probe the Na^+^ storage mechanism in terms of the long‐range and local structures during sodiation and de‐sodiation. The results indicate that iron plays a key role in facilitating the loss of long‐range order through disorder of the FeO_6_ octahedra local structure, and, consequently, on the performance of the material. Simultaneously, the formation of short‐range ordered zigzag‐chain structures within the NbO_6_ planes creates a “skeleton” that allows the material to maintain abundant active sites for pseudocapacitive intercalation and enhanced ion diffusion pathways. The amorphization process during sodiation of the submicron‐sized iron niobate generates an effective intercalation host for fast Na^+^ insertion and extraction, along with high capacity. The properties of iron niobate, which lead to its high capacity, good rate capability, and attractive working voltage, show significant potential for SIB applications.

## 2 Results

### 2.1 Characterization

**Figure 1:**
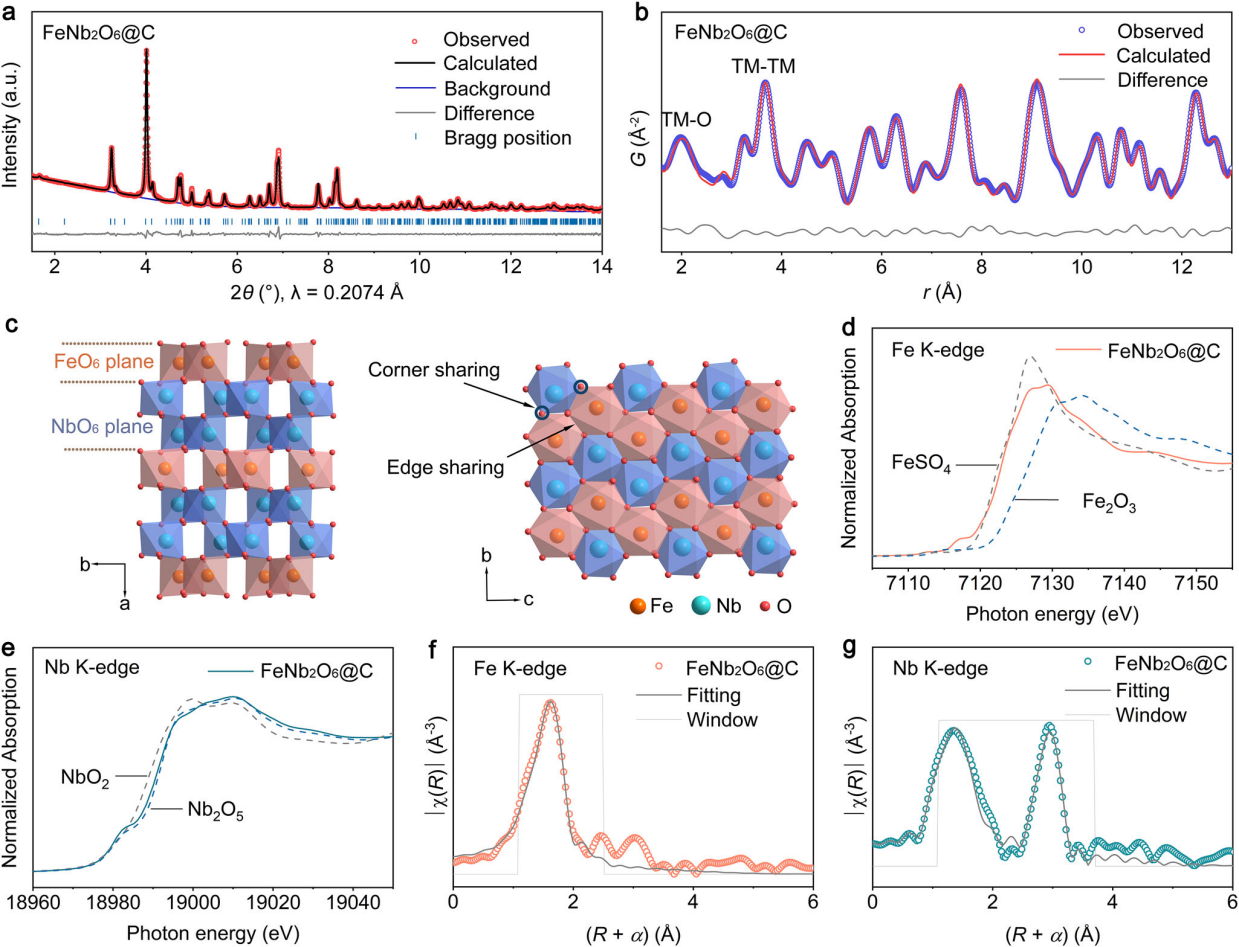
a) XRD pattern of FeNb_2_O_6_@C and corresponding Rietveld refinement. b) PDF pattern of FeNb_2_O_6_@C and corresponding fitting. c) Schematic illustration of the structure of FeNb_2_O_6_. d) Fe K‐edge and e) Nb K‐edge XANES spectra of FeNb_2_O_6_@C. f) Fe K‐edge FT‐EXAFS spectrum of FeNb_2_O_6_@C and fit of the first coordination shell. g) Nb K‐edge FT‐EXAFS spectrum of FeNb_2_O_6_@C and fit of the first and second coordination shells.

FeNb_2_O_6_@C was synthesized using a hydrothermal method, followed by annealing in an argon atmosphere to form the crystalline phase. Subsequently, the metal oxide was uniformly coated with a polydopamine shell via polymerization at room temperature and then calcined under argon to yield FeNb_2_O_6_ coated with an amorphous carbon layer. Similarly, niobium pentoxide coated with a carbon shell (*T*‐Nb_2_O_5_@C) was synthesized using the same procedure and employed for comparison and to help better understand the sodium storage behavior of the iron niobate. The X‐ray diffraction patterns of FeNb_2_O_6_@C and *T*‐Nb_2_O_5_@C and the results of the Rietveld refinement of the diffractograms are shown in **Figure** **1**a, Figures S1 and S2, Tables S1, and S2 (Supporting Information). FeNb_2_O_6_@C has an orthorhombic columbite structure (*Pbcn*) with refined lattice parameters *a* = 14.2459 Å, *b* = 5.7357 Å, *c* = 5.0464 Å, and *α* = *β* = *γ* = 90°, and a unit cell volume of 412.3 Å^3^ (Figure 1a; Table S1, Supporting Information). All the reflections in the diffractogram of *T*‐Nb_2_O_5_@C are indexed to the orthorhombic phase (*Pbam*) of niobium pentoxide (Figure S1a, Supporting Information). As illustrated in Figure 1c, the Fe and Nb atoms in FeNb_2_O_6_ are sixfold coordinated and occupy octahedral sites. The NbO_6_ octahedra connect to adjacent FeO_6_ and NbO_6_ octahedra along the *a* axis via corner‐sharing, and the cations within the octahedra alternate according to the sequence Fe‐Nb‐Nb‐Fe‐Nb‐Nb‐Fe. Along the *c* axis, adjacent NbO_6_‐NbO_6_ and adjacent FeO_6_‐FeO_6_ share edges and form independent zigzag Fe and Nb octahedra chains within the *bc* plane. *T*‐Nb_2_O_5_ contains Nb atoms in both octahedral and pentagonal bipyramidal coordination environments (Figure S3, Supporting Information). Synchrotron X‐ray pair distribution function analyses were conducted to probe the atomic pair distribution in the structures of the materials. The PDF peak positions directly show the interatomic distances between pairs of atoms in the structure. The PDF of FeNb_2_O_6_@C is accurately fitted using the orthorhombic *Pbcn* model (Figure 1b), and the resulting parameter values given in Table S3 (Supporting Information) are consistent with the XRD refinement results. The first peak in the PDF corresponds to the interatomic distances between metal and oxygen atoms in the first coordination shell, while the subsequent peaks represent metal–metal distances within the structure. The maximum of the first peak is located at a lower *r* value in the PDF of FeNb_2_O_6_@C than in that of *T*‐Nb_2_O_5_@C (Figure S4, Supporting Information). This shift can be attributed to the contribution of Fe–O distances to the FeNb_2_O_6_@C PDF, as well as to the presence of sevenfold coordinated Nb atoms (in addition to sixfold coordinated ones) in *T*‐Nb_2_O_5_, in contrast with the exclusively sixfold coordinated Fe and Nb atoms in FeNb_2_O_6_.

The oxidation state and local structure of the metal cations were investigated using X‐ray absorption spectroscopy. The edge energy in the Fe K‐edge X‐ray absorption near‐edge structure (XANES) spectra of FeNb_2_O_6_@C indicates that the oxidation state of the Fe cations is 2+, the same as in FeSO_4_ (Figure 1d). The edge position in the Nb K‐edge XANES spectra (Figure 1e and Figure S5, Supporting Information) reveals that niobium is in the 5+ oxidation state in both FeNb_2_O_6_@C and *T*‐Nb_2_O_5_@C. The pre‐edge features observed in both the Fe and Nb XANES spectra are due to electron transitions from the 1s orbitals to the p component in d–p mixed orbitals, and reflect some degree of distortion of the FeO_6_ and NbO_6_ octahedra.^[^ ^15^ ^]^ Additional information on the local structure around the metal cations was obtained from the Fe and Nb K‐edge extended X‐ray absorption fine structure (EXAFS) spectra. The Fe and Nb K‐edge Fourier transform (FT) EXAFS spectra of FeNb_2_O_6_@C are shown in Figure 1f,g, respectively, and the corresponding fitting results are listed in Tables S4 and S5 (Supporting Information). The results confirm the six‐coordination of the Fe and Nb on the first Fe‒O and Nb‒O shell in FeNb_2_O_6_@C. The first peaks of the Fe and Nb EXAFS spectra are fitted with two and four scattering paths, respectively, suggesting that Fe and Nb form distorted octahedra, which is consistent with the pre‐edge features in the Fe and Nb K‐edge XANES spectra. The PDF and XAS data for FeNb_2_O_6_@C will be discussed in greater detail in a subsequent section.

Transmission electron microscopy (TEM) imaging shows that FeNb_2_O_6_@C and *T*‐Nb_2_O_5_@C (Figures S6 and S7, Supporting Information) consist of similarly sized particles, ranging from 100 to 200 nm, uniformly coated with an amorphous carbon layer. The presence of the amorphous carbon is also detected in the Raman spectra of FeNb_2_O_6_@C and *T*‐Nb_2_O_5_@C, which display the characteristic D and G bands located at 1348 and 1595 cm^−1^, respectively (Figure S8, Supporting Information). The high‐resolution TEM (HR‐TEM) image of FeNb_2_O_6_@C in Figure S7 (Supporting Information) confirms the high crystallinity of the material, showing lattice fringes with a spacing of 0.37 nm, which is consistent with the *d*‐spacing of the (310) planes of FeNb_2_O_6_. Moreover, the (200), (002), and (202) planes in the selected area electron diffraction (SAED) pattern of FeNb_2_O_6_@C match the diffraction pattern of the orthorhombic (*Pbcn*) structure. For *T*‐Nb_2_O_5_@C, a crystal lattice spacing of 0.30 nm is observed, consistent with the interplanar spacing of the (200) planes of orthorhombic *T*‐Nb_2_O_5_. The energy dispersive X‐ray spectroscopy (EDS) elemental maps of FeNb_2_O_6_@C and uncoated FeNb_2_O_6_ indicate a homogeneous distribution of the elements in the materials (Figures S7 and S9, Supporting Information). It is noted that the particles of uncoated FeNb_2_O_6_ and FeNb_2_O_6_@C exhibit internal voids resulting from the synthesis process due to the Kirkendall effect.^[^ ^16^ ^]^ The formation of Kirkendall voids is caused by the difference in diffusion fluxes of atoms across an interface in a diffusion couple under elevated temperature conditions.

### 2.2 Na‐Ion Electrochemical Behavior

**Figure 2:**
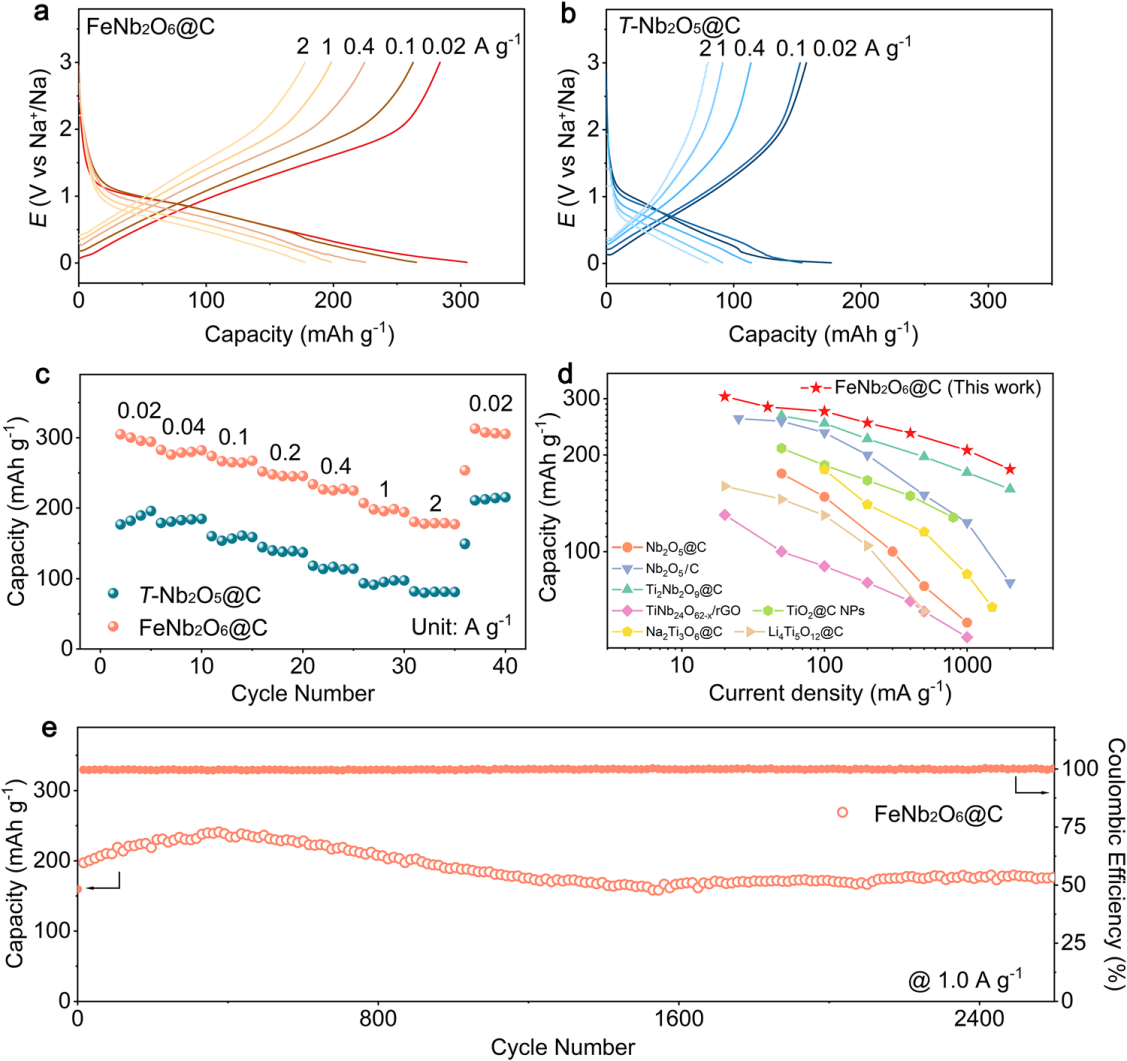
Discharge–charge curves of a) FeNb_2_O_6_@C and b) *T*‐Nb_2_O_5_@C at different rates. c) Rate capability of FeNb_2_O_6_@C and *T*‐Nb_2_O_5_@C. d) Comparison of the rate capability of FeNb_2_O_6_@C with that of reported Ti‐based and Nb‐based oxide composites with carbon.^[^ ^11^, ^19^ ^]^ e) Cycling performance of FeNb_2_O_6_@C at 1.0 A g^−1^.

The electrochemical performance of FeNb_2_O_6_@C and *T*‐Nb_2_O_5_@C was investigated in half‐cells with Na foil anodes. The rate performance was studied by galvanostatic discharge and charge measurements at various current densities ranging from 0.02 to 2 A g^−1^, in the voltage window 0.01–3.0 V (vs Na^+^/Na) as shown in **Figures** **2**a–c and S10 (Supporting Information). FeNb_2_O_6_@C exhibits a high initial capacity of 604.5 mAh g^−1^ and reversible capacity of 304.9 mAh g^−1^ at a low current density of 0.02 A g^−1^, while a smaller initial capacity of 408.1 mAh g^−1^ and reversible capacity of 180.5 mAh g^−1^ are obtained for *T*‐Nb_2_O_5_@C. Both materials show linear voltage profiles without obvious plateau regions after the first cycle, which are consistent with surface‐redox and intercalation pseudocapacitive mechanisms.^[^ ^6^, ^8^ ^]^ The theoretical capacities of FeNb_2_O_6_ and Nb_2_O_5_, calculated based on a one‐electron transfer per transition metal, are 238.2 and 201.7 mAh g^−1^, respectively. The higher reversible capacity observed for FeNb_2_O_6_@C with respect to its theoretical value can be attributed to surface‐redox pseudocapacitive behavior and the occurrence of multi‐electron transfer processes involving Nb^5+^, which reaches oxidation states below 4+ during the lithiation process. In contrast, Nb_2_O_5_@C exhibits a reversible capacity close to its theoretical value. During the first discharge, the operation voltage drops rapidly from the opening circuit voltage (OCV) to 0.3 V, delivering a capacity of 278 mAh g^−1^ (3.5 Na^+^ per formula unit). This behavior is attributed to the formation of a solid electrolyte interphase (SEI) film and to pseudocapacitive processes. Subsequently, an irreversible plateau is observed between 0.3 to 0.01 V, corresponding to a capacity of 326.5 mAh g^−1^ (4.1 Na^+^ per formula unit). The average operation voltage for the subsequent profiles of FeNb_2_O_6_@C is a moderate value of ≈0.6 V, which is beneficial for achieving high energy density in a full cell, making it more promising for practical application. The clear peak (0.01–3 V) in the first cyclic voltammogram (CV) of FeNb_2_O_6_@C contributes to the amorphization process, which is consistent with the plateau region in the voltage profile (Figure S10, Supporting Information). In contrast, *T*‐Nb_2_O_5_@C only exhibits a weak peak (0.01–3 V) in the first CV cycle, which is responsible for the low capacity (Figure S10, Supporting Information). For comparison, we have also studied the Li‐ion storage behavior of FeNb_2_O_6_@C in half‐cells with Li foil anodes (Figure S11, Supporting Information). FeNb_2_O_6_ has been previously studied as an anode material for lithium‐ion batteries. P. B. Samarasingha et al.^[^ ^14f^ ^]^ reported that it delivered a limited initial capacity of ≈68 mAh g^−1^ and reversible capacity of 37 mAh g^−1^ in the voltage window of 0.5–3.0 V. FeNb_2_O_6_@C shows an initial capacity of 307.1 mAh g^−1^ and reversible capacity of 148.7 mAh g^−1^ (Figure S11a, Supporting Information). The voltage profiles and CVs show that the capacity contribution during the lithiation process occurs below 1.5 V. The intense peak at ≈0.7 V in the first CV curve is mainly attributed to the partial structural disordering, as revealed by *operando* XRD discussed below. The irreversible reduction peak at 0.85–1.0 V could be ascribed to the formation of an SEI film,^[^ ^14^, ^17^ ^]^ leading to the irreversible capacity decay of the initial first cycle. Similar behavior has been reported for other columbite Nb‐based oxides in LIBs. Feng et al.^[^ ^17a^ ^]^ have demonstrated that the CV curve of MNb_2_O_6_ (M = Ni, Cu, Zn) during the initial scan differs from that observed in subsequent cycles, primarily due to the development of the SEI film at a potential below 1.0 V. Cheng et al.^[^ ^14e^ ^]^ reported that a broad reduction peak appears at ≈0.75 V during the initial lithiation process of CdNb_2_O_6_, which the authors ascribed to the electrolyte decomposition and the formation of SEI. Additionally, the Li‐ion storage mechanism of FeNb_2_O_6_@C was probed via *operando* XRD within the voltage window 0.5–3.0 V (Figure S12, Supporting Information). During the lithiation process from OCV to 0.8 V, the position and intensity of the reflections in the XRD patterns do not change obviously. This behavior is consistent with the structural evolution observed for FeNb_2_O_6_@C from OCV to 0.3 V in SIBs, which has been attributed to pseudocapacitive processes and SEI layer formation below 1.0 V. Upon further lithiation from 0.8 to 0.5 V, a decrease in the intensity of the diffraction peaks is observed, while the peak positions remain constant. This may result from partial amorphization induced by lithium insertion, which is consistent with the reduction peak observed at 0.7 V in the CV. The absence of peak shifts suggests that no significant lattice expansion occurs within this voltage window. During subsequent cycling, the positions of the reflections remain unchanged. This behavior indicates the formation of a partial amorphous phase that acts as a host for pseudocapacitive reaction.

The rate capabilities of FeNb_2_O_6_@C and *T*‐Nb_2_O_5_@C are compared in Figure 2c, revealing that FeNb_2_O_6_@C exhibits superior rate performance. It shows reversible capacities of 207.0 and 180.4 mAh g^−1^ at current densities of 1 and 2 A g^−1^, respectively. When the current density is reduced to 0.02 A g^−1^ again, the capacity recovers to 312.9 mAh g^−1^. In contrast, *T*‐Nb_2_O_5_@C only delivers reversible capacities of 93.5 and 82 mAh g^−1^ at 1 and 2 A g^−1^, respectively. The electrochemical performance of *T*‐Nb_2_O_5_@C in this work was compared with that of previously reported Nb_2_O_5_‐based electrode materials to demonstrate its suitability as a reference for comparison with FeNb_2_O_6_@C (Table S6, Supporting Information). *T*‐Nb_2_O_5_@C exhibits a comparable initial reversible capacity and rate capability to those of mesoporous Nb_2_O_5_/C (175 mAh g^−1^ at 50 mA g^−1^, 60 mAh g^−1^ at 1 A g^−1^) and Nb_2_O_5_/C@NC (201 mAh g^−1^ at 100 mA g^−1^, 109 at 1 A g^−1^). Additionally, mesoporous Nb_2_O_5_/C and Nb_2_O_5_/C@NC display similar linear voltage profiles to that of *T*‐Nb_2_O_5_@C.^[^ ^11^, ^18^ ^]^ Compared to other previously reported intercalation‐type anode materials,^[^ ^11^, ^19^ ^]^ such as Nb‐based and Ti‐based oxides, FeNb_2_O_6_@C exhibits competitive electrochemical performance (Figure 2d). The long cycling stability of FeNb_2_O_6_@C and *T*‐Nb_2_O_5_@C is shown in Figure 2e and Figures S13–S15 (Supporting Information). FeNb_2_O_6_@C maintains a high capacity of 256 mAh g^−1^ with no capacity degradation during cycling at 0.2 A g^−1^, whereas *T*‐Nb_2_O_5_@C shows a specific capacity of 147.8 mA h g^−1^ after 500 cycles (Figure S13, Supporting Information). Although *T*‐Nb_2_O_5_@C delivers a lower capacity during long‐term cycling, it does not show significant capacity degradation, similar to other Nb_2_O_5_‐based anodes reported in the literature, such as Nb_2_O_5_/C@NC and a nanospherical Nb_2_O_5_/C composite.^[^ ^18^, ^20^ ^]^ Additionally, the long‐term cycling performance of FeNb_2_O_6_@C was evaluated at 1.0 A g^−1^ for 2000 cycles, achieving a capacity retention of 95.2% after 2600 cycles (Figure 2e). The capacity increases gradually during the initial ≈400 cycles, subsequently decreasing slowly until finally stabilizing after ≈1200 cycles. The initial capacity increase can be attributed to an electrochemical activation process.^[^ ^21^ ^]^ Figures S14–S17 (Supporting Information) show the rate capability and cycling stability of three independent cells measured for FeNb_2_O_6_@C and *T*‐Nb_2_O_5_@C. The results are consistent among the cells, demonstrating the reproducibility of the results. Electrochemical impedance spectroscopy (EIS) measurements were carried out to further investigate the electrode activation process. The Nyquist plots for FeNb_2_O_6_@C and *T*‐Nb_2_O_5_@C at different cycling stages are presented in Figure S18a,b (Supporting Information), respectively. The linear plots in the low‐frequency regions are indicative of typical Warburg behavior, which is associated with the diffusion of Na^+^ in the electrode.^[^ ^22^ ^]^ The gradual decrease of the Warburg coefficient *σ* for FeNb_2_O_6_@C and *T*‐Nb_2_O_5_@C during the initial cycling stages indicates enhancement of the Na^+^ ion kinetics (Figure S18c,d, Supporting Information), suggesting that the materials undergo electrochemical activation.

The electrochemical performance of FeNb_2_O_6_ and *T*‐Nb_2_O_5_ without carbon coating was also studied, and the results are presented in Figures S19 and S20 (Supporting Information). The results are qualitatively similar to those obtained for the carbon‐coated oxides. FeNb_2_O_6_ exhibits a high initial reversible capacity of 351.9 mAh g^−1^ at 0.02 A g^−1^ compared to 189.4 mAh g^−1^ for *T*‐Nb_2_O_5_. Moreover, FeNb_2_O_6_ shows superior rate performance, delivering capacities of 176.7 and 144.6 mAh g^−1^ at current densities of 1 and 2A g^−1^ (Figure S20, Supporting Information), respectively, whereas the capacity of *T*‐Nb_2_O_5_ remains below 35 mAh g^−1^ at the same current densities. Compared with previously reported Ti‐ and Nb‐based anodes, FeNb_2_O_6_ offers a high specific capacity along with a moderate voltage of ≈0.6 V, demonstrating its potential for SIB applications.

### 2.3 Na‐Ion Storage Mechanism

**Figure 3:**
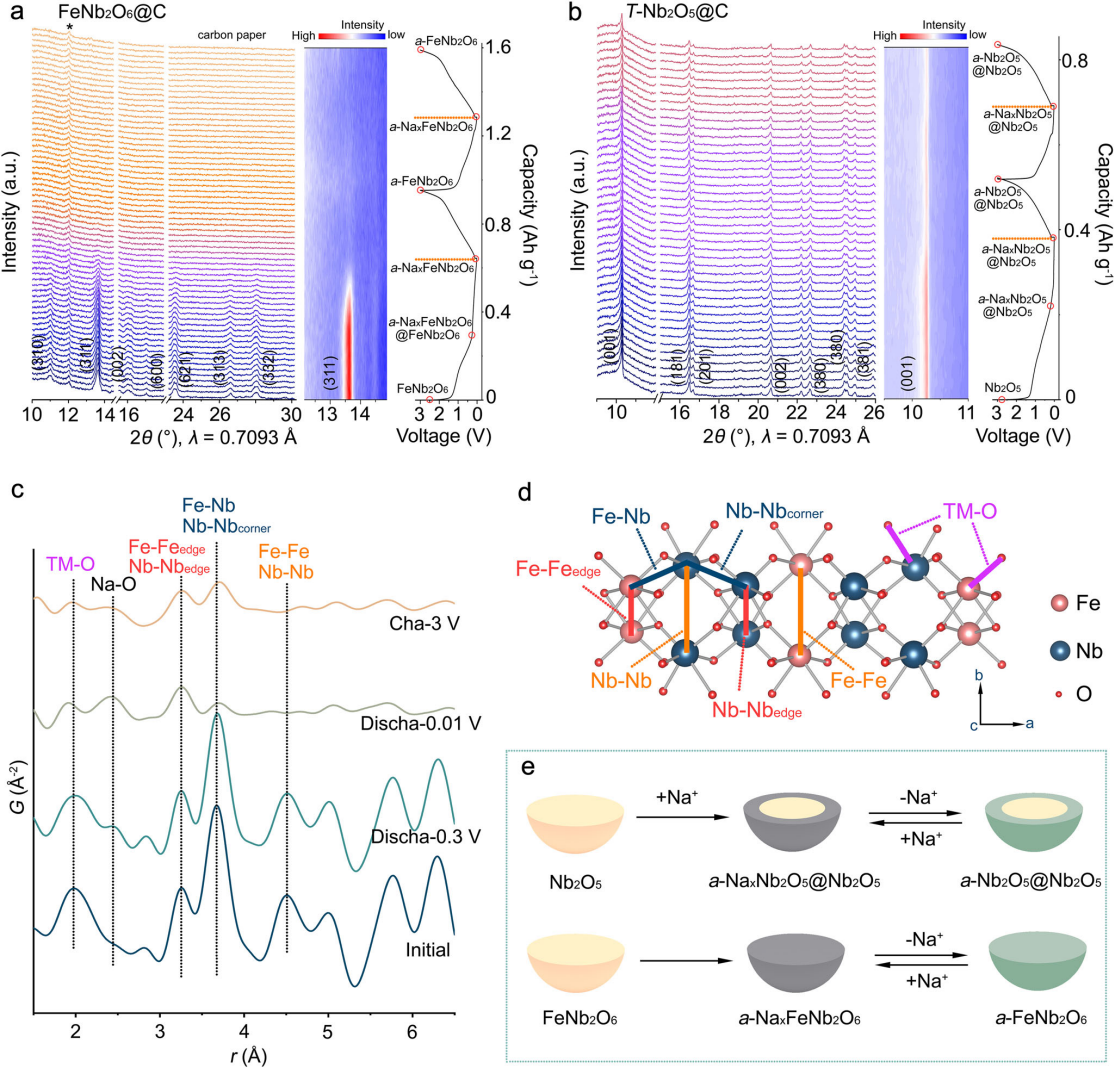
*Operando* XRD patterns of a) FeNb_2_O_6_@C and b) *T*‐Nb_2_O_5_@C with corresponding voltage profiles, collected for the first and second cycles. c) Ex situ PDF patterns of FeNb_2_O_6_@C at different voltage stages. d) Schematic illustration of the FeNb_2_O_6_ structure viewed along the *c* axis, with the atom pairs corresponding to the peaks in the PDFs labeled with the same colors. e) Schematic illustration of sodium‐ion storage mechanism in *T*‐Nb_2_O_5_@C and FeNb_2_O_6_@C.

Both the long‐range and local structures of FeNb_2_O_6_@C were investigated during the sodium‐ion storage process to better understand the electrochemical behavior of this material. To gain insights into the evolution of the long‐range structural order, *operando* XRD was performed within the voltage window 0.01–3.0 V. **Figure** **3**a,b shows the operando XRD patterns of FeNb_2_O_6_@C and *T*‐Nb_2_O_5_@C and the corresponding initial sodiation and de‐sodiation curves. The diffractogram of the pristine FeNb_2_O_6_@C exhibits reflections ascribed to the (310), (311), (002), (600), (621), (313), and (332) planes. The intensity and position of these reflections remain largely unchanged from OCV to 0.3 V, suggesting that the primary contributions to the capacity in this region are the SEI layer formation and surface‐redox pseudocapacitive processes. Upon further discharge to 0.01 V, which corresponds to the long plateau in the voltage profile, the intensities of the reflections progressively decrease and essentially vanish at the end of the discharge, coinciding with the insertion of 4.1 Na⁺ ions. Therefore, FeNb_2_O_6_ completely loses its long‐range order during the sodiation plateau, reaching an amorphous state (*a*‐FeNb_2_O_6_) by the end of the discharge process. This observation agrees with the ex situ XRD measurements performed on the discharged material (Figure S21, Supporting Information). During subsequent charging and the second cycle, FeNb_2_O_6_ remains amorphous, serving as an effective intercalation host for Na^+^ insertion and extraction while delivering high capacity. This finding is consistent with literature reports that an amorphization transition is essential for achieving high specific capacity in pseudocapacitive materials used as sodium insertion hosts.^[^ ^23^ ^]^

Similarly to FeNb_2_O_6_@C, the intensity of the reflections in the XRD patterns of *T*‐Nb_2_O_5_@C does not significantly change during discharge from OCV to 0.3 V, indicating that the degree of crystallinity of the oxide remains unchanged (Figure 3b). However, the intensity of the diffraction peaks only slightly decreases during the plateau from 0.3 to 0.01 V (which corresponds to 1.8 Na^+^ insertion), suggesting only a partial amorphization of the Nb_2_O_5_ oxide (*a*‐Nb_2_O_5_) in *T*‐Nb_2_O_5_@C, unlike the full amorphization observed for FeNb_2_O_6_@C after complete sodiation. During the charging process, the XRD patterns remain unchanged, reflecting that the material continues to be a mixture of crystalline *T*‐Nb_2_O_5_ and amorphous *a*‐Nb_2_O_5_. The limited sodium‐ion insertion in *T*‐Nb_2_O_5_@C explains its relatively small capacity in subsequent cycles. Ex situ HR‐TEM imaging of fully discharged FeNb_2_O_6_@C and *T*‐Nb_2_O_5_@C was performed to investigate the changes caused by the amorphization. After discharging to 0.01 V, *T*‐Nb_2_O_5_@C consists of a crystalline Nb_2_O_5_ core surrounded by a disordered Na_x_Nb_2_O_5_ surface shell (Figure S22, Supporting Information), showing that sodium is inserted only into the superficial region of the particles. On the contrary, fully discharged FeNb_2_O_6_@C particles become entirely disordered (Figure S23, Supporting Information), in agreement with the *Operando* XRD results, meaning that the sodium ions are inserted throughout the entire structure. The differences in Na^+^ storage between these two materials are schematically summarized in Figure 3e. Aberration‐corrected scanning transmission electron microscopy (AC‐STEM) was used to investigate the atomic arrangement of the iron and niobium in FeNb_2_O_6_ after initial discharge to 0.1 V (Figure S24, Supporting Information). This discharge voltage corresponds approximately to the midpoint of the long voltage plateau ranging from 0.01 V to 0.3 V, and was selected so that the material contained both crystalline and amorphous regions. The AC‐STEM image reveals that the particle is characterized by amorphous regions, located close to the surface, and crystalline regions, located mainly in the inner part of the particle. The high‐resolution fast Fourier transform (FFT) pattern shows the lattice oriented along the [100] crystallographic zone axis. Na^+^‐ion insertion into the crystalline structure leads to defects in the crystalline regions, prior to a complete amorphization, including extended defects such as stacking faults, as observed in Figure S24c (Supporting Information). The propagation of defects along specific crystallographic directions is consistent with the insertion of Na‐ions occurring along specific planes of atoms in the structure, as it will be discussed later in the text. The formation of extended defects, together with point defects and local disorder, in the crystalline regions can be seen as a transition stage between the highly crystalline initial structure and the entirely amorphous phase obtained after complete discharge. The EDS maps reflect the homogeneous distribution of sodium in FeNb_2_O_6_@C after sodiation (Figure S25, Supporting Information). To gain further insights into the changes that occur at the transition stage region between the crystalline and amorphous phases after sodium insertion, electron energy loss spectroscopy (EELS) analysis was performed of the sample discharged to 0.1 V. Figure S26 (Supporting Information) shows the O K‐ and Fe L_2,3_ edges EEL spectra measured from a higher crystallinity inner region to a more amorphized superficial region of a particle, as indicated in the corresponding TEM image (Figure S27, Supporting Information). The peak immediately above the edge onset in the O K‐edge spectra is associated with transitions of electrons from O 1s states to unoccupied states with 2p character. The peak is split due to the splitting of the d orbitals of the metals in the ligand field (the first and second peaks correspond respectively to the t_2g_ and e_g_ states) and to exchange splitting.^[^ ^24^ ^]^ The spectrum corresponding to the inner region of the particle is dominated by the contribution of NbO_6_ octahedra (d^0^), as the material contains more niobium than iron, with the iron bution overlapped.^[^ ^25^ ^]^ As the measurement moves toward the surface, some general changes occur in the spectra: a small decrease in the relative intensity of the first peak with respect to the second and a slight shift to higher energies; a broadening of the peaks; and a reduction of the energy difference between the peaks. These changes indicate a decrease in the number of unoccupied p‐character states, a broader energy distribution of those states, and a reduction of the ligand field splitting, along the transition region between the crystalline and amorphous parts of the structure, caused by the Na‐ion insertion. In the Fe L_2,3_‐edges EEL spectra, the L_3_/L_2_ intensity ratio decreases from 2 in the inner, more crystalline region to 1.75 at the surface, with ratios 1.78–1.80 in between (L_3_ and L_2_ correspond, respectively, to transitions from the 2p_3/2_ and 2p_1/3_ core levels to unoccupied d states of the metal). The decrease of the L_3_/L_2_ ratio indicates a reduction of the iron in the same direction,^[^ ^26^ ^]^ as sodium ions are introduced into the structure. The EELS results suggest that in the transition region between the crystalline and the amorphous regions, the insertion of sodium causes a reduction of the niobium and iron, accompanied by a broadening of the variation of the hybridization degree of the O 2p orbitals with the Nb and Fe d orbitals, which reflects the progressive increase in the disorder around the inserted sodium ions. The results indicate that an amorphous phase is more readily formed for FeNb_2_O_6_, suggesting that the presence of Fe in the structure is essential for promoting a complete amorphization. As a result, additional Na^+^ sites are available within the FeNb_2_O_6_ host compared to *T*‐Nb_2_O_5_, leading to a higher specific capacity.

To gain further insight into the structural changes in FeNb_2_O_6_@C during sodiation and de‐sodiation, PDF analysis was performed at various stages of discharge and charge. Figure 3c shows the short‐range regions (*r* < 6.5 Å) of the PDF patterns for FeNb_2_O_6_@C at different voltage states. The atomic pairs corresponding to the observed peaks are indicated in matching colors on the FeNb_2_O_6_ structure shown in Figure 3d. To help elucidate the changes in the PDFs, patterns were calculated based on the FeNb_2_O_6_ crystallographic data for each atomic pair in the structure (Figure S28, Supporting Information), namely Fe‒O, Nb‒O, Fe‒Fe, Nb‒Fe, and Nb‒Nb. The first peak at 1.97 Å in the initial PDF corresponds to the overlapping Fe‒O and Nb‒O interatomic distances in the FeO_6_ and NbO_6_ octahedra, respectively. The peak at 3.26 Å corresponds to distances between pairs of Fe atoms and pairs of Nb atoms (Fe‒Fe_edge_ and Nb‒Nb_edge_, respectively) in edge‐sharing FeO_6_‒FeO_6_ and edge‐sharing NbO_6_‒NbO_6_ octahedra. The peak at 3.67 Å is associated with the Fe‒Nb and Nb‒Nb_corner_ interatomic distances in corner‐sharing FeO_6_‒NbO_6_ and NbO_6_‒NbO_6_ octahedra. Finally, the peak at 4.51 Å is attributed to Fe‒Fe and Nb‒Nb pairs from FeO_6_‒FeO_6_ and NbO_6_‒NbO_6_ positioned on opposite sides of the tunnels formed by the octahedra of the respective metal atoms. A new peak at 2.4 Å, assigned to the Na‒O distance, appears in the PDF of the material discharged to 0.3 V, while no other changes are observed. This indicates that sodium is inserted into the crystal structure from OCV to 0.3 V, but the amount of sodium inserted does not affect the structure, in agreement with the *operando* XRD results. A significant decrease in the intensity and an increase in the broadening of the TM‒O peak are observed in the PDF of the material fully discharged to 0.01 V, reflecting a broad variation in the Fe‒O and Nb‒O bond lengths in the octahedra, typical of structural disorder. However, only a slight decrease in the intensity of the Fe–Fe_edge_/Nb–Nb_edge_ peak is observed after full sodiation, suggesting that some short‐range order is maintained within the zigzag chains formed by the Fe and Nb octahedra. The partial retention of the short‐range order in the zigzag chains results from the strong interaction between adjacent corner‐sharing octahedra, which may be essential for providing and maintaining a high number of accessible sites for Na^+^ ions. In contrast, the intensity of the neighboring Fe–Nb/Nb–Nb_corner_ peak decreases more drastically, reflecting a loss of regularity between the Fe–Fe and Nb–Nb planes along the *a* axis during the amorphization process. In addition, the drastic decrease in the intensity of the Fe–Fe/Nb–Nb peak is attributed to the anisotropic disruption of the regularly arranged zigzag chains of Fe and Nb octahedra following the insertion of Na^+^ ions into the tunnels. These results suggest that the Na^+^ ions are inserted into the structure along the *c* axis, disrupting the ordered arrangement of the chains. Upon charging to 3.0 V, the intensity of the Na‒O peak decreases as Na^+^ ions are de‐inserted from the amorphous host. Moreover, the intensity of the Fe–Nb/Nb–Nb_corner_ peak slightly increases, revealing a slight increase in the structural order between the Fe–Fe and Nb–Nb planes after sodium extraction. The long‐range regions of the PDF patterns (*r* >6.5 Å) lose all atomic pair correlations after discharge to 0.01 V, demonstrating the irreversible loss of long‐range structural order caused by the amorphization process (Figure S29).

**Figure 4:**
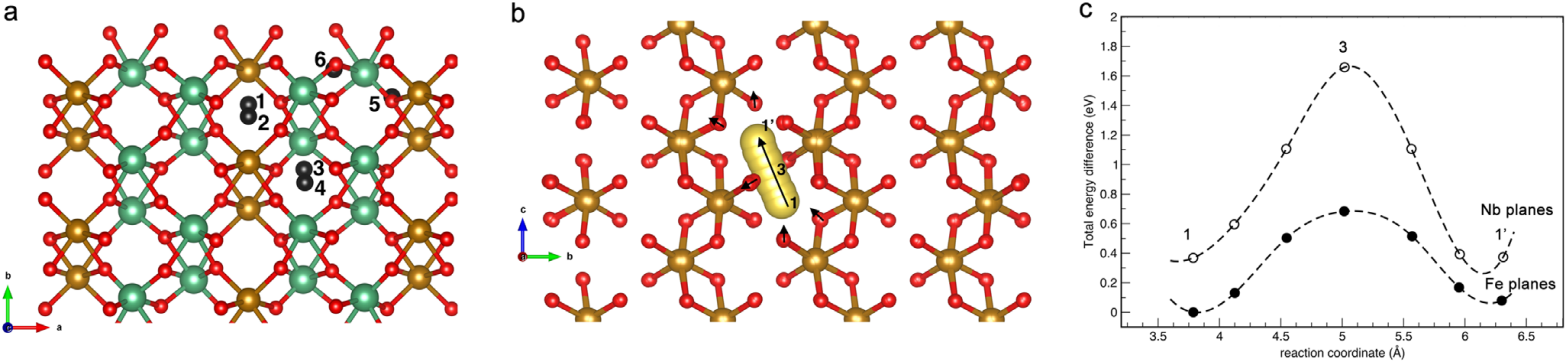
a) Positions of interstitial Na^+^ ions sites in FeNb_2_O_6_; b) overlayered atomic positions of minimum energy migration path for Na^+^ ions, with black arrows indicating the direction of atomic relaxation of neighbroing O atoms as Na atom moves from 1 to 1′ over the saddle point 3. c) Energy barriers of the images along the NEB paths in Fe (filled circles) and Nb (empty circles) *bc* planes.

To provide understanding of the initial insertion of sodium ions in crystalline FeNb_2_O_6_, ab initio calculations within Density Functional Theory were performed.^[^ ^27^ ^]^ Starting from the relaxed geometry of FeNb_2_O_6_ at the experimental unit cell volume, we calculated formation energies for isolated the Na^+^ ions in the bulk oxide (1 Na^+^ atom in 8 f.u., Figure S30, Supporting Information). We have considered the possible interstitial sites depicted in **Figure** **4**a, for which total energies were calculated after minimization of the atomic forces: interstitial sites between zig‐zag chains of Fe or Nb octahedra (1,2,3,4 in the figure) and between FeO_6_‐NbO_6_ or NbO_6_‐NbO_6_ octahedra belonging to adjacent *bc* planes (5,6). DFT calculations show that isolated Na^+^ ions preferentially occupy the void site between adjacent zigzag chains of FeO_6_ octahedra within Fe *bc* planes (site 1 in Figure 4a, corresponding relaxed atomic configuration are reported in Figure S31a, Supporting Information), while the corresponding site in the NbO_6_‐planes (site 2, in Figure S31b, Supporting Information) is slightly higher in energy by 0.37 eV. Placing a Na^+^ ions in the other interstitials induces larger local distortions, leading to higher configuration energies (greater than 1.5 eV for sites 5 and 6, with local Fe/Nb‐Nb_corner_ distances changing by ±1 Å) or spontaneously relaxing (sites 3,4) toward the closer minimum energy configurations (1,2). The investigation of Na^+^ ion migration path along *c*‐oriented void channels in Fe (Nb) planes was performed via DFT‐Nudged Elastic Band (NEB) calculations^[^ ^28^ ^]^ and returns an estimate for a migration barrier *E*_m_ = 0.68 eV (1.2 eV), for diffusion between two neighboring void sites in a *bc* Fe (Nb) plane (Figure 4c). In Figure 4b, the atomic configurations of the NEB images were overlayered to emphasize the relaxation of the FeO_6_ octahedra and the path of the Na^+^ ions. The black arrows on the O atoms highlight the displacement (≈0.1 A) of the nearest‐neighboring oxygens as Na moves from one minimum to the next across the saddle point (3). Migration paths along *a* or *b* directions, i.e., from one *bc* plane to the adjacent one or crossing a FeO_6_ or NbO_6_ zig‐zag chain, were not investigated, as the calculated energetics of the possible saddle configurations (5,6, see above) already indicate these paths as highly unfavorable. Overall, DFT calculations show a preference for Na^+^ ions to be included in the interstitial voids between zigzag chains, in particular within Fe planes. As a further indication, the calculated energetics for two Na^+^ atoms in the supercell reveal a preference for the atoms to be close to each other on the Fe plane, rather than being positioned both in a Nb plane or across planes (ΔE = 0.32 eV/Na atom).

**Figure 5:**
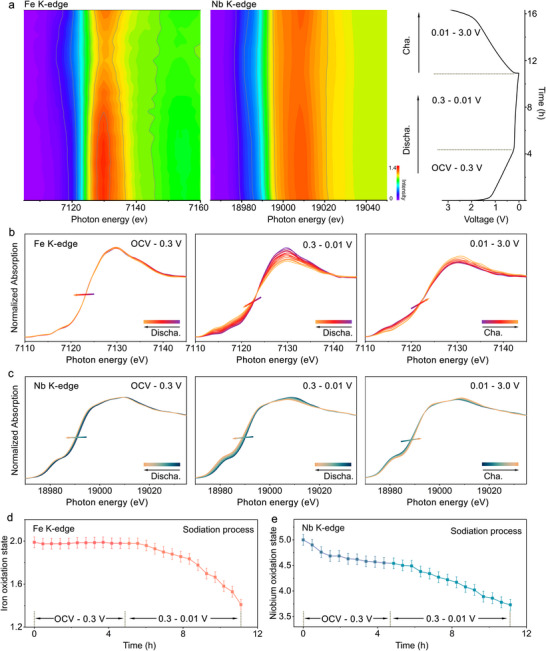
a) *Operando* Fe K‐edge and Nb K‐edge XANES contour plots of FeNb_2_O_6_@C during sodiation and desodiation (the corresponding voltage profile for the first cycle is shown on the right side). *Operando* b) Fe K‐edge and c) Nb K‐edge XANES spectra of FeNb_2_O_6_@C for the different regions in the voltage profile. Oxidation state of d) Fe and e) Nb as a function of time during sodiation.

**Figure 6:**
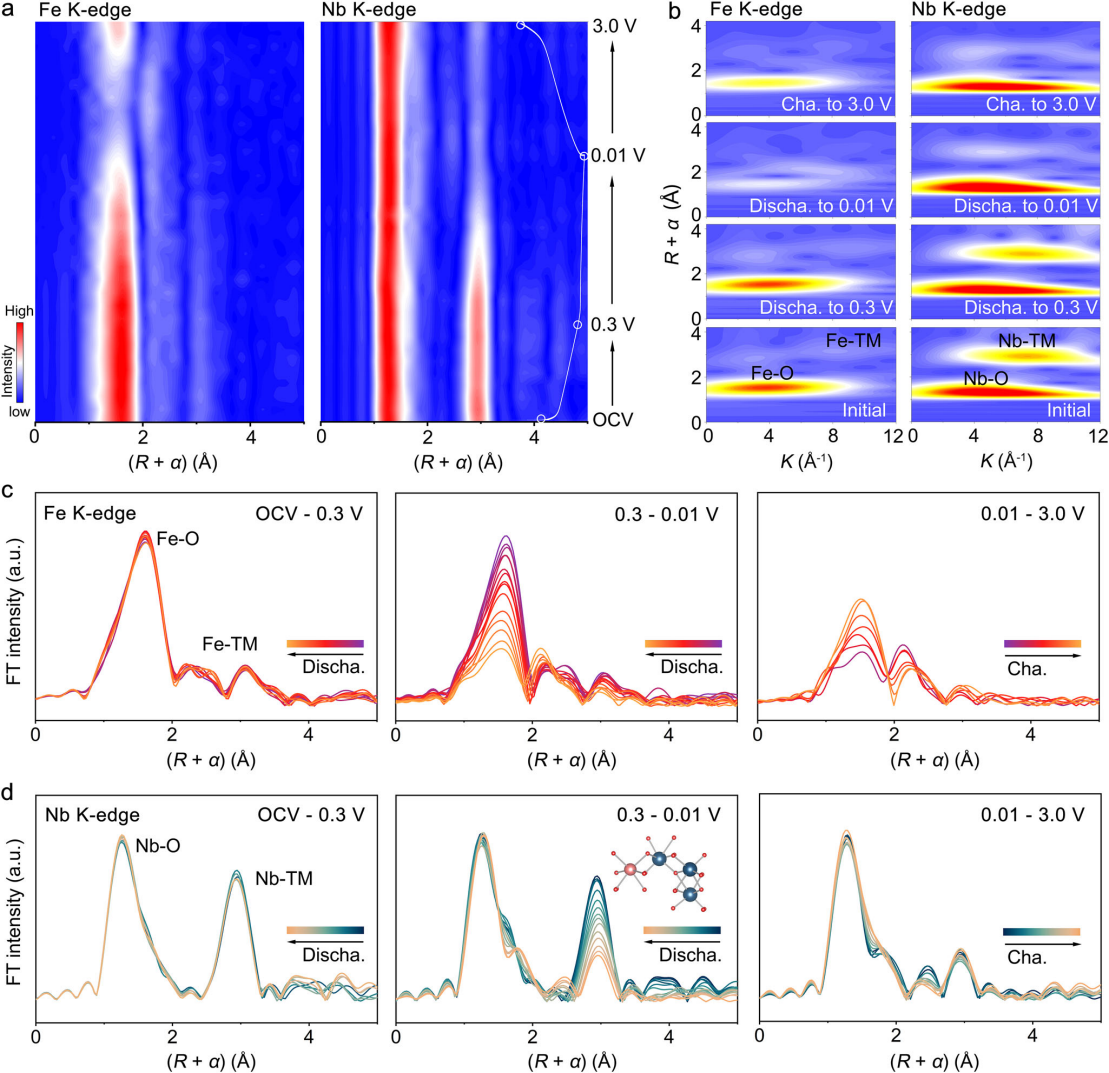
a) *Operando* Fe K‐edge and Nb K‐edge EXAFS contour plots of FeNb_2_O_6_@C during sodiation and desodiation (the corresponding voltage profile for the first cycle is shown on the right side). b) WT‐EXAFS spectra of FeNb_2_O_6_@C at different voltage stages (the blue–yellow–red color gradient represents the increasing intensity of the EXAFS signal). *Operando* c) Fe K‐edge and d) Nb K‐edge EXAFS spectra of FeNb_2_O_6_@C for the different regions in the voltage profile.

Information on the charge‐compensation process and the evolution of the local structure around the Fe and Nb atoms during sodiation and de‐sodiation was obtained via *operando* Fe and Nb K‐edge XANES and EXAFS analyses (**Figures** **5** and **6**). The XANES contour plots for the first cycle corresponding to the Fe and Nb K‐edge spectra of FeNb_2_O_6_@C (cycled at 40 mAh g^−1^ within the voltage window of 0.01–3 V), show different trends of the Fe and Nb absorption edges across different regions of the voltage profile (Figure 5a). Based on this observation, the spectra were separated into three different groups corresponding to the voltage ranges from OCV to 0.3 V, 0.3 to 0.01 V, and 0.01 to 3 V (Figure 5b,c). The oxidation states of the Fe and Nb during discharge were calculated from the edge energy, using the half‐height method, and are presented in Figure 5d,e. The position of the Fe absorption edge hardly changes during discharge from OCV to 0.3 V, and its oxidation state remains close to 2+ (Figure 5d), revealing that the iron cations do not participate in the electrochemical redox reactions in this voltage range. On the contrary, the Nb absorption edge shifts to lower energies during discharge from OCV to 0.3 V, indicating the reduction of the metal. The oxidation state initially decreases rapidly, followed by a more gradual reduction to 4.5+ (Figure 5e), which corresponds to the insertion of 1.0 Na^+^ per formula unit, and suggests that Nb is preferentially reduced within this voltage range. Therefore, in addition to the formation of SEI film and surface‐redox pseudocapacitive processes, the intercalation reaction contributes to the capacity. During the subsequent amorphization process within the voltage range of 0.3 to 0.01 V, significant changes are observed in the Fe K‐edge XANES spectra. The absorption edge shifts to lower energies during discharge, and the oxidation number of Fe decreases down to 1.4+ in the fully discharged state. The oxidation state of Nb decreases consistently to 3.7+ in the same voltage range. This corresponds to an overall insertion into the lattice of 2.2 Na^+^‐ions per formula unit. During the charging process, the Fe and Nb absorption edges shift to higher energies, corresponding to an increase in their oxidation states to 1.7+ and 4.4+, respectively. Therefore, the reversible insertion and extraction of Na^+^‐ions in FeNb_2_O_6_@C occurs via redox reactions that involve a change in the oxidation state of Fe from 1.7+ to 1.4+ and of Nb from 4.4+ to 3.7+, which corresponds to 1.7 Na^+^‐ions per formula unit. Consequently, 1.5 Na^+^ ions remain intercalated in FeNb_2_O_6_@C and are not extracted, which contributes to the irreversible capacity observed during the initial cycle.

*Operando* FT‐EXAFS spectra were used to evaluate the evolution of the local structure and coordination environment around the Fe and Nb centers during sodiation and de‐sodiation (Figure 6a). Similarly to the operando XANES analysis, the spectra were divided into three groups corresponding to distinct regions of the voltage profile: discharge from OCV to 0.3 V, discharge from 0.3 to 0.01 V, and the charging process (Figure 6c,d). The peaks at 1.6 Å and 3.1 Å in the Fe K‐edge EXAFS spectra are associated with the first (Fe–O) and second (Fe–TM) coordination shells, respectively. Similarly, in the Nb K‐edge EXAFS spectra, the peaks at 1.4 Å and 3.0 Å are related to the Nb‒O and Nb‒TM first and second coordination shells around the niobium atoms, respectively. During discharge from OCV to 0.3 V, no drastic alterations are observed in the Fe and Nb spectra in terms of peak position or intensity. This is consistent with the ex situ PDF analysis and operando XRD results, which also show no important changes. Niobium is reduced within this voltage range, but this change in oxidation state does not appear to affect its local structure. The results suggest that the insertion of 1.0 Na^+^ ions per formula unit into the structure tunnels above 0.3 V does not alter both the local and long‐range structural order of FeNb_2_O_6_. During discharge from 0.3 to 0.01 V, which is the voltage range associated with the amorphization process, the intensity of the Fe‒O shell peak drops rapidly. Additionally, both the position and intensity of the Fe‒TM peak change. These alterations suggest a rapid increase in the disorder within the oxygen coordination shell around the metal atom, which affects its metal sublattice. In contrast, the Nb‒O peak intensity remains almost unchanged during this discharge stage, indicating that the coordination environment around the niobium is largely preserved. Thus, despite the structure losing its long‐range order during discharge from 0.3 to 0.01 V, the oxygen coordination environment around the niobium remains nearly intact. The intensity of the Nb‒TM peak, on the other hand, decreases due to the disorder introduced into the metal sublattice, but the peak is partially retained after full sodiation. Taking into account the ex situ PDF results, the Nb‒TM peak observed after full discharge is attributed to the retention of short‐range structural integrity in the Nb–Nb_edge_ pairs within the zigzag chains, while the Fe–Nb/Nb–Nb_corner_ pairs are affected by the strong disorder across the Fe and Nb planes. During the subsequent desodiation process, the intensity of the Fe‒O peak gradually increases, eventually reaching half the height of the Fe–O peak in the OCV‐state, suggesting some flexibility of the FeO_6_ octahedra during Na^+^‐ion insertion and extraction. Meanwhile, the Nb–O and Nb–TM peaks remain almost unchanged from the fully sodiated state throughout the entire charging process. The ex situ FT‐EXAFS spectra of *T*‐Nb_2_O_5_@C (Figure S32, Supporting Information) show decreased intensity of the Nb‒O and Nb‒Nb peaks, in agreement with the partial local disorder in the structure due to the amorphous phase formed at the surface of the particles. The absence of FeO_6_ structural units in *T*‐Nb_2_O_5_@C results in an incomplete amorphization process.

Wavelet transform analysis of the Fe and Nb K‐edge EXAFS spectra was conducted to further show the evolution of the coordination environment of the metals (Figure 6b). The scattering peaks associated with Fe–O, Fe–TM, Nb–O, and Nb–TM remain almost unchanged during sodiation from OCV to 0.3 V. In the fully sodiated state, the sharp decrease in the intensity of both Fe‒O and Nb‒TM scattering peaks indicates that strong distortion occurs within the FeO_6_ octahedra, while a disordered structure forms between corner‐shared NbO_6_ octahedra and adjacent NbO_6_‐FeO_6_ octahedra. The results suggest that the short‐range structural order remaining in the NbO_6_ zigzag chains after the first full sodiation forms a “skeleton” that enables the material to maintain abundant active sites and suitable Na^+^‐ion diffusion pathways, facilitating intercalation and surface‐redox pseudocapacitance reactions during subsequent cycles.

The data demonstrates the Na‐ion storage mechanism in FeNb_2_O_6_@C. Specifically, it indicates that the initial sodiation process from 0.3 to 0.01 V functions as an “activation process” of FeNb_2_O_6_ for reversible Na storage. During this process, long‐range disorder is induced by the local disorder of flexible FeO_6_ octahedra in FeO_6_ *bc* planes. DFT results suggest that Na^+^ ions preferentially occupy the void site between adjacent zigzag chains within FeO_6_ *bc* planes, which promotes the disorder of FeO_6_. Along the *a* axis, the corner‐shared NbO_6_ and FeO_6_ octahedra show a loss of regularity. In contrast, the local structure of the NbO_6_ octahedra is maintained during this amorphization, and the edge‐shared NbO_6_ octahedra in zigzag chains along NbO_6_ *bc* planes retain local‐range order. This is attributed to the structural rigidity of NbO_6_ octahedra and greater flexibility found in corner‐sharing polyhedra compared to edge‐sharing polyhedra. As a result, an amorphous structure along with local NbO_6_ ordering forms after the initial sodiation, which makes FeNb_2_O_6_@C a stable host for fast Na^+^‐ion intercalation during subsequent cycles.

## 3 Conclusion

FeNb_2_O_6_ has been used here for the first time as an anode material for SIBs. We found that it possesses a specific Na^+^‐ion storage mechanism that is unlike those in typical transition‐metal oxide anodes. FeNb_2_O_6_@C delivers a practical reversible capacity of 304.9 mAh g^−2^, showing a sloping discharge and charge profile with a moderate average voltage of ≈0.6 V, which enhances the safety of the battery. Additionally, it exhibits a high rate capability and is stable during cycling up to 2600 cycles. The excellent Na‐storage properties stem from the intrinsic properties of the structure. Specifically, structural amorphization can be triggered by the disorder introduced into the FeO_6_ octahedra during the initial sodiation. This is accompanied by short‐range order within the NbO_6_ planes. The resulting structure provides rich active Na^+^ sites for pseudocapacitive intercalation and facile ion diffusion pathways. This study proposes a new type of anode material for SIBs and provides guidance for an in‐depth understanding of the sodium storage mechanism in transition‐metal oxide materials, and therefore will promote the development of SIBs.

## Conflict of Interest

The authors declare no conflict of interest.

## Supporting information



Supporting Information

## Data Availability

The data that support the findings of this study are available from the corresponding author upon reasonable request.
