# Supplementary material for: FeNb2O6 as a High‐Performance Anode for Sodium‐Ion Batteries Enabled by Structural Amorphization Coupled with NbO6 Local Ordering
Source: Adv Mater. 2025 Jul 29;37(46):e04100. doi: 10.1002/adma.202504100 (PMC12631508; doi:10.1002/adma.202504100)
Supplement: Supplementary file 1 — Supporting Information [file ADMA-37-e04100-s001.docx]

Supporting Information

FeNb_2_O_6_ as a high-performance anode for sodium-ion batteries enabled by structural amorphization coupled with NbO_6_ local ordering

Yanchen Liu^1^, Ana G. Buzanich^2^, Paola Alippi^3^, Luciano A. Montoro^4^, Kug-Seung Lee^5^, Taeyeol Jeon^5^, Kilian Weißer^1^, Martin A. Karlsen^6^, Patrícia A. Russo^1^*, Nicola Pinna^1^*

1 Department of Chemistry and The Center for the Science of Materials Berlin, Humboldt-Universität zu Berlin, Brook-Taylor-Str. 2, 12489 Berlin, Germany

2 Bundesanstalt für Materialforschung und -prüfung (BAM), Richard-Willstätter-Straße 11, 12489, Berlin, Germany

3 CNR-ISM, Consiglio Nazionale delle Ricerche, Istituto di Struttura della Materia, Via Salaria Km 29.3, I-00015 Monterotondo Stazione, Roma, Italy

4 Universidade Federal de Minas Gerais, Department of Chemistry, Belo Horizonte, MG 31270-901, Brazil

5 PLS-II Beamline Depart. / Pohang Accelerator Laboratory, POSTECH, 80 Jigokro-127-beongil, Nam-gu Pohang, Gyeongbuk 37673, Korea

6 P02.1 Beamline, PETRA III Synchrotron, DESY, Notkestr. 85, 22607 Hamburg, Germany

**Experimental sections:**

*Material synthesis*: Firstly, K_7_HNb_6_O_19_·13H_2_O was prepared based on a method reported in the literature^1^. 3.325 g of Nb_2_O_5_ and 6.5 g of KOH were mixed with a mortar and placed into a nickel crucible. The mixture was calcined in a muffle oven at 480 ℃ for 50 min. The product was dissolved in 25 mL of boiling water, and then the water was evaporated from the suspension through heating. When the volume of solution reached about 12.5 mL, the mixture was centrifuged. The supernatant was placed in a refrigerator at 0 ℃ overnight, so that needle-like crystals were recrystallized. The obtained crystals were washed with 1:1(V/V) ethanol-water and absolute ethanol, and subsequently dried at 60 ℃ in a vacuum oven. FeNb_2_O_6_ and Nb_2_O_5_ were synthesized by hydrothermal method. For FeNb_2_O_6_, 0.167 g of FeSO_4_·7H_2_O and 0.274 g of K_7_HNb_6_O_19_·13H_2_O were separately dissolved in 10 mL of ultrapure water. Then, FeSO_4_ solution was added dropwise into the polyoxoniobate solution under rigorous magnetic stirring. Subsequently, 10 mL of ultrapure water was added into the obtained suspension, and the mixture was transferred into a Teflon-lined stainless-steel autoclave. For Nb_2_O_5_, 1.890 g of ammonium niobate oxalate was dissolved in 30 mL ultrapure water under magnetic stirring, then the solution was transferred to a Teflon-lined stainless-steel autoclave. The autoclaves containing the FeNb_2_O_6_ and Nb_2_O_5_ reaction mixtures were sealed and heated at 180 ℃ for 12 h, and then cooled to room temperature naturally. The solids were collected by centrifugation, washed with ethanol and ultrapure water several times, and dried at 60 ℃ overnight. The dried powders were calcined at 700 ℃ for 3 h in Ar atmosphere. 60 mg of FeNb_2_O_6_ or Nb_2_O_5_ were dispersed in 40 mL of Tris-buffer solution (10 mM) with sonication for 10 min. 80 mg of dopamine hydrochloride was added into the suspension, and then stirred at room temperature for 24 h. The solids were collected by centrifugation, washed with ultrapure water, and dried at 60 ℃ in a vacuum oven. The dopamine-coated FeNb_2_O_6_ and Nb_2_O_5_ were annealed at 700 ℃ for 3 h under Ar atmosphere.

*Electrochemical measurements*: Electrochemical measurements were performed in coin cells (CR2032, Shandong Gelon Lib Co., Ltd), which were assembled in an Ar-filled glove box. The electrode was prepared by mixing the active materials, super P and poly(vinyl difluride) (PVDF) in N-methyl-2-pyrrolidone with a weight ratio of 7:2:1 onto Cu/C foil, then dried at 90 ℃ in vacuum for 12 h. The loading mass of active materials in each electrode is 1.5-2.5 mg cm^-2^ in a diameter of 12 mm. Sodium foil was used as negative electrode, and glass fiber was used as separator. The electrolyte was 1.0 M of NaPF_6_ in ethylene carbonate/dimethyl carbonate (EC/DMC) in a volume ratio of 1:1 with 5 wt% fluoroethylene carbonate (FEC). The charge/discharge performance was conducted on a Land CT2001A battery test system in a voltage range of 0.01-3.0 V. CV was performed at various scan rates on a Bio-Logic VMP3 multichannel potentiostat/galvanostat. Li-ion storage behavior was tested in half-cells with Li foil as negative electrode. 1.0 M LiPF_6_ in ethylene carbonate/diethyl carbonate/dimethyl carbonate (EC/DEC/DMC) in a volume ratio of 1:1:1 was used as the electrolyte. The charge/discharge performance was conducted on a Land CT2001A battery test system in the voltage range of 0.5-3.0 V.

The theoretical capacity values for FeNb_2_O_6_ and Nb_2_O_5_ were calculated based on the equation below:

$$\text{C}_{\text{theoretical}}\text{ = }\frac{\text{nF}}{\text{3.6}\text{m}}$$

where *n* represents the number of electrons transferred per formula unit, *F* is the Faraday constant (96485.3 C mol^-1^), 3.6 is the conversion factor between coulomb and milliampere-hour, and *m* represents the mass per formula unit. The obtained theoretical values for FeNb_2_O_6_ and Nb_2_O_5_ are 238.2 and 201.7 mAh g^-1^, respectively, which is defined by one-electron transfer per transition metal.

*Materials characterization*: The XRD measurements were carried out at beamline P02.1, PETRA-III at DESY (Deutsches Elektronensynchrotron) in Hamburg, Germany, using synchrotron radiation (*λ* = 0.20737 Å) and on a STOE MP diffractometer using Mo Kα radiation (*λ* = 0.70930 Å). Rietveld refinement was conducted with the GSAS-Ⅱ software^2^. The Raman spectra were collected on an XPLORA plus Raman microscope with a 532 nm laser. Transmission electron microscopy (TEM), high-resolution TEM (HR-TEM), high-angle annular dark-field scanning transmission electron microscopy (HAADF-STEM) and energy dispersive X-ray spectroscopy (EDX) elemental mapping were carried out on a FEI Talos F200S scanning/transmission electron microscope (S/TEM) at an acceleration voltage of 200 kV. Aberration-corrected STEM (AC-STEM) analysis was performed using a FEI Titan Themis Cubed STEM microscope (FEI Company), equipped with a Cs probe corrector and operated at 300 kV. HAADF images were acquired with collection angles between 66–200 mrad to improve Z-contrast imaging. EELS was performed using a JEOL JEM-2100F microscope equipped with a Gatan Imaging Filter Tridiem 863, operating at 200 kV, STEM mode, 0.7 nm spot size, 2 cm camera length, 0.2 eV/pixel dispersion, 4 s/pixel acquisition time, 2048 channels, and 94 frames. The electrodes for ex situ AC-STEM and EELS were washed with dimethyl dimethyl carbonate (DMC) for three times, and then dried in vacuum. *Operando* XRD patterns were performed on a STOE MP diffractometer using Mo Kα radiation (λ = 0.70930 Å). Pouched coin cells with quartz glass sheet windows were assembled for *operando* XRD measurements. PDF measurements were carried out at beamline P02.1, PETRA-III at DESY (Deutsches Elektronen-Synchrotron) in Hamburg, Germany, using synchrotron radiation (λ = 0.207371 Å). The data was collected on a detector of Varex (150 × 150 µm2 pixel size, 2798.926 × 2807.232 pixel area). The detector was in 'corner configuration', i.e., the beam center was at the lower right corner of the detector, looking from the samples and downstream towards the detector. LaB6 was used for calibration of the experimental geometry. The pyFAI software was used for both calibration of the experimental geometry and for azimuthal integration. The sample-to-detector distance (SDD) was calibrated to be 300.046 mm. The Fourier transformation was conducted using the PDFgetX3 algorithm (*Q*_max_ = 18.0 Å^-1^) through the xPDFsuite software^3-5^. XAFS spectra were collected in transmission mode. The acquired spectra were extracted, calibrated, and normalized using the Athena software. The Fourier Transformations are made in k-space between 2 and 12 Å^-1^ for Fe K-edge, and 2 and 12 Å^-1^ for Nb L_III_-edge. The resulting Fe and Nb K-edge R-space are used for fitting with the model of orthorhombic FeNb_2_O_6._

*Operando* X-ray absorption spectra at the Fe K-edge (7112 eV) and Nb K-edge (18986 eV) were collected at the BAM*line* of the BESSY-II (Berlin, Germany)^6^, operated by the Helmholtz-Zentrum Berlin of Materials and Energy. The incident X-ray beam was provided by a super bend magnet (7 T) source and subsequently energetically narrowed by Double Crystal Monochromator (DCM), with Si (111) crystals, which is used to fine scan the energy range and an intrinsic resolution of ΔE/E=2x10^-4^. The beam size on the sample was 4 mm (horizontal) x 2 mm (vertical). The XAS measurements were performed in transmission mode containing both X-ray absorption near edge structure (XANES) and extended X-ray absorption fine structure (EXAFS). Pouched coin cells with Kapton film windows were assembled for *operando* XAS measurements. The pouched coin cell was placed between two Argon-filled ionization chambers (I_0_, I_1_, 5 cm and 15 cm long, respectively). The electrochemical tests were performed at 40 mA g^-1^ on a Bio-Logic VMP3 multichannel potentiostat/galvanostat. Fe and Nb metal foils were used to calibrate the energy at the respective energies. The measurement protocol was the following: 10 eV steps until 20 eV before the edge, followed by 0.5 eV steps until 20 eV above the edge and 2 eV steps until 200 eV above the edge. From then on equidistant k-steps were taken every 0.04 Å until 16 Å. The acquired spectra were extracted, calibrated, and normalized using the Athena and Larch software^7^. The Fourier Transformations are made in k-space between 2 and 12 Å^-1^ for Fe K-edge, and 2 and 12 Å^-1^ for Nb L_III_-edge. The resulting Fe and Nb K-edge R-spaces are used for fitting with the model of orthorhombic FeNb_2_O_6_. The FT EXAFS plots are the result of this.

*DFT calculations*: Density Functional Theory calculations have been performed within the plane-waves, projector augmented wave (PAW) method^8,9^ implemented in the Vienna ab initio simulation package (VASP)^10-12^ and within the generalized gradient approximation (GGA) with the Perdew-Burke-Ernzerhof parametrization^13^ for exchange-correlation interaction. We have applied a DFT+U Hubbard-like correction method, following the simplified rotationally invariant form of Dudarev *et al.* ^14^: a value of U_Fe-d_= 5.3 eV has been set, as in Ref.^23^. We have used a 400 eV cut-off for the plane wave expansion and a (2x4x4) k-point sampling of the Brillouin zone in the Monkhorts-Pack scheme. The primitive unit cell of FeNb_2_O_6_ consists of 4 formula units (i.e., formula Fe_4_Nb_8_O_24_), with 6 planes of NbO_6_ and FeO_6_ octahedra (alternate in the Fe-Nb-Nb-Fe-Nb-Nb) and 2 octahedra per plane. We have kept the unit cell dimension at the experimental ones. Spin-polarized calculations have been performed for FeNb_2_O_6_: differences between calculated total energies for distinct magnetic configurations are very small (1<meV/Fe atom), albeit indicating a preferential AFM ordering, as expected for columbite materials^15^. In particular, the AFM-1 arrangement (anti-ferro ordering of Fe atoms in a (b,c) plane and in a direction) is predicted to be the lowest energy one. The calculated magnetic moment on Fe atoms, calculated from projected spin-polarized charges, is μ_Fe_=3.77 μ_B_, with negligible spin-polarization of Nb and O atoms. To calculate total energies for Na insertion in FeNb_2_O_6_, we have doubled the unit cell in b and c directions and used a (2x2x2) k-point sampling. Atomic forces are calculated and geometrical minimization is performed until forces are lower than 0.01 eV/Å. We also observed that the calculated relative energetics of Na^+^ in different interstitial positions does not depend on the magnetic ordering of the host, and present here results for non spin-polarized calculations. To find the saddle point and the minimum energy path for Na^+^ migration between two neighboring interstitial sites, we have performed Nudged Elastic Band calculations^16^ with 5 intermediate images between the end points.


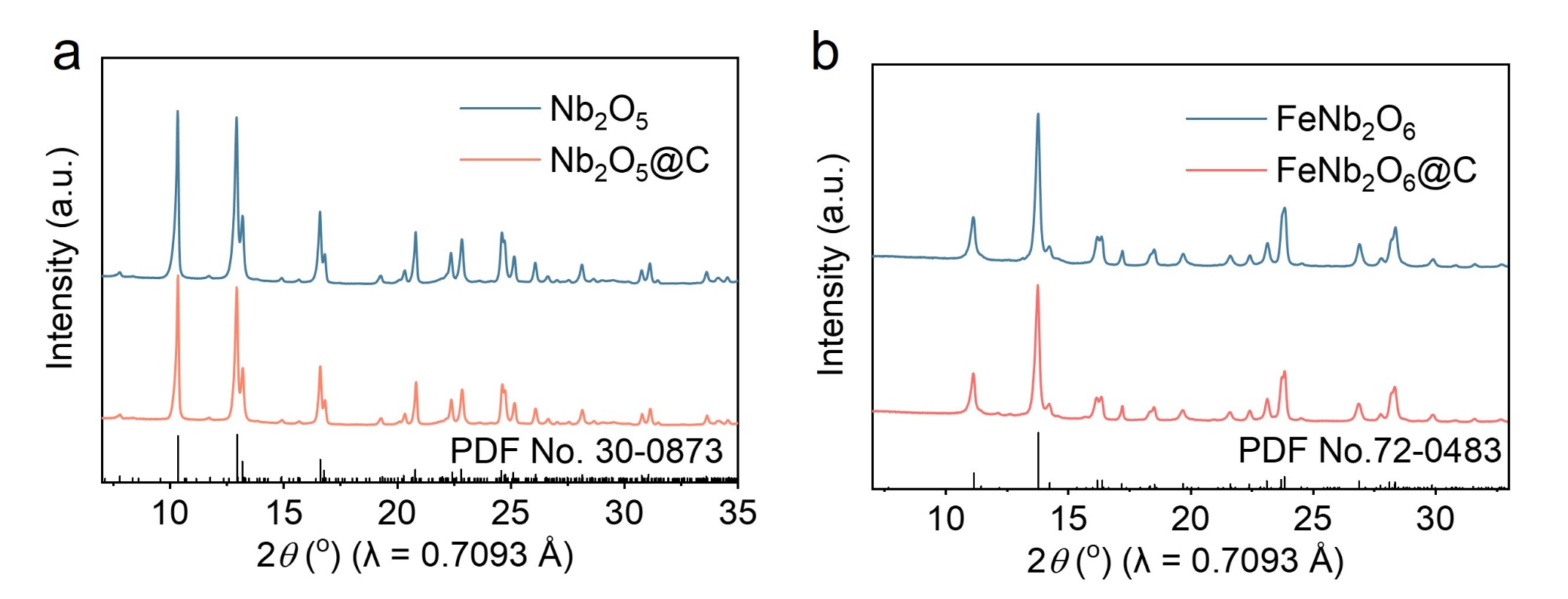


**Figure S1.** (a) XRD patterns of Nb_2_O_5_ and Nb_2_O_5_@C. (b) XRD patterns of FeNb_2_O_6_ and FeNb_2_O_6_@C.

**
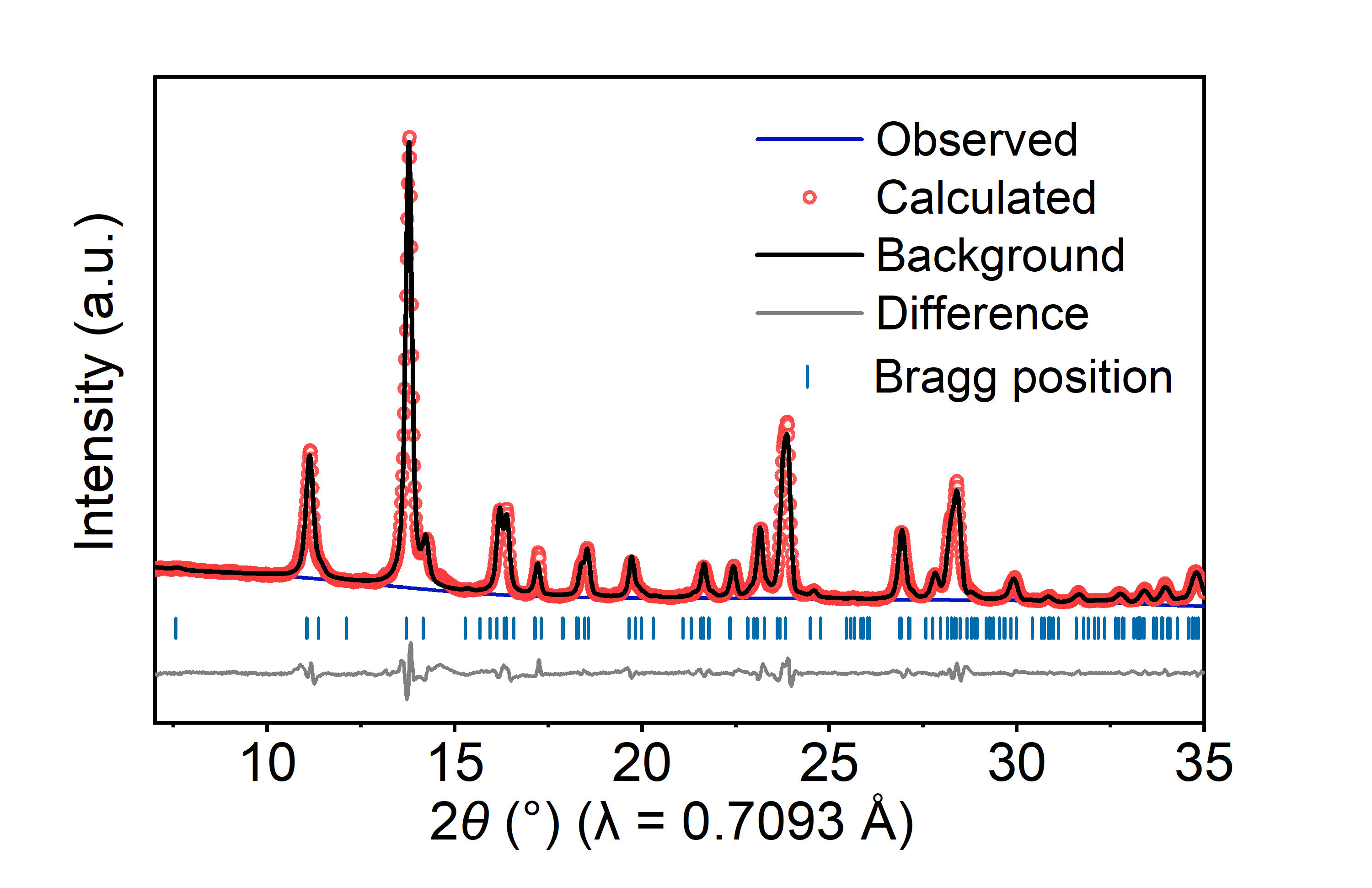
**

**Figure S2.** Rietveld refinement of the XRD pattern of FeNb_2_O_6_.


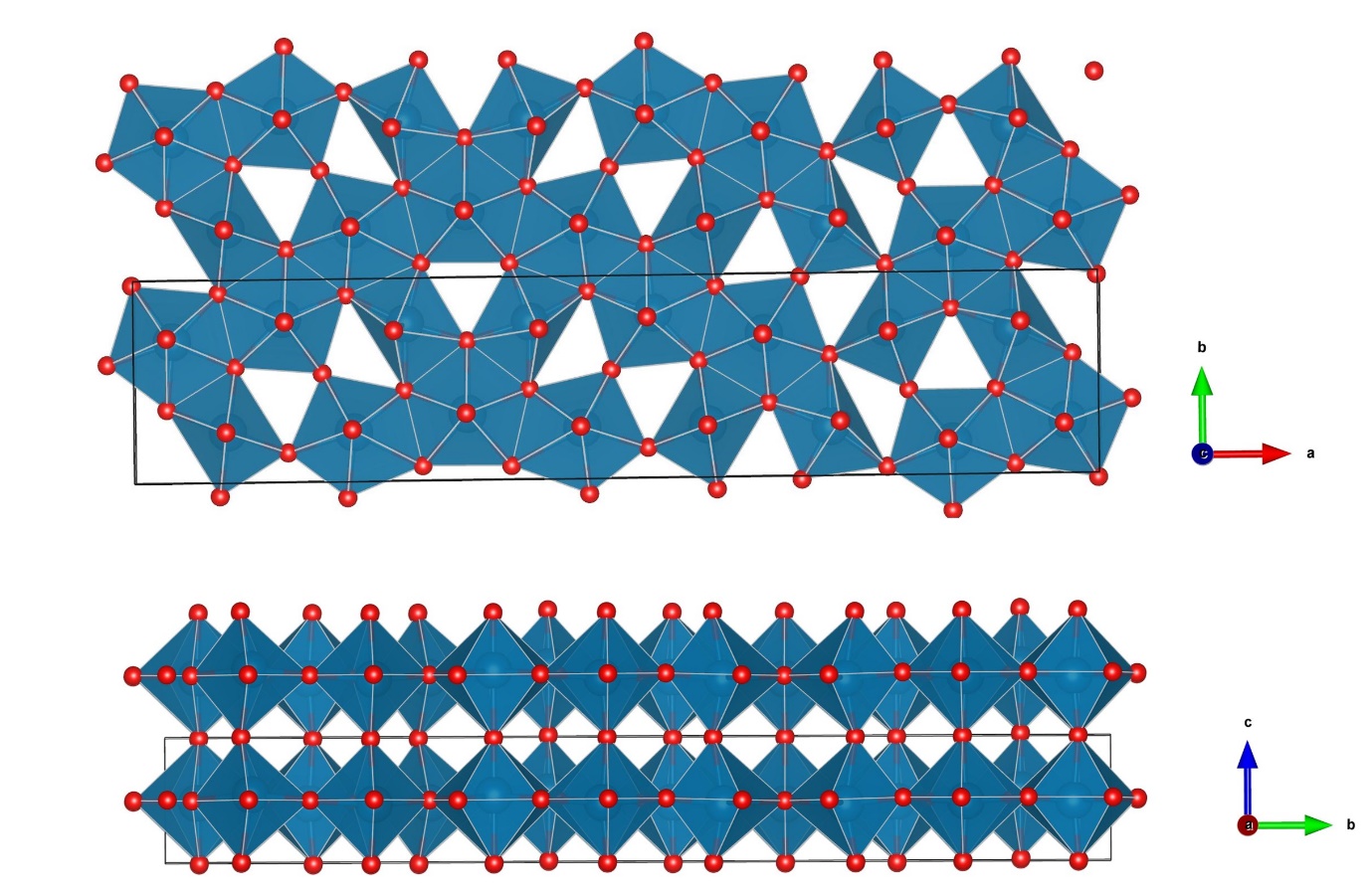


**Figure S3.** Schematic illustration of the structure of *T*-Nb_2_O_5_.


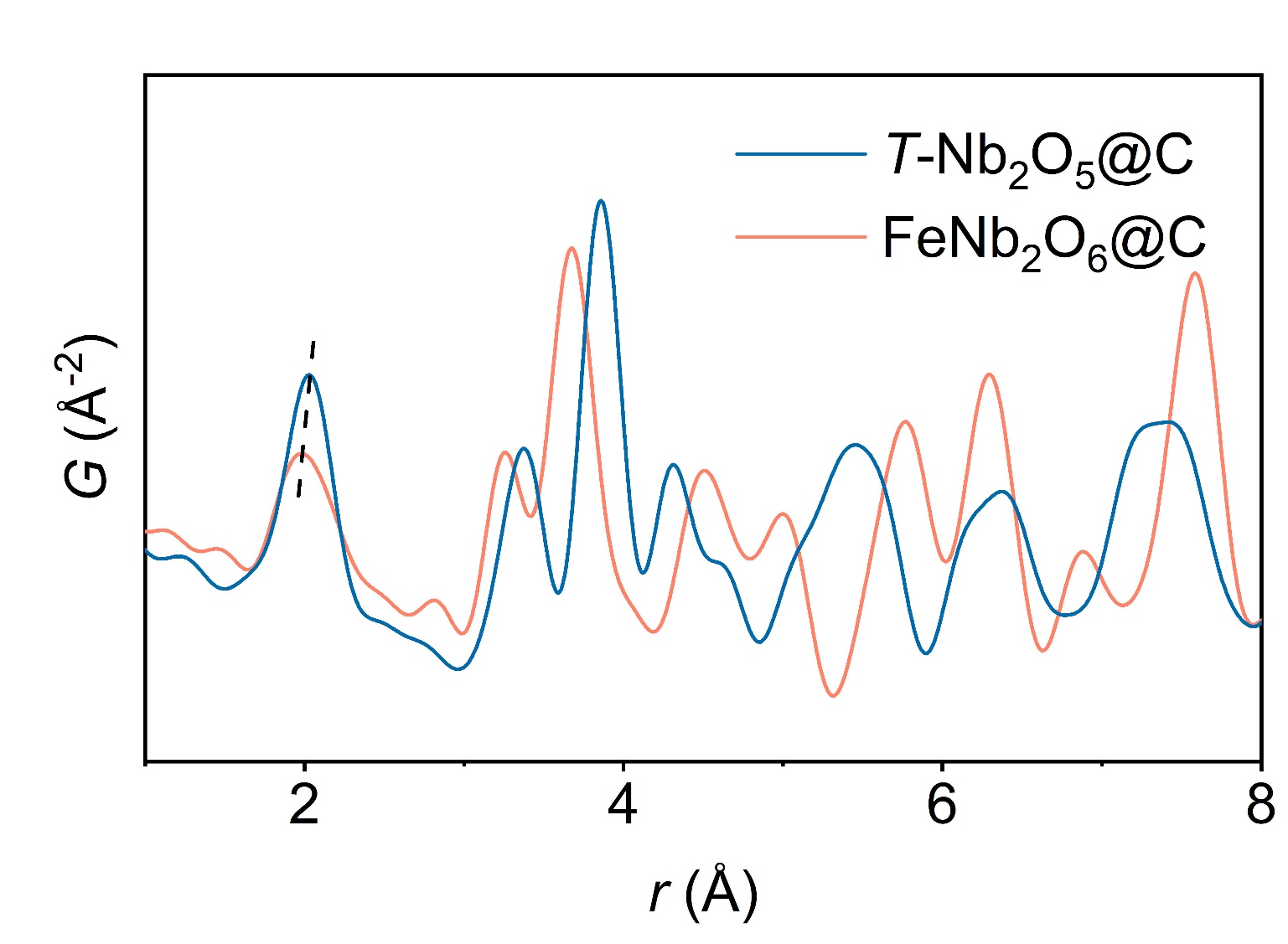


**Figure S4.** PDF patterns of *T*-Nb_2_O_5_@C and FeNb_2_O_6_@C.


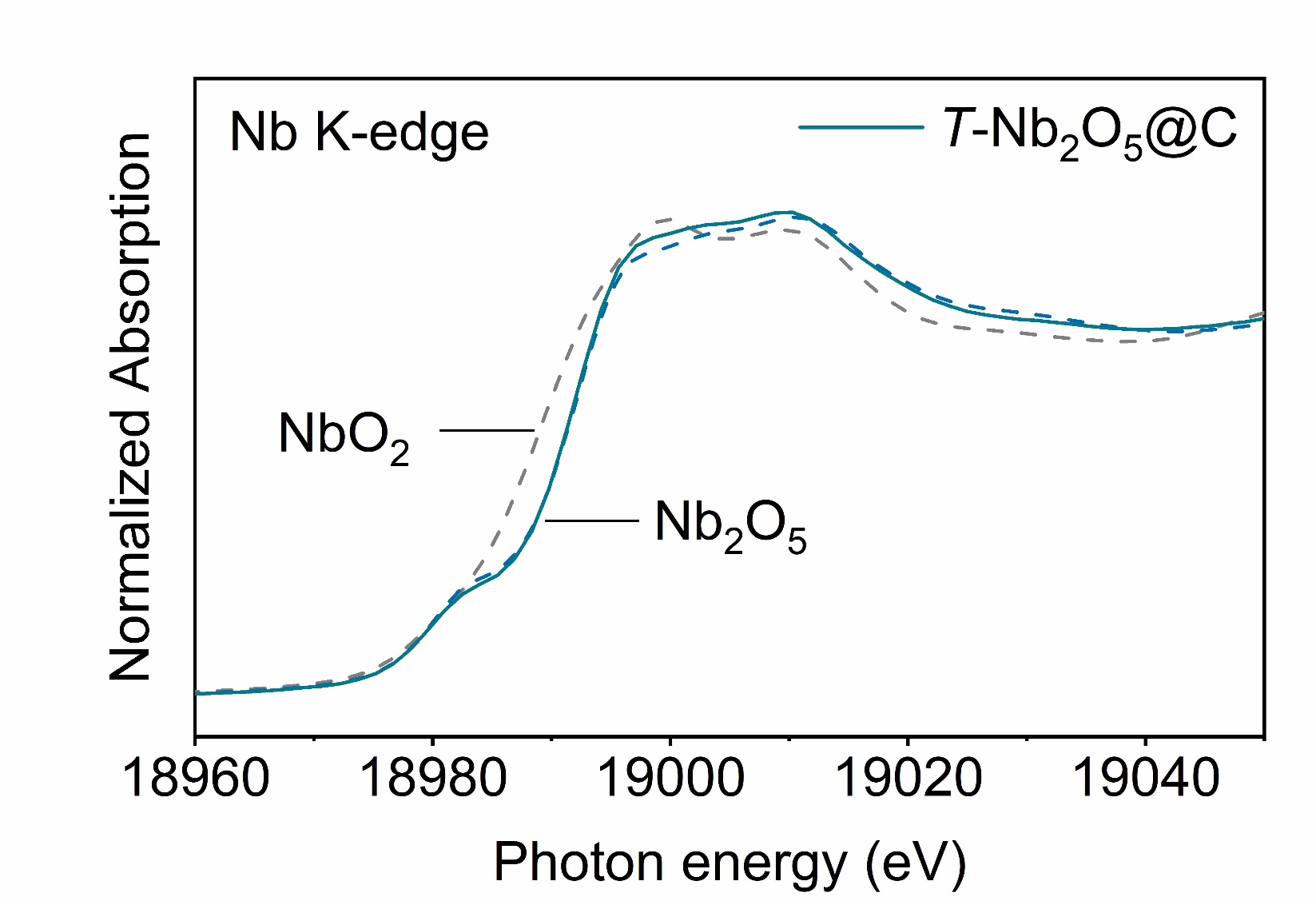


**Figure S5.** Nb K-edge XANES spectrum of *T*-Nb_2_O_5_@C.
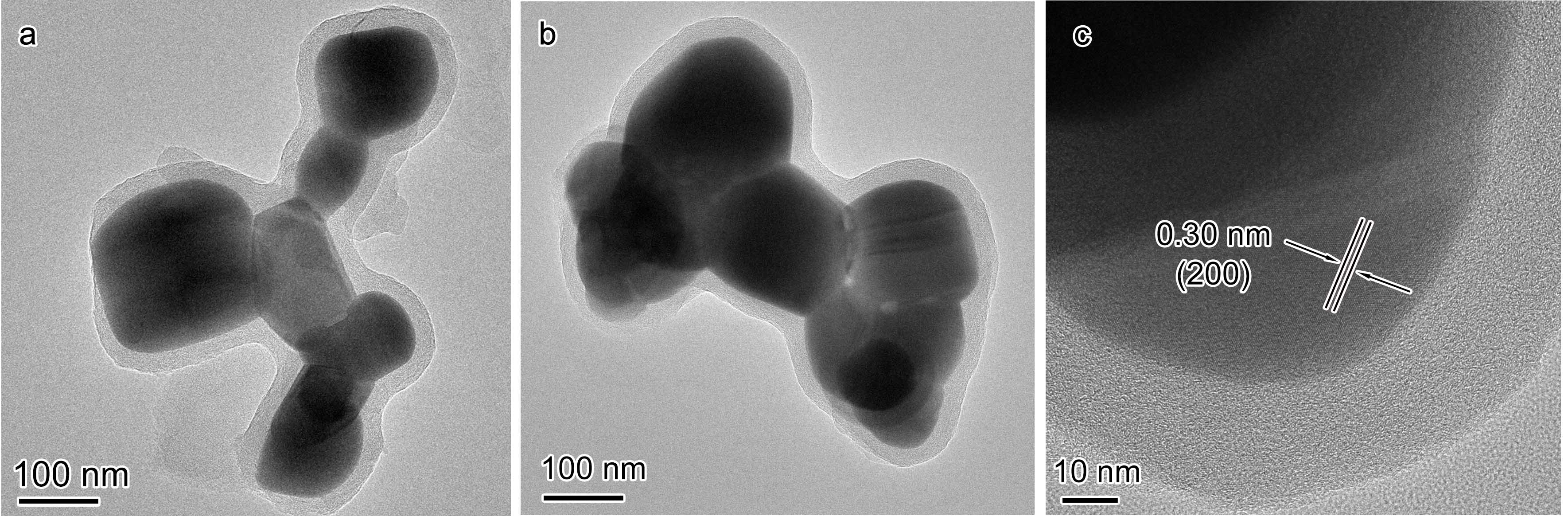


**Figure S6.** (a, b) TEM images of *T*-Nb_2_O_5_@C. (c) HR-TEM image of *T*-Nb_2_O_5_@C.


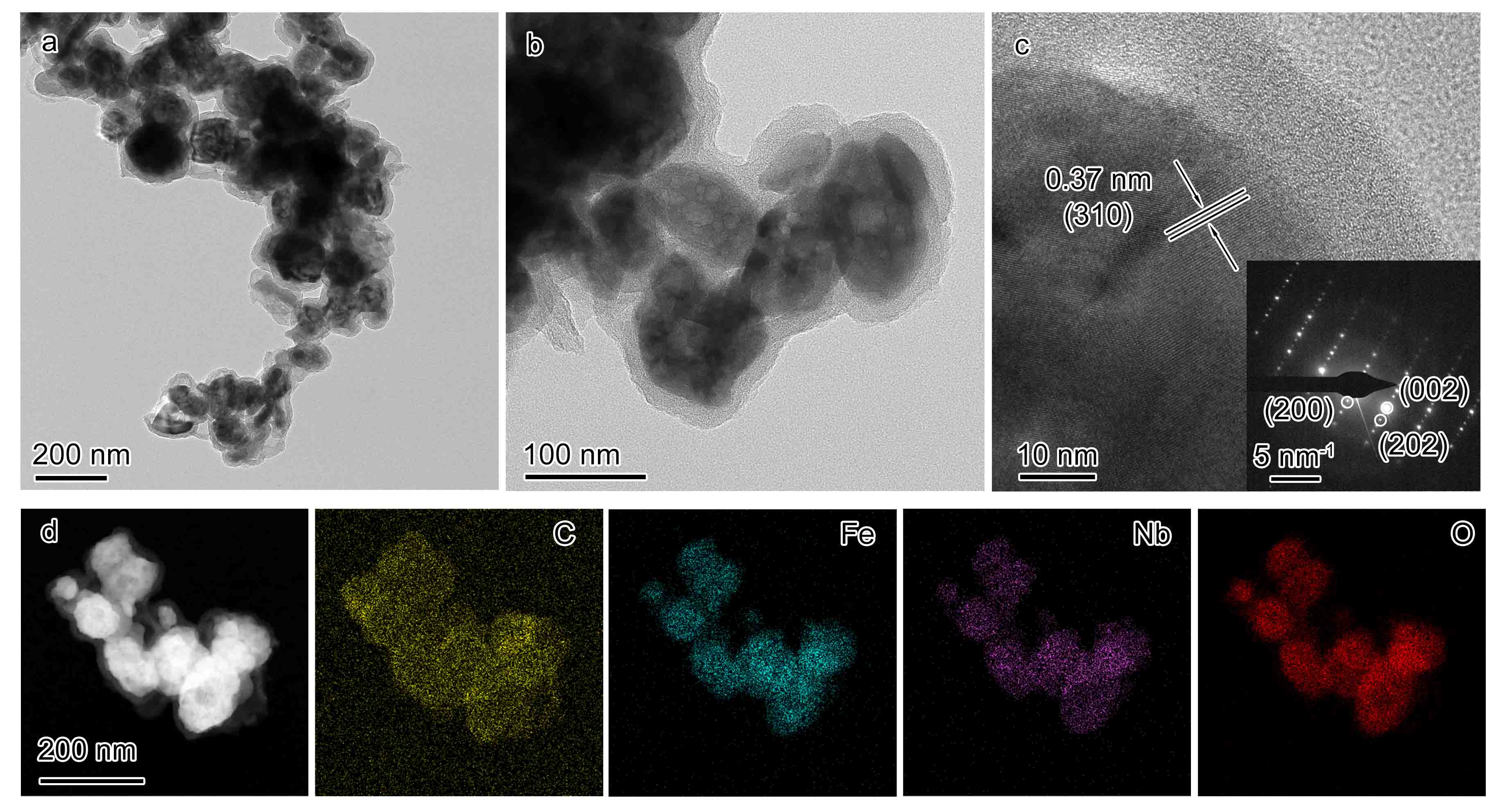


**Figure S7.** (a, b) TEM images of FeNb_2_O_6_@C. (c) HR-TEM and (d) EDS elemental maps of FeNb_2_O_6_@C.


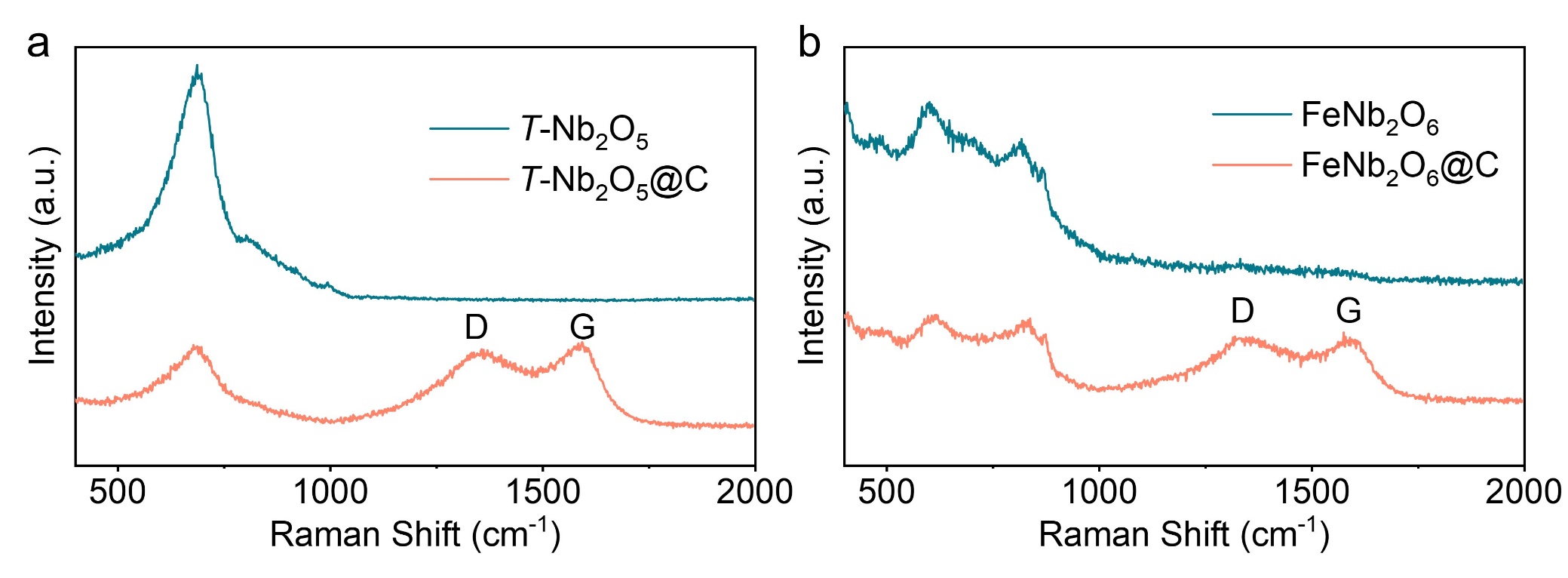


**Figure S8.** Raman spectra of (a)*T*-Nb_2_O_5_ and Nb_2_O_5_@C and (b) FeNb_2_O_6_ and FeNb_2_O_6_@C.


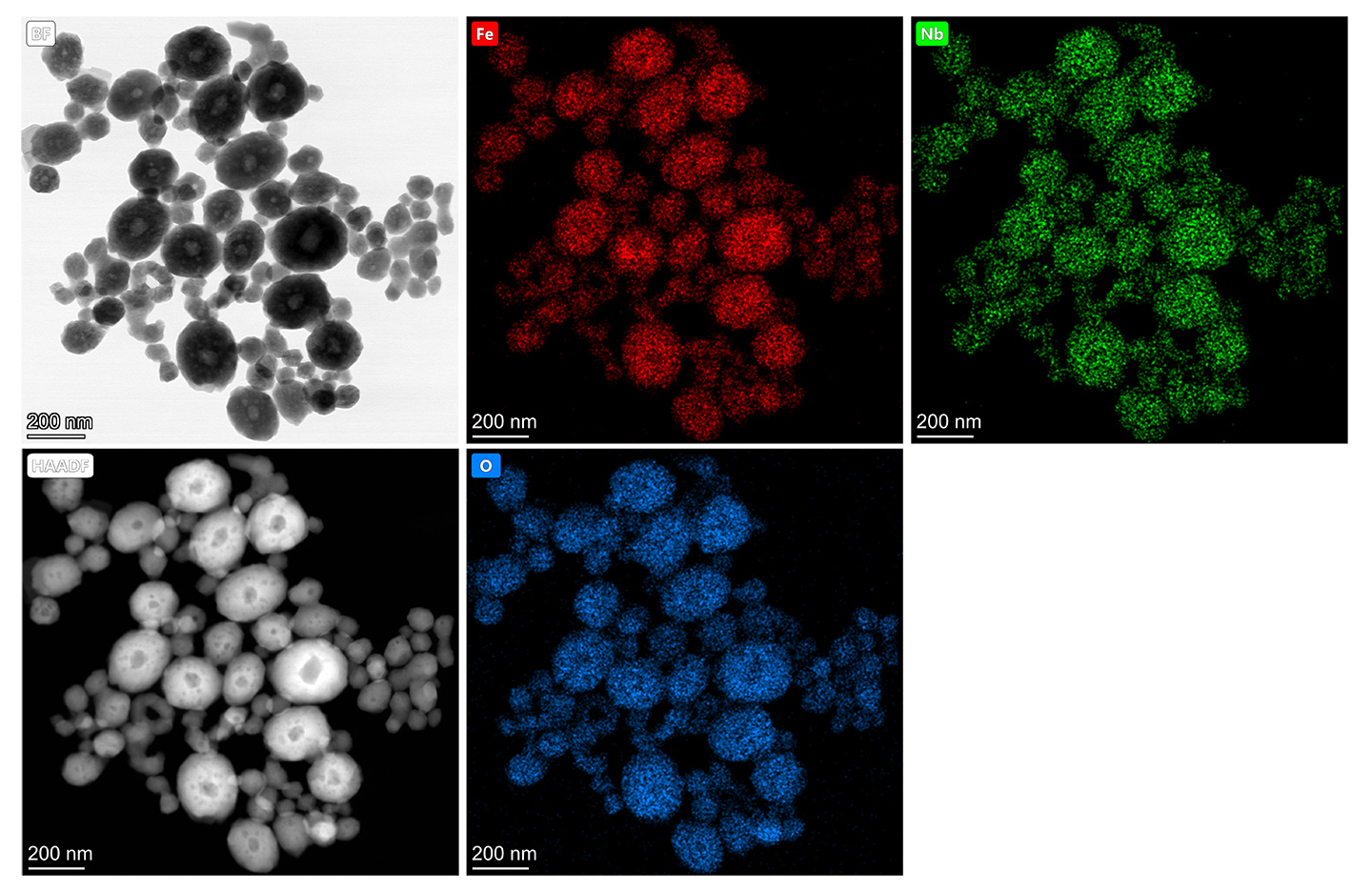


**Figure S9**. EDS elemental maps of FeNb_2_O_6_.

**
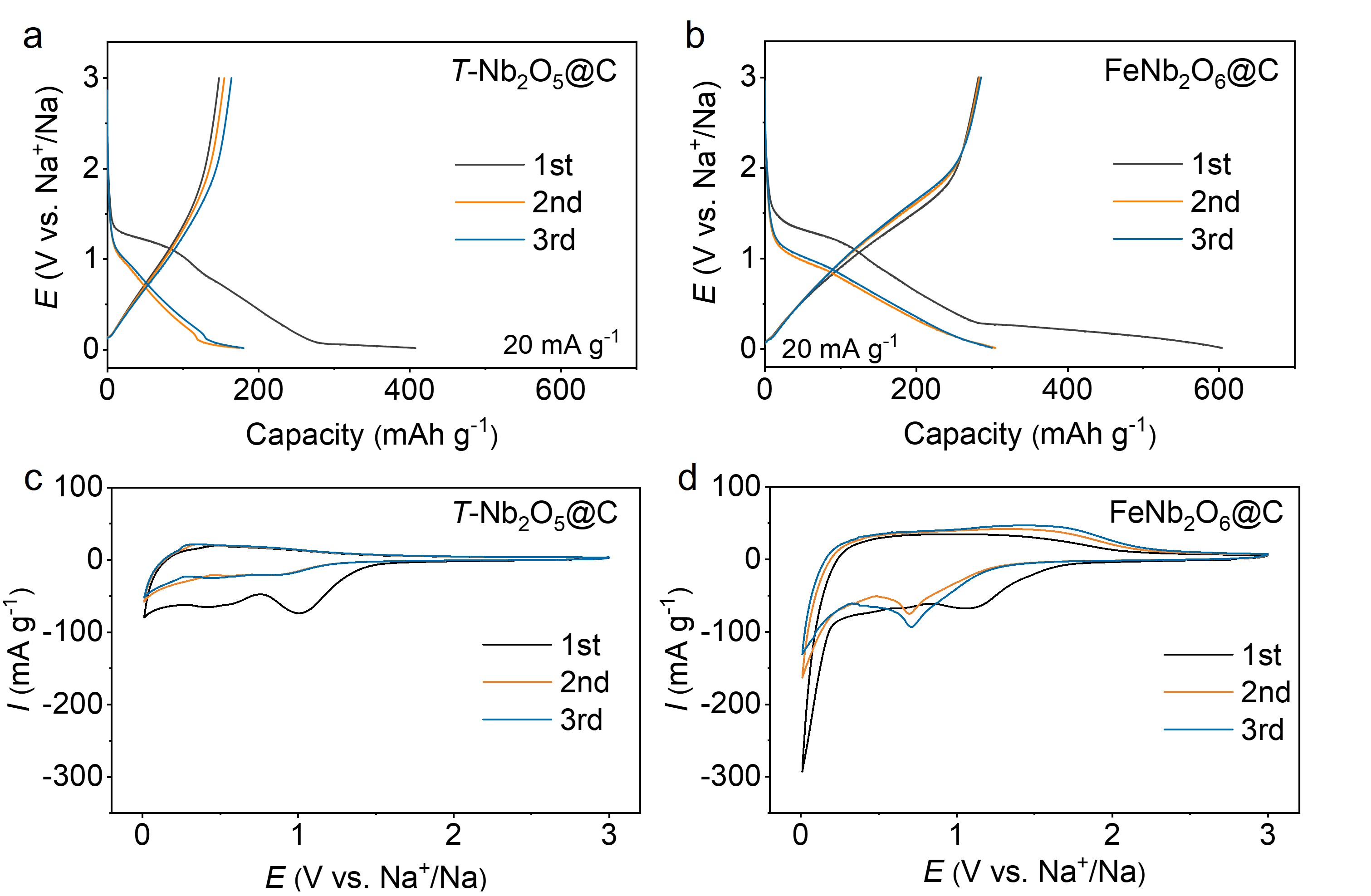
**

**Figure S10.** Discharge and charge voltage profiles of the initial three cycles for (a) *T*-Nb_2_O_5_@C and (b) FeNb_2_O_6_@C. CV curves of (c) *T*-Nb_2_O_5_@C and (d) FeNb_2_O_6_@C at a scan rate of 0.1 mV s^-1^.


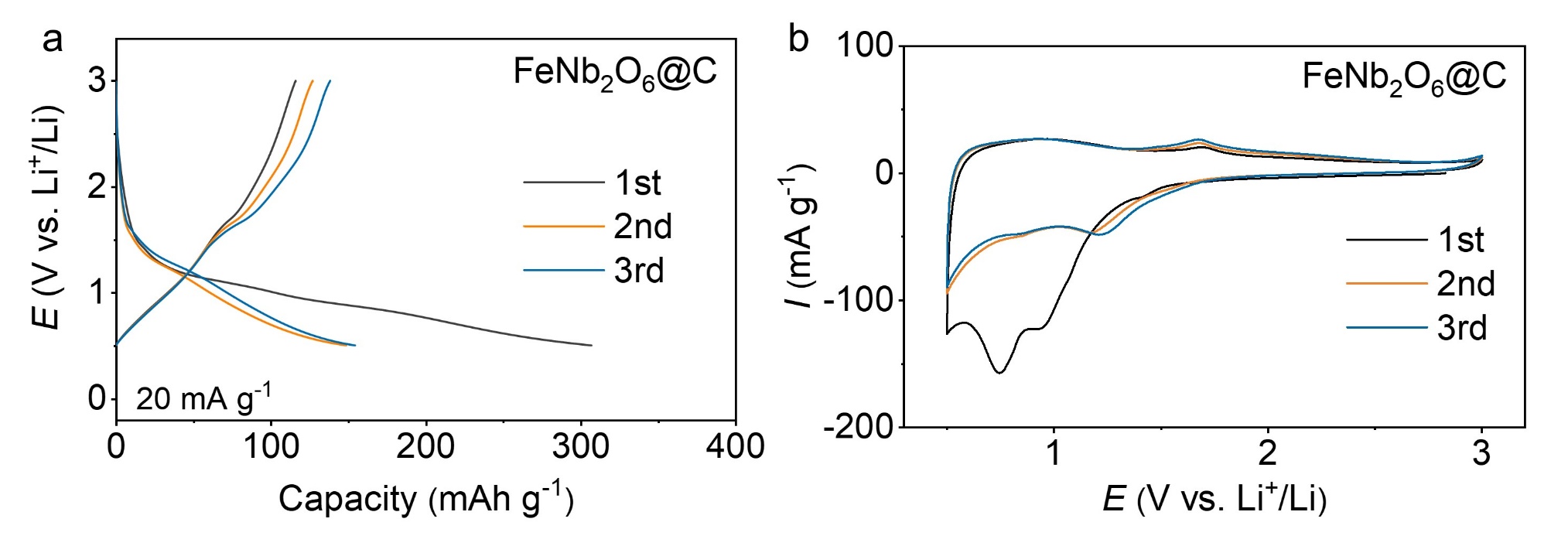


**Figure S11**. Discharge and charge voltage profiles of the initial three Li-ion insertion/extraction cycles for FeNb_2_O_6_@C. (b) CV curves of FeNb_2_O_6_@C at a scan rate of 0.1 mV s^-1^.


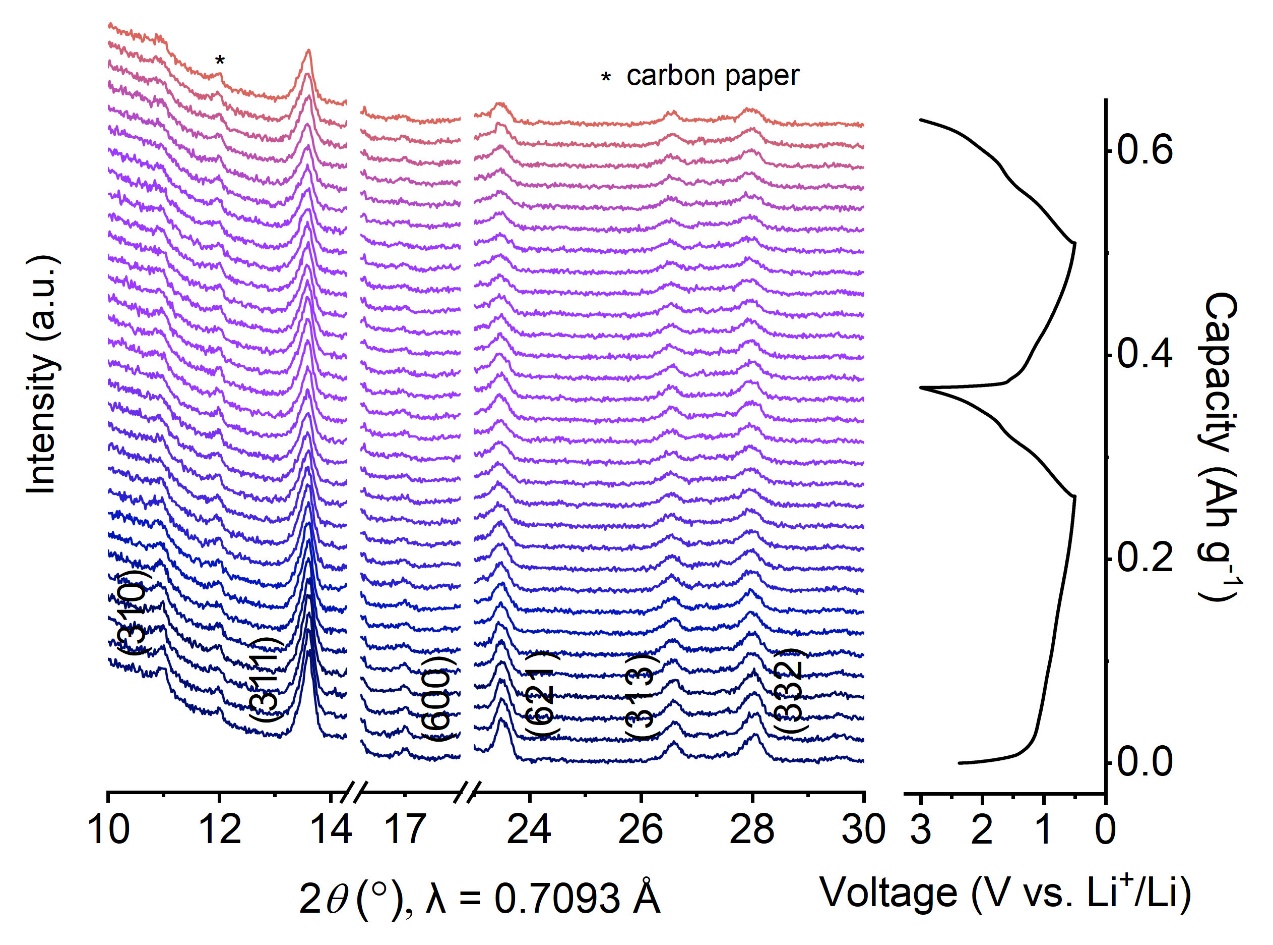


**Figure S12**. *Operando* XRD patterns of FeNb_2_O_6_@C in half-cell with Li foil anode collected for the first and second cycles (the corresponding voltage profiles are shown on the right side).


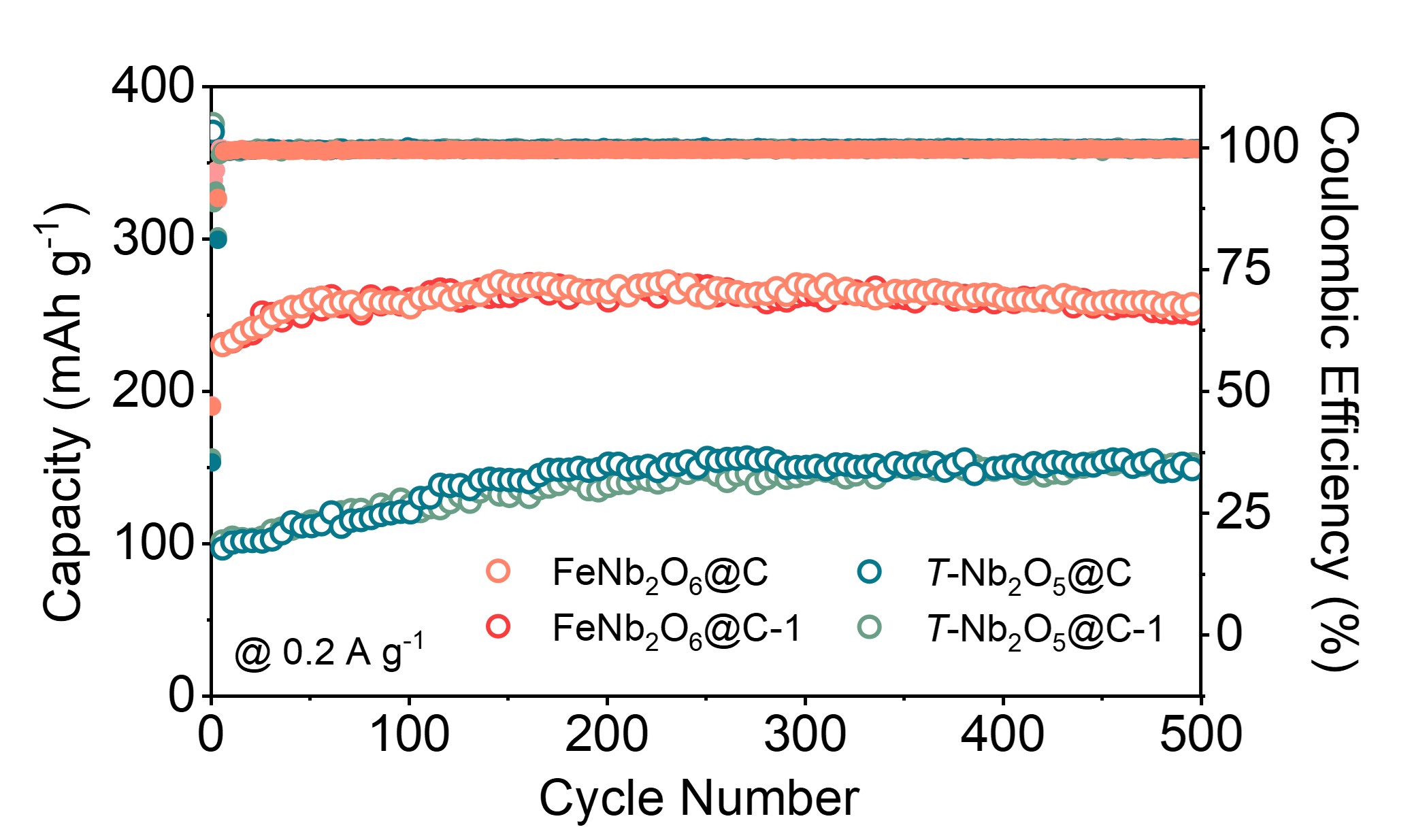


**Figure S13**. Long cycling stability of *T*-Nb_2_O_5_@C and FeNb_2_O_6_@C at 0.2 A g^-1^.


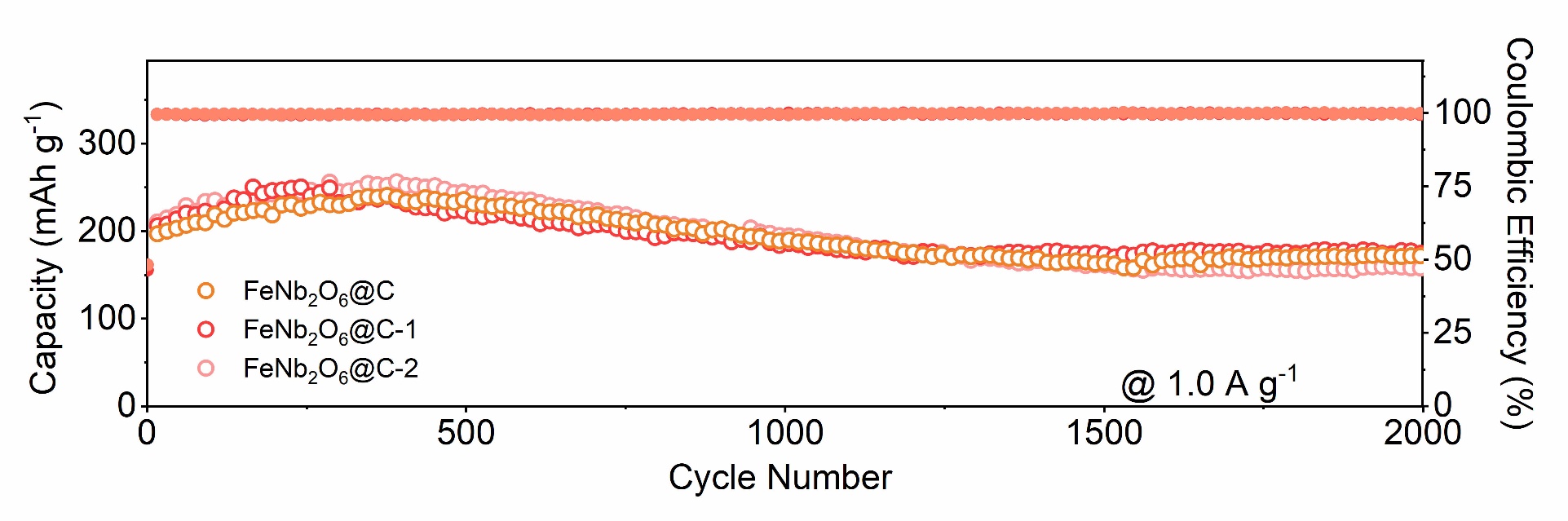


**Figure S14**. Long cycling stability measured for three independent half-cells of FeNb_2_O_6_@C at 1.0 A g^-1^.


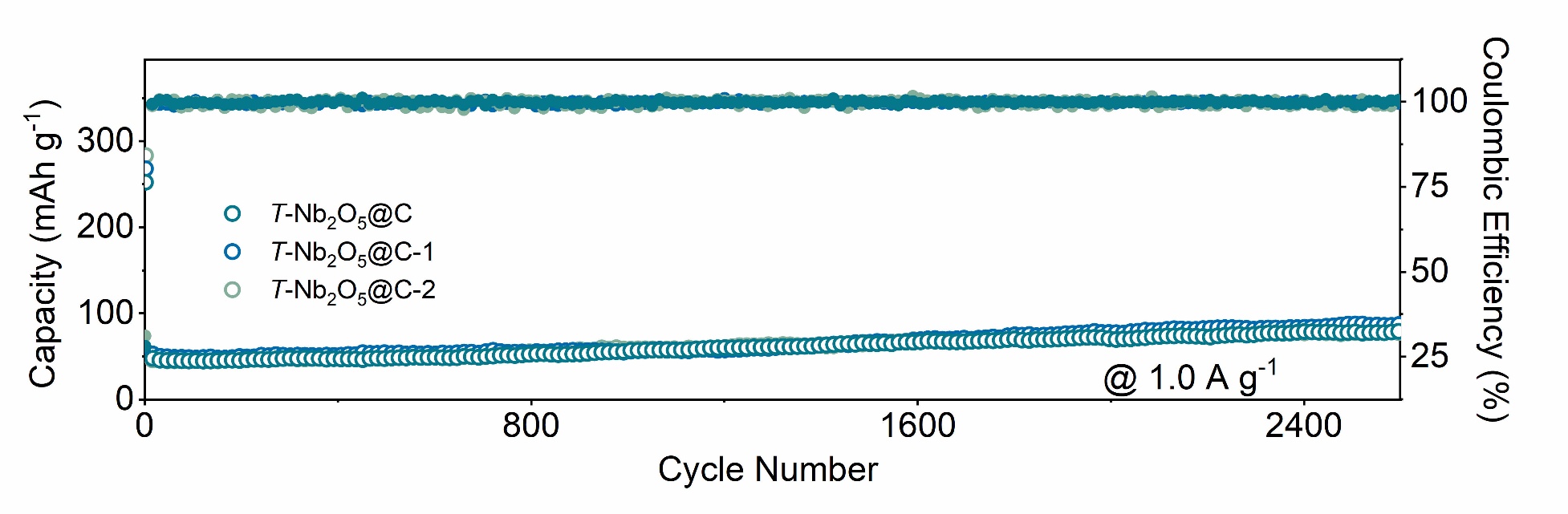


**Figure S15.** Long cycling stability measured for three independent half-cells of *T*-Nb_2_O_5_@C at 1.0 A g^-1^.


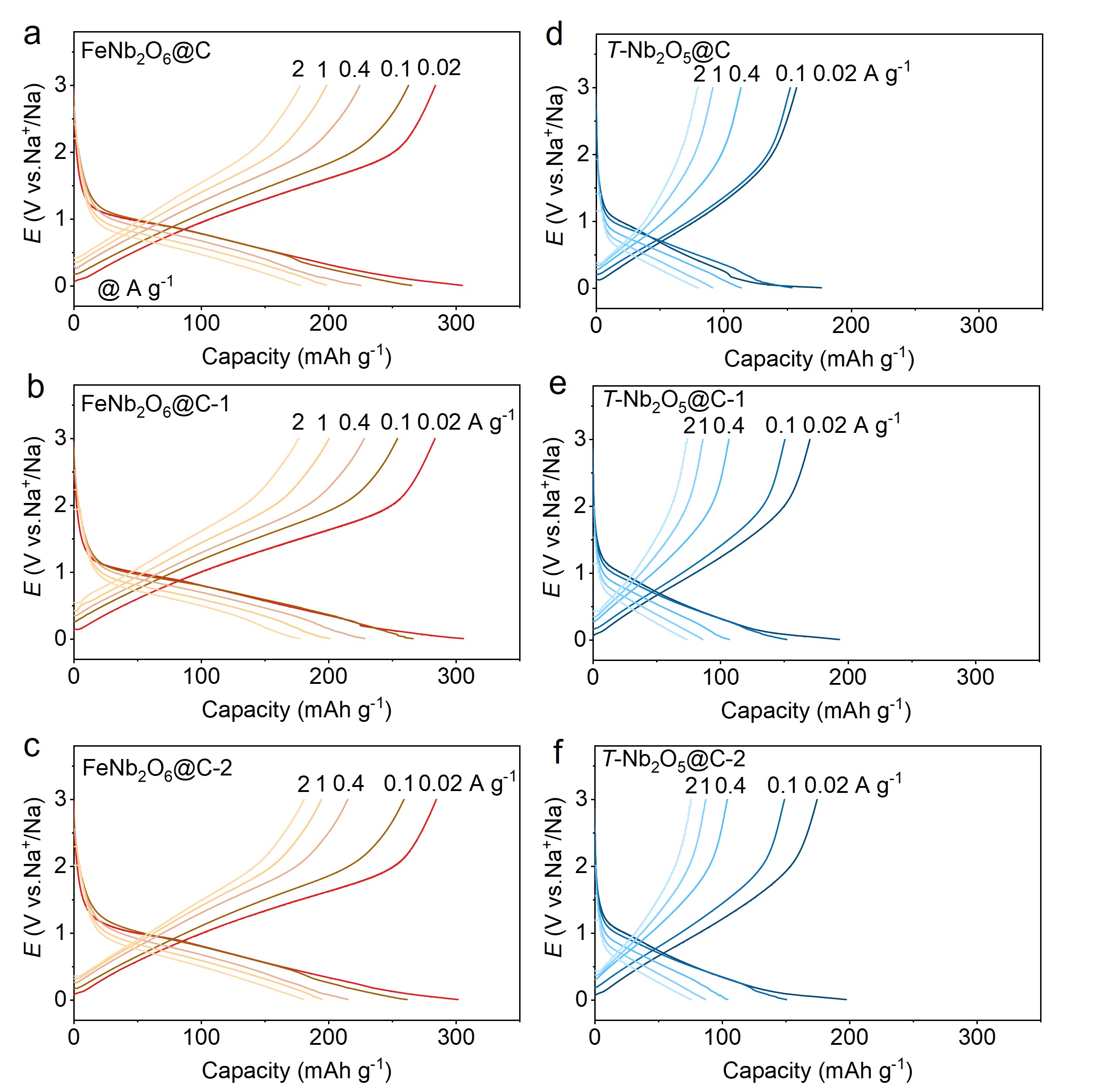


**Figure S16**. Discharge-charge curves measured for three independent half-cells of (a), (b), (c) FeNb_2_O_6_@C and (d), (e), (f) *T*-Nb_2_O_5_@C at different rates.


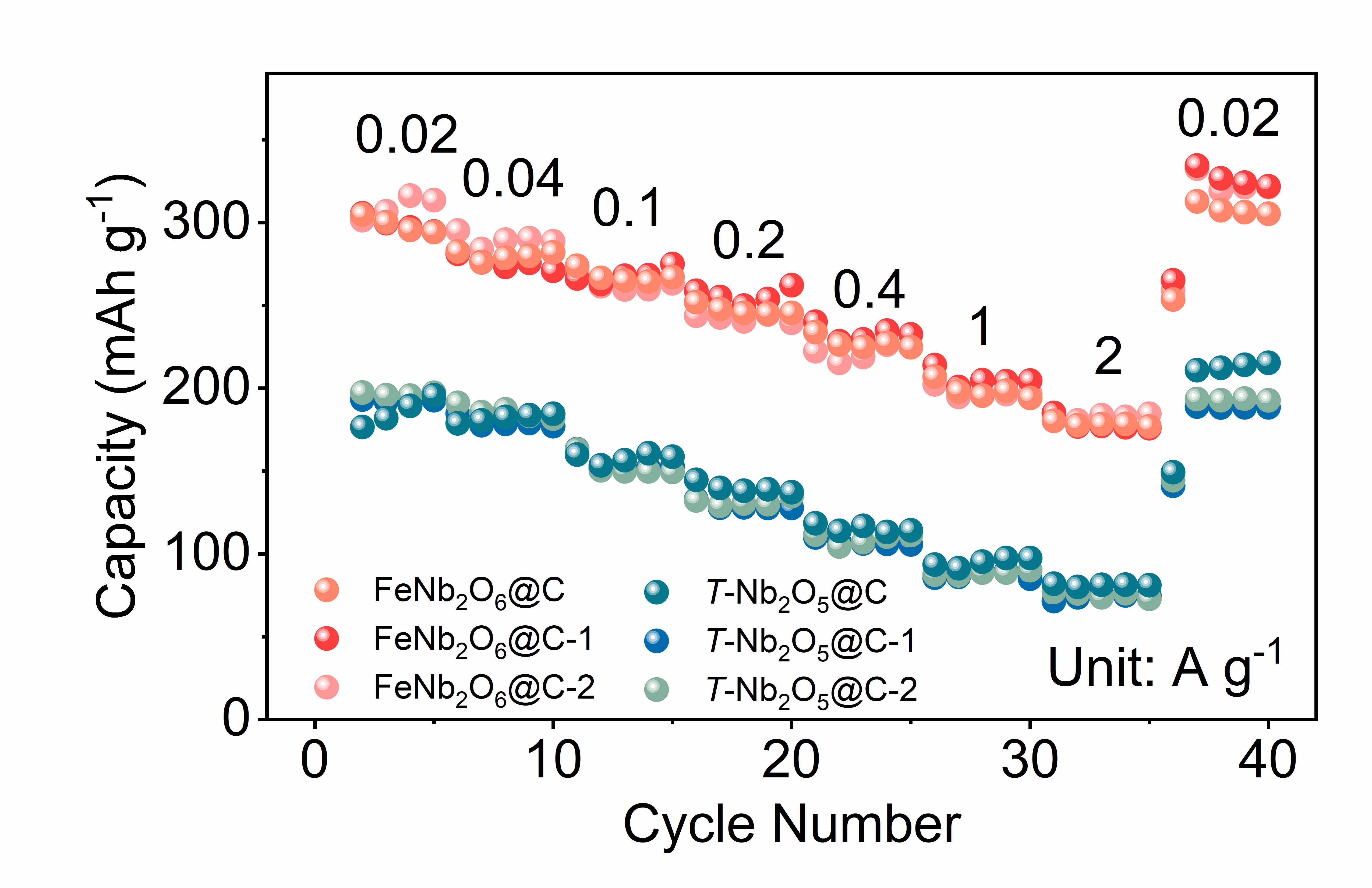


**Figure S17**. Rate capability measured for three independent half-cells of FeNb_2_O_6_@C and *T*-Nb_2_O_5_@C at different rates.

**
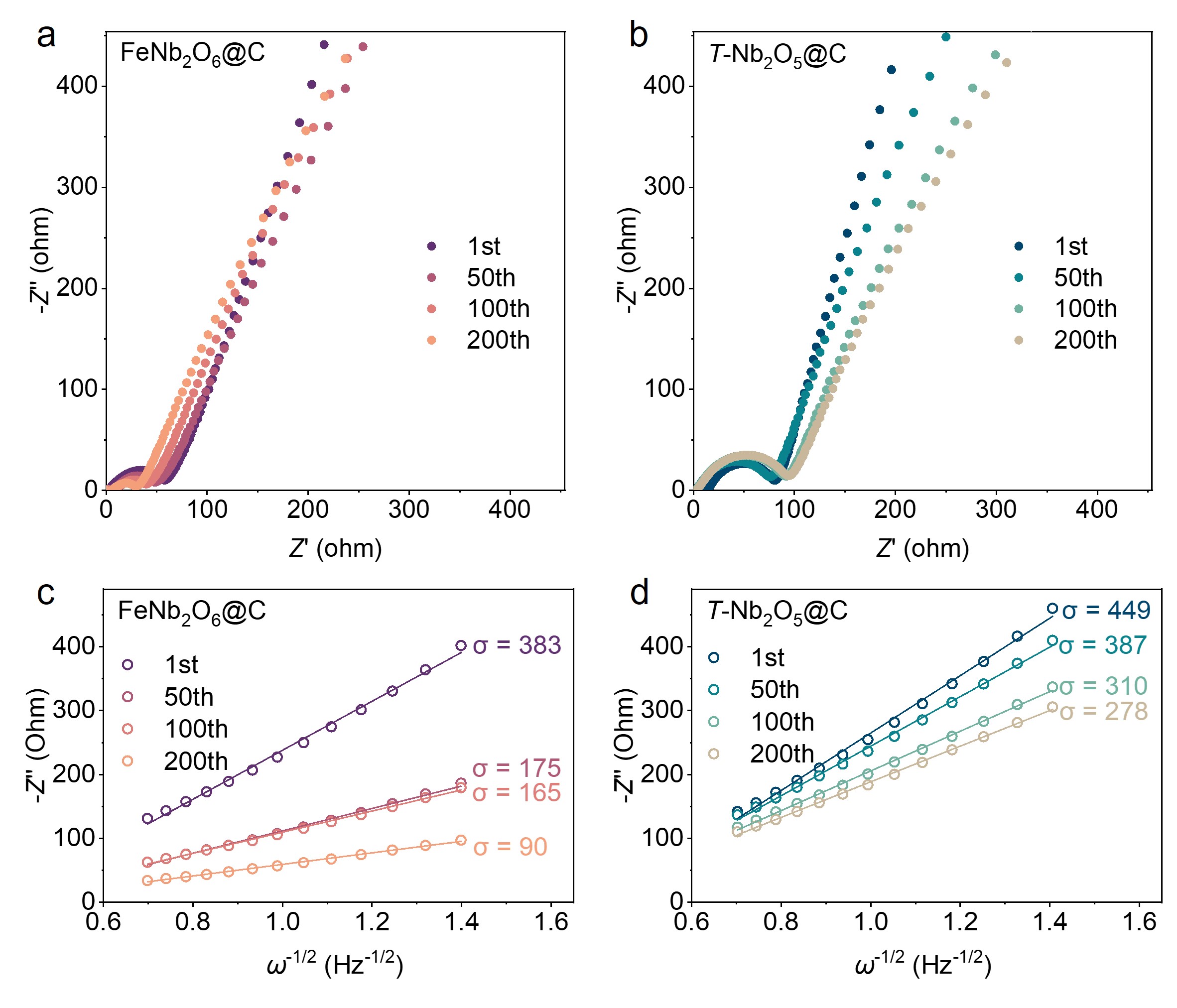
**

**Figure S18.** Nyquist plots of (a) FeNb_2_O_6_@C and (b) *T*-Nb_2_O_5_@C at different cycling stages. Low-frequency part of imaginary impedance (*Z*′′) as a function of *ω*^−1/2^ for (c) FeNb_2_O_6_@C and (d) *T*-Nb_2_O_5_@C. *ω* is the angular frequency, and the slope of the linear fit corresponds to the Warburg coefficient *σ.* The relationship between the Na^+^ diffusion coefficient and Warburg coefficient *σ* is given by the equation $\text{D}\text{= }\frac{\text{R}^{\text{2}}\text{T}^{\text{2}}}{\text{2}\text{n}^{\text{4}}\text{F}^{\text{4}}\text{A}^{\text{2}}\text{C}^{\text{2}}\text{σ}^{\text{2}}}$, where *R* is the gas constant, *T* is the absolute temperature, *A* is the area of the electrode, *n* is the number of electrons per reaction species, *F* is the Faraday constant, and *C* is the concentration of Na^+^.


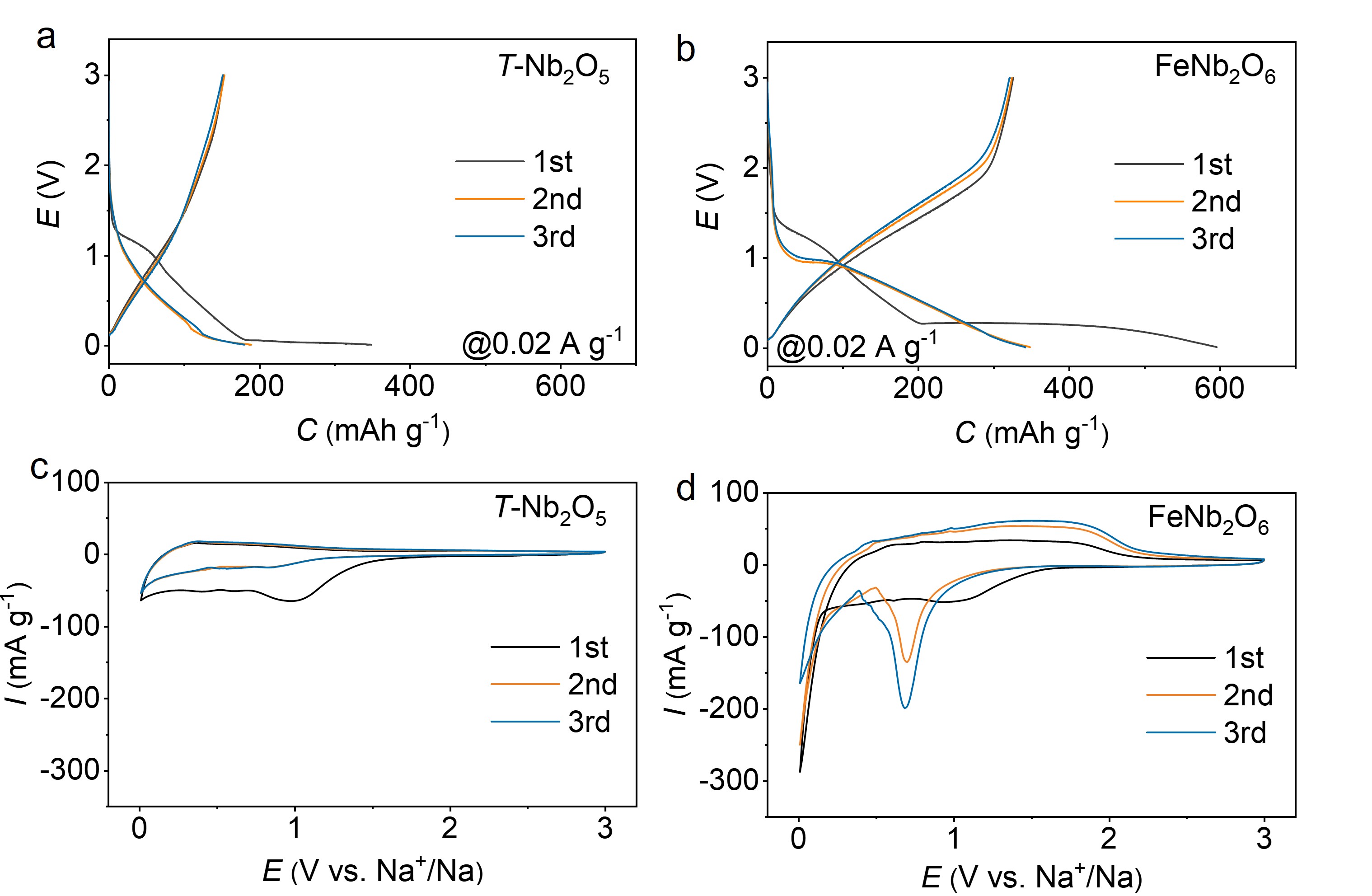


**Figure S19.** Electrochemical performance of *T*-Nb_2_O_5_ and FeNb_2_O_6_. Discharge and charge voltage profiles of the initial three cycles for (a) *T*-Nb_2_O_5_ and (b) FeNb_2_O_6_. CV curves of (c) *T*-Nb_2_O_5_ and (d) FeNb_2_O_6_ at a scan rate of 0.1 mV s^-1^.


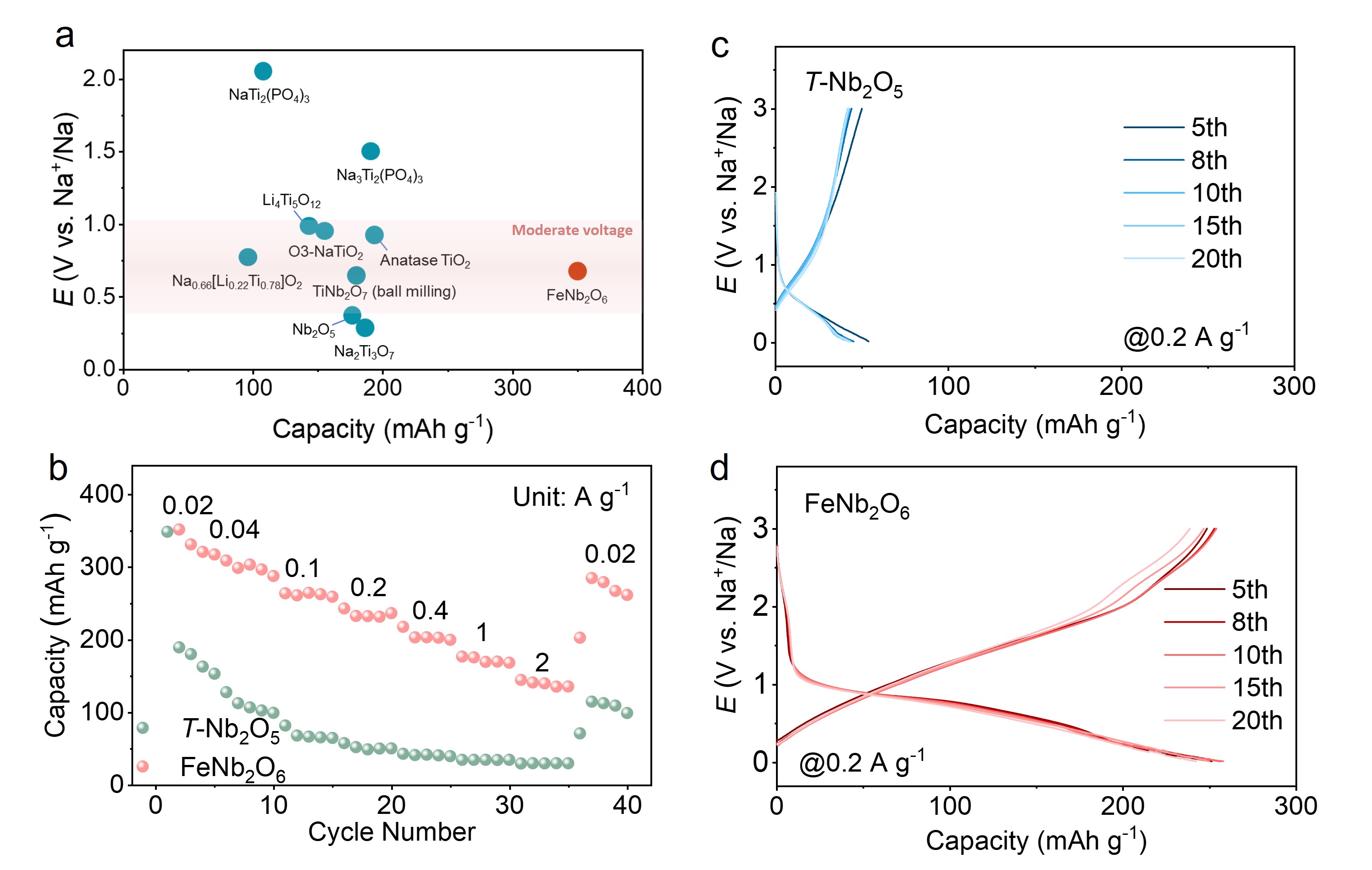


**Figure S20.** (a) Comparison of the electrochemical results obtained for FeNb_2_O_6_ in this work with those of previously reported Ti-based and Nb-based anodes^17-24^. (b) Rate capability of *T*-Nb_2_O_5_ and FeNb_2_O_6_. Discharge and charge voltage profiles of extended cycles of (c) *T*-Nb_2_O_5_ and (d) FeNb_2_O_6_ at 0.2 A g^-1^.


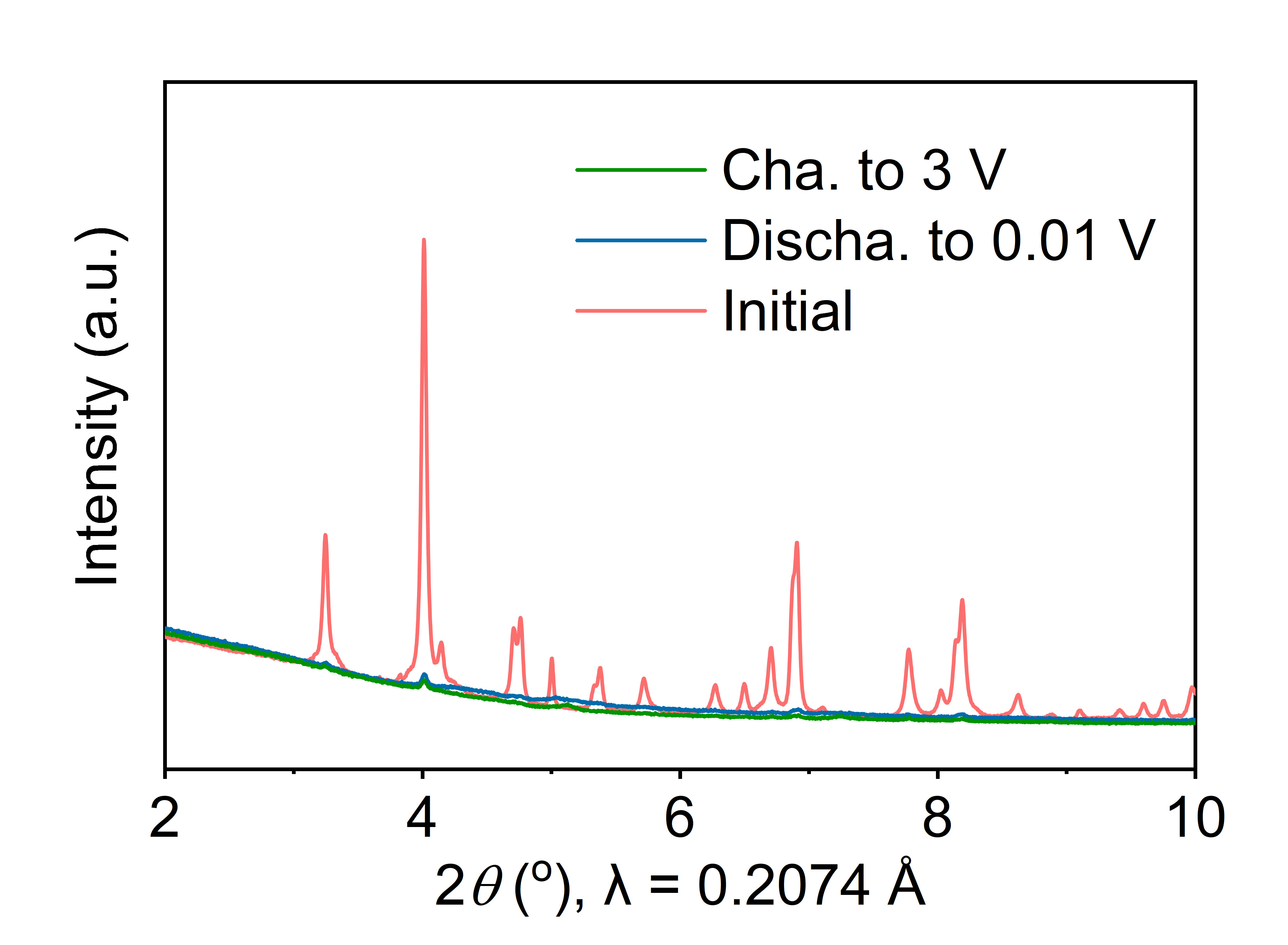


**Figure S21.** Ex situ synchrotron XRD patterns of FeNb_2_O_6_@C.


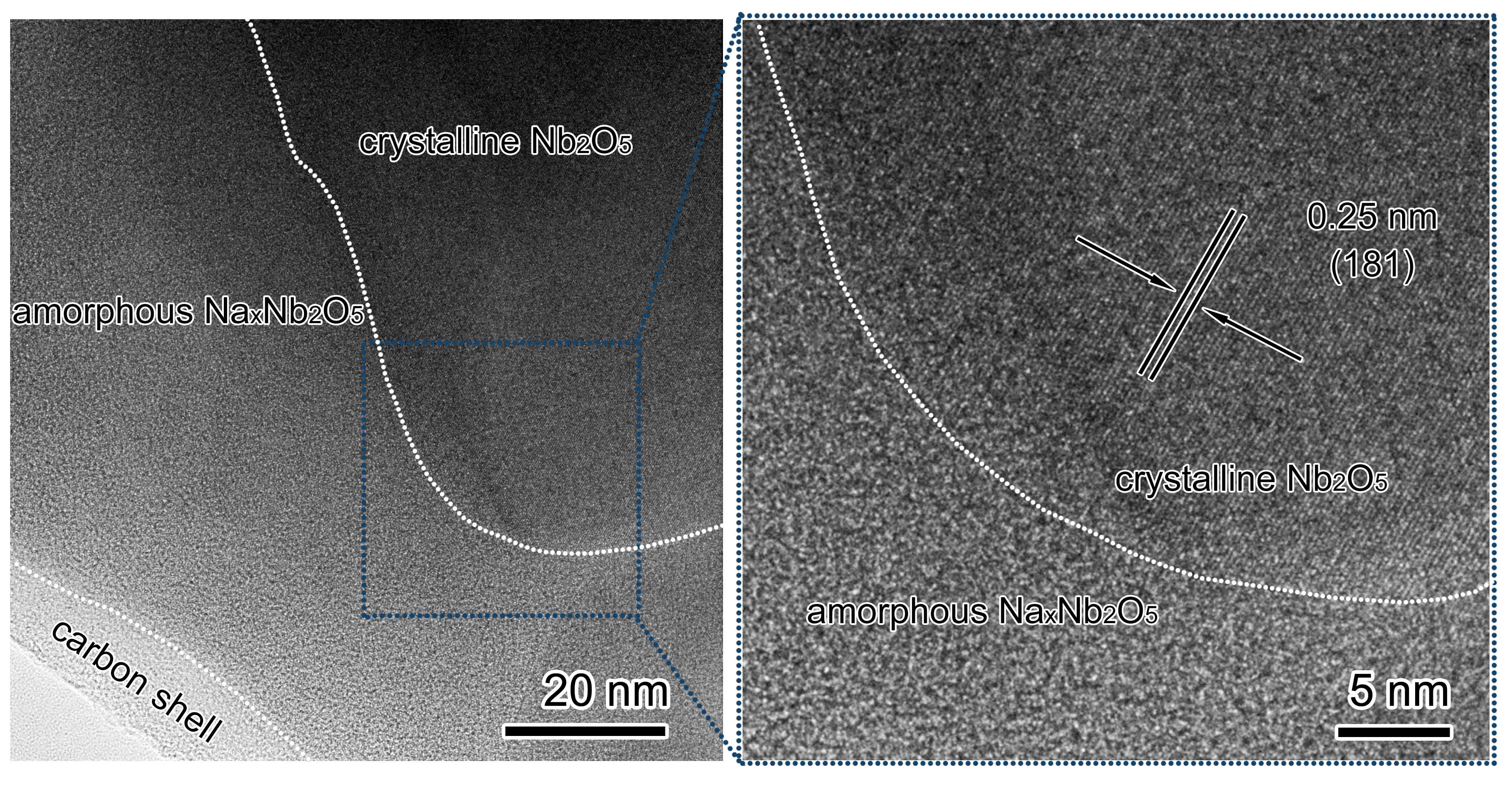


**Figure S22.** Ex situ HR-TEM images of *T*-Nb_2_O_5_@C at the fully sodiated state, showing an amorphous Na_x_Nb_2_O_5_ surface region and crystalline Nb_2_O_5_ core.


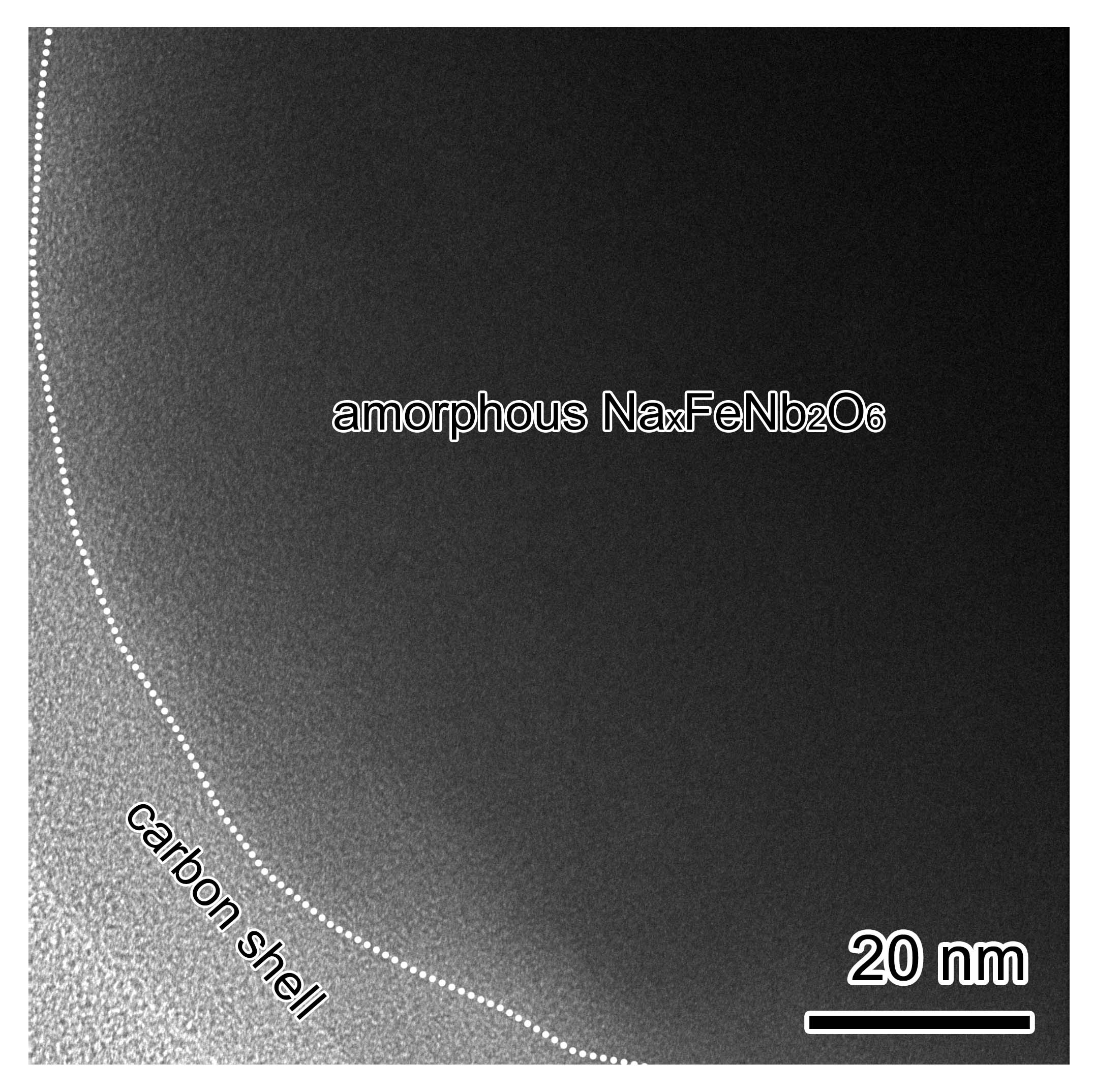


**Figure S23.** Ex situ HR-TEM image of FeNb_2_O_6_@C at the fully sodiated state, showing the amorphous Na_x_FeNb_2_O_6_.


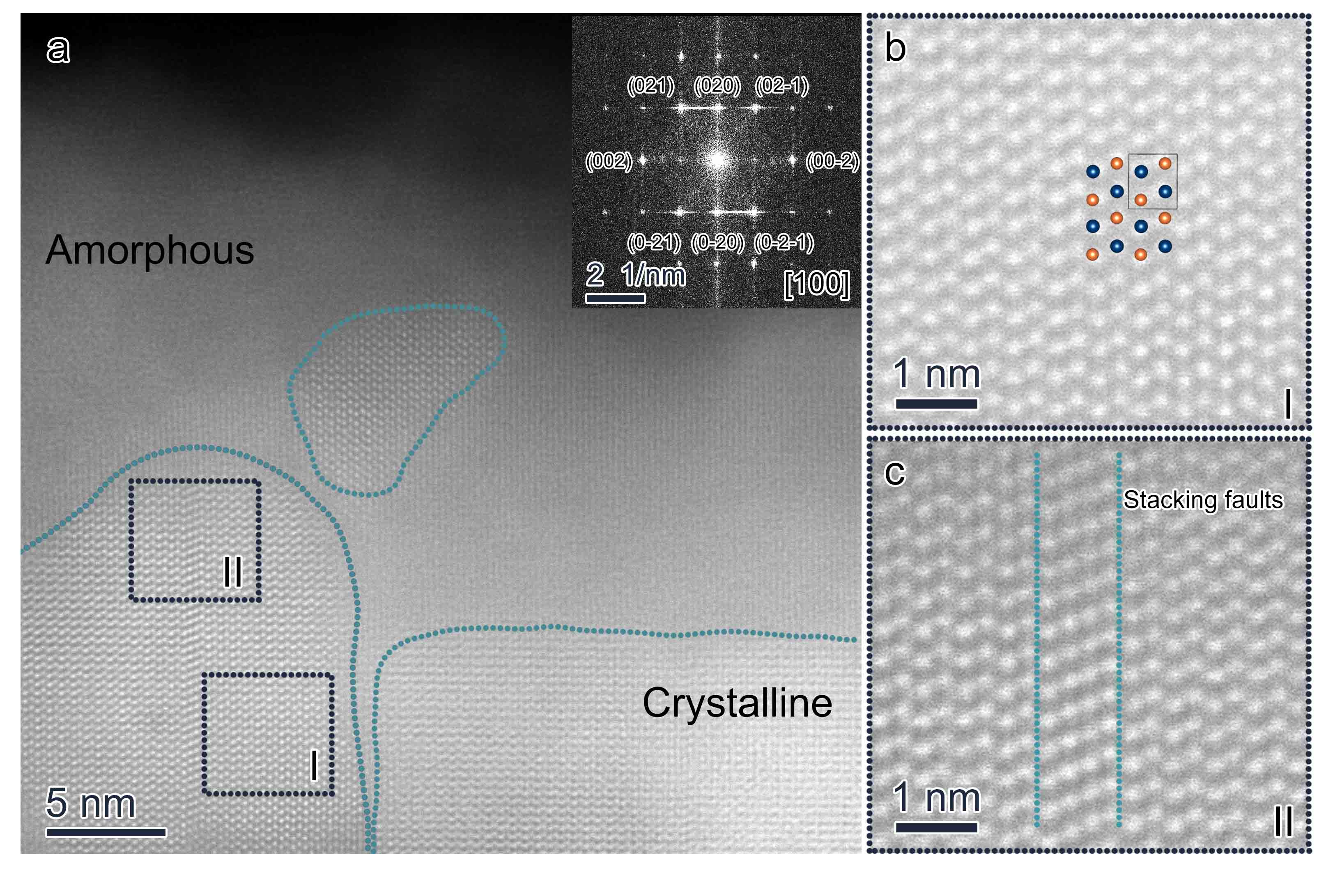


**Figure S24**. (a) AC-STEM image of FeNb_2_O_6_ after discharge to 0.1 V; FFT pattern of the partial crystalline region is shown in the inset. (b) and (c) Magnified AC-STEM image of the rectangular regions Ⅰ and Ⅱ delimited in (a) (Fe and Nb atoms are indicated by orange and blue spheres, respectively).


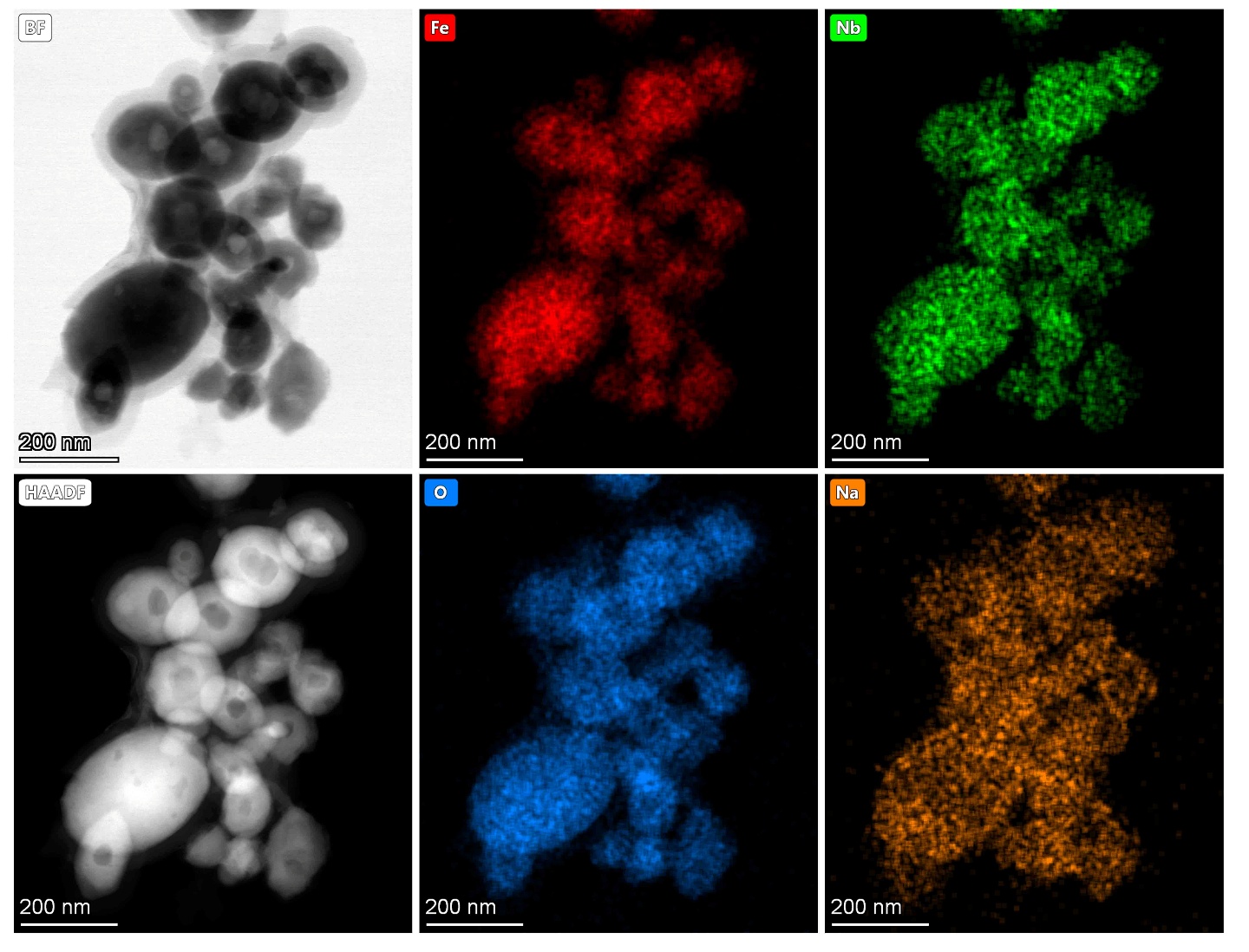


**Figure S25**. EDS elemental maps of FeNb_2_O_6_ after discharge to 0.1 V.


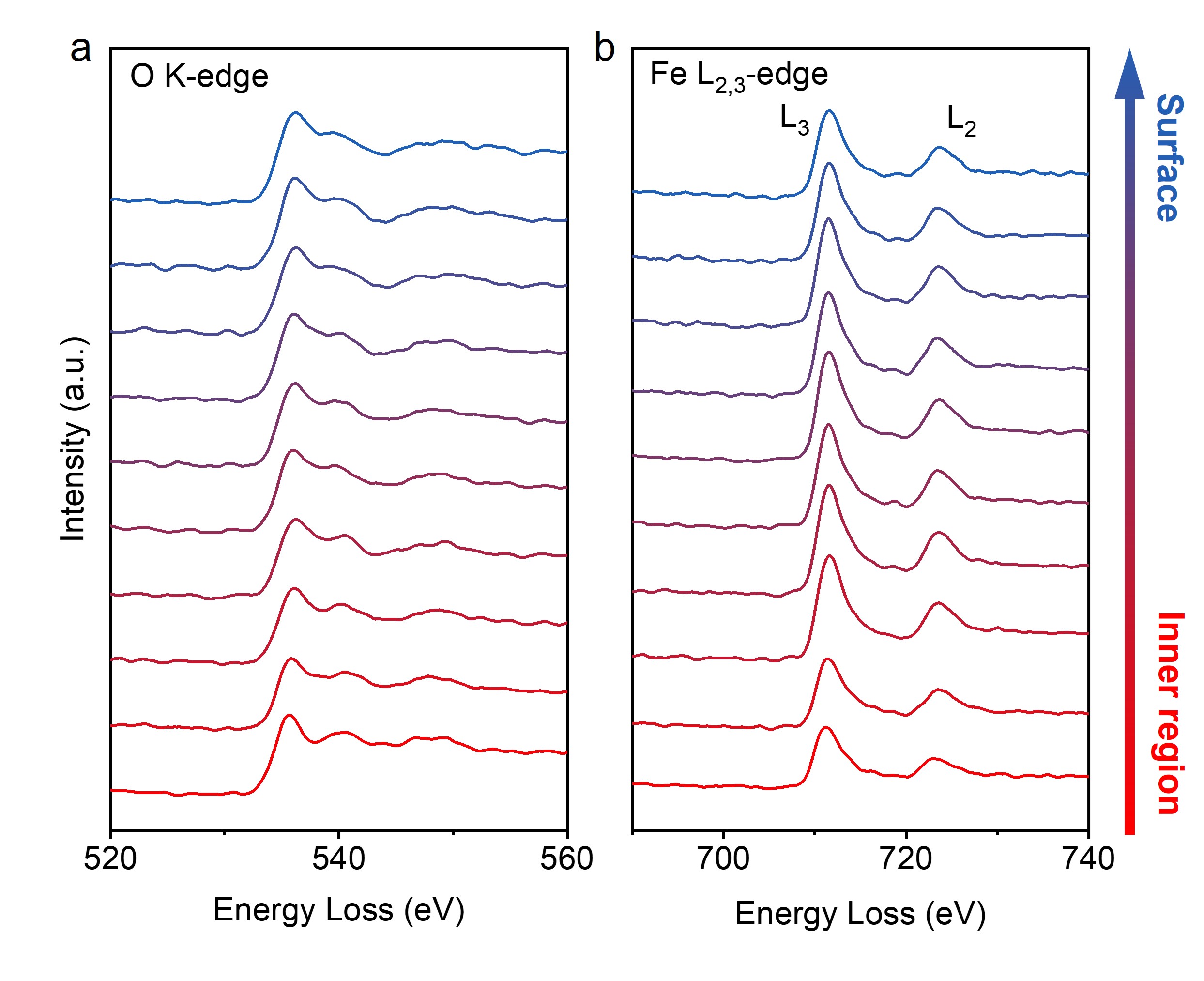


**Figure S26**. (a) O K-edge and (b) Fe L_2,3_-edge EEL spectra of FeNb_2_O_6_ after discharge to 0.1 V, measured in the direction from the inner region to surface of the particle, marked by the arrow in the AC-STEM image of **Figure S27**.


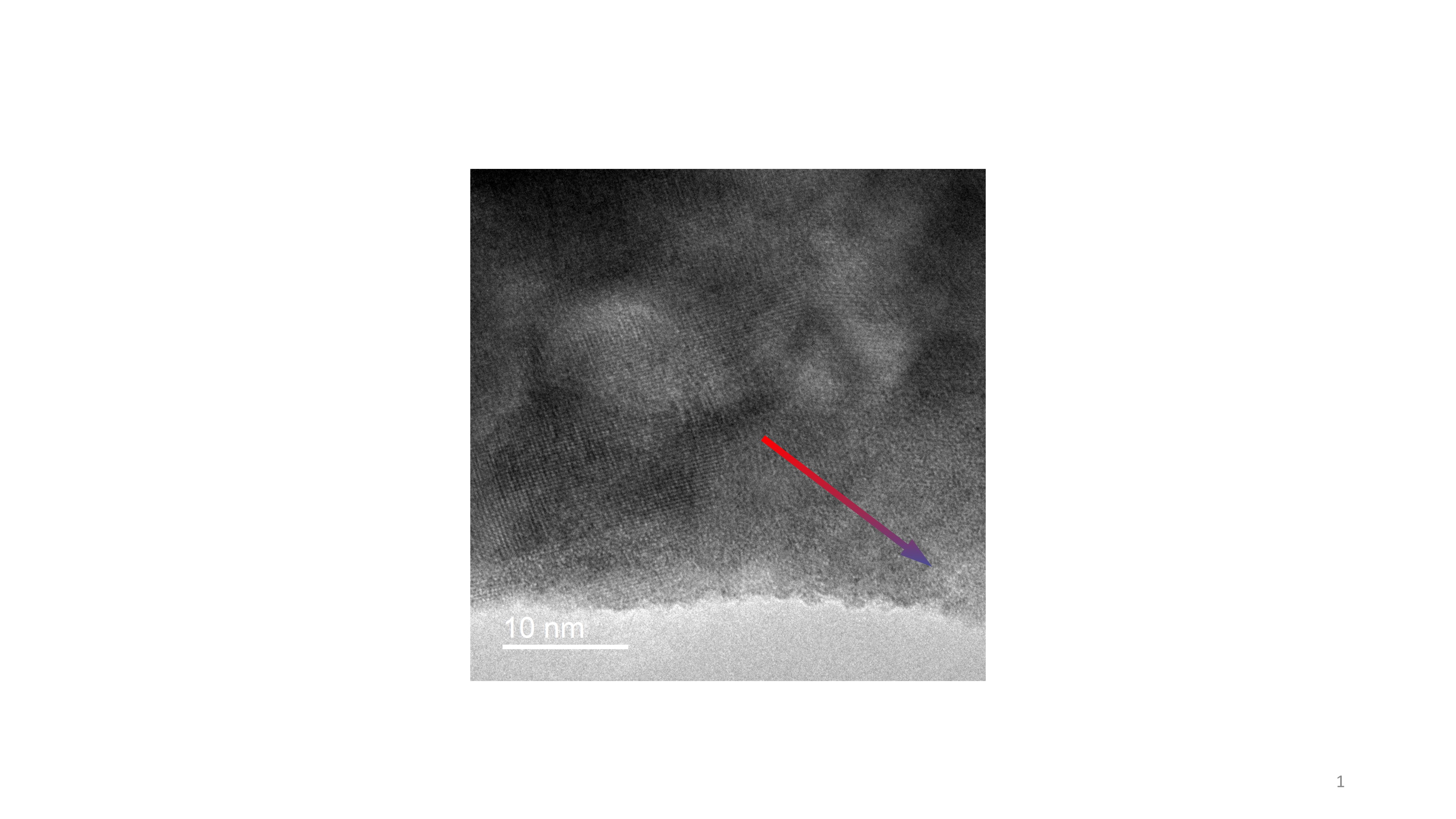


**Figure S27**. AC-STEM image of FeNb_2_O_6_ after discharge to 0.1 V.


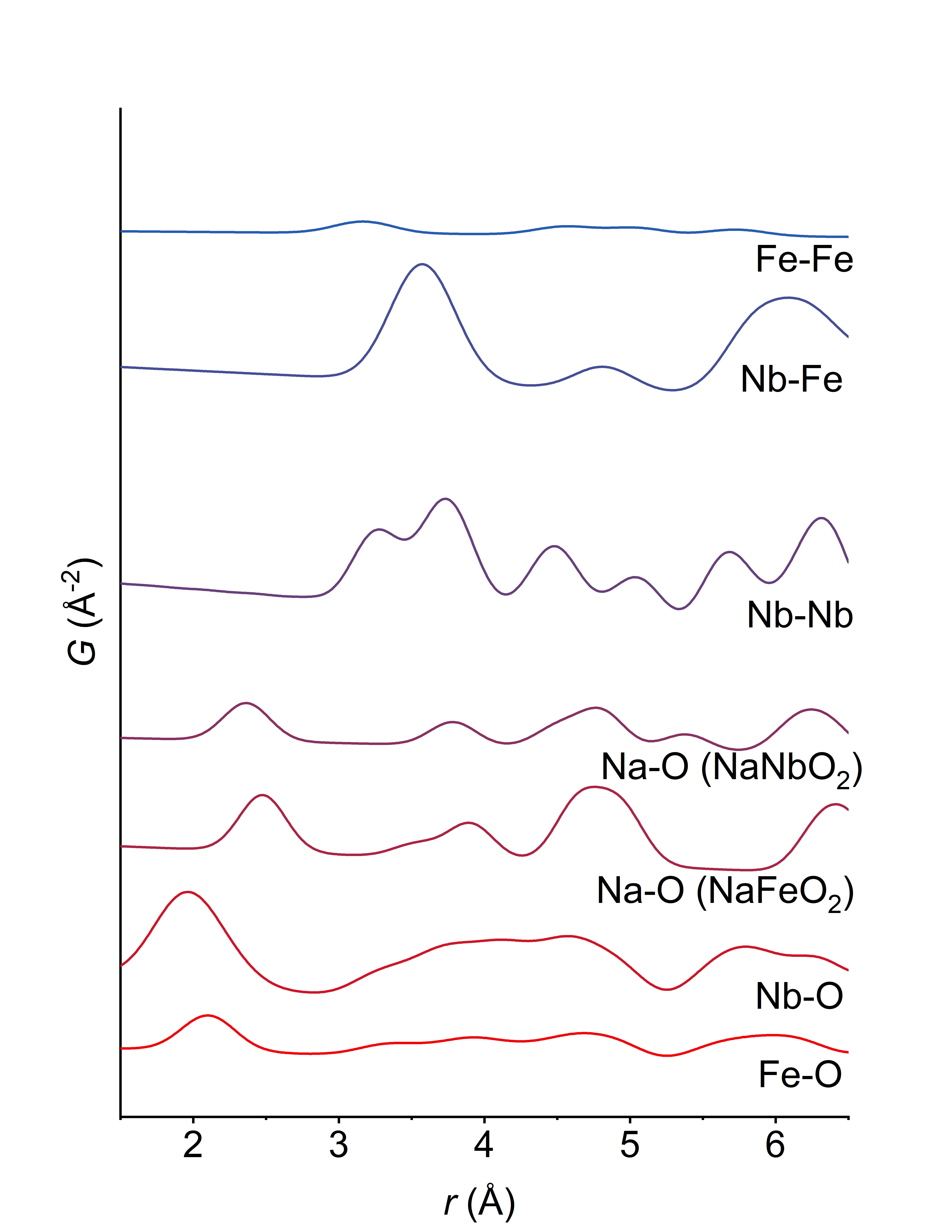


**Figure S28.** Calculated partial PDFs of Fe‒O, Nb‒O, Nb‒Nb, Nb‒Fe and Fe‒Fe atomic pairs in FeNb_2_O_6_.


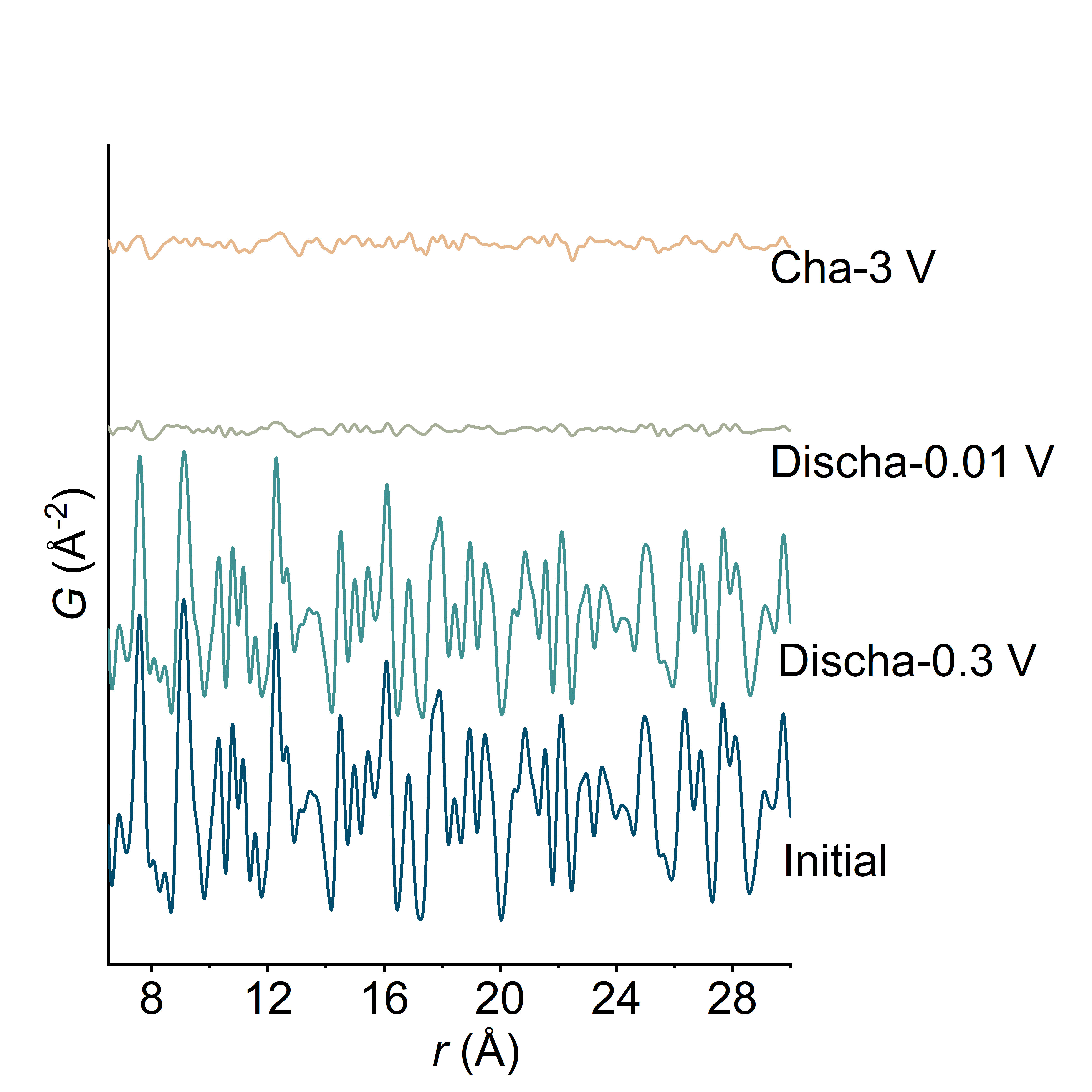


**Figure S29.** Long-range regions (6.5 < *r* < 30) of the ex situ synchrotron X-ray PDF of FeNb_2_O_6_@C at different voltage stages.

**
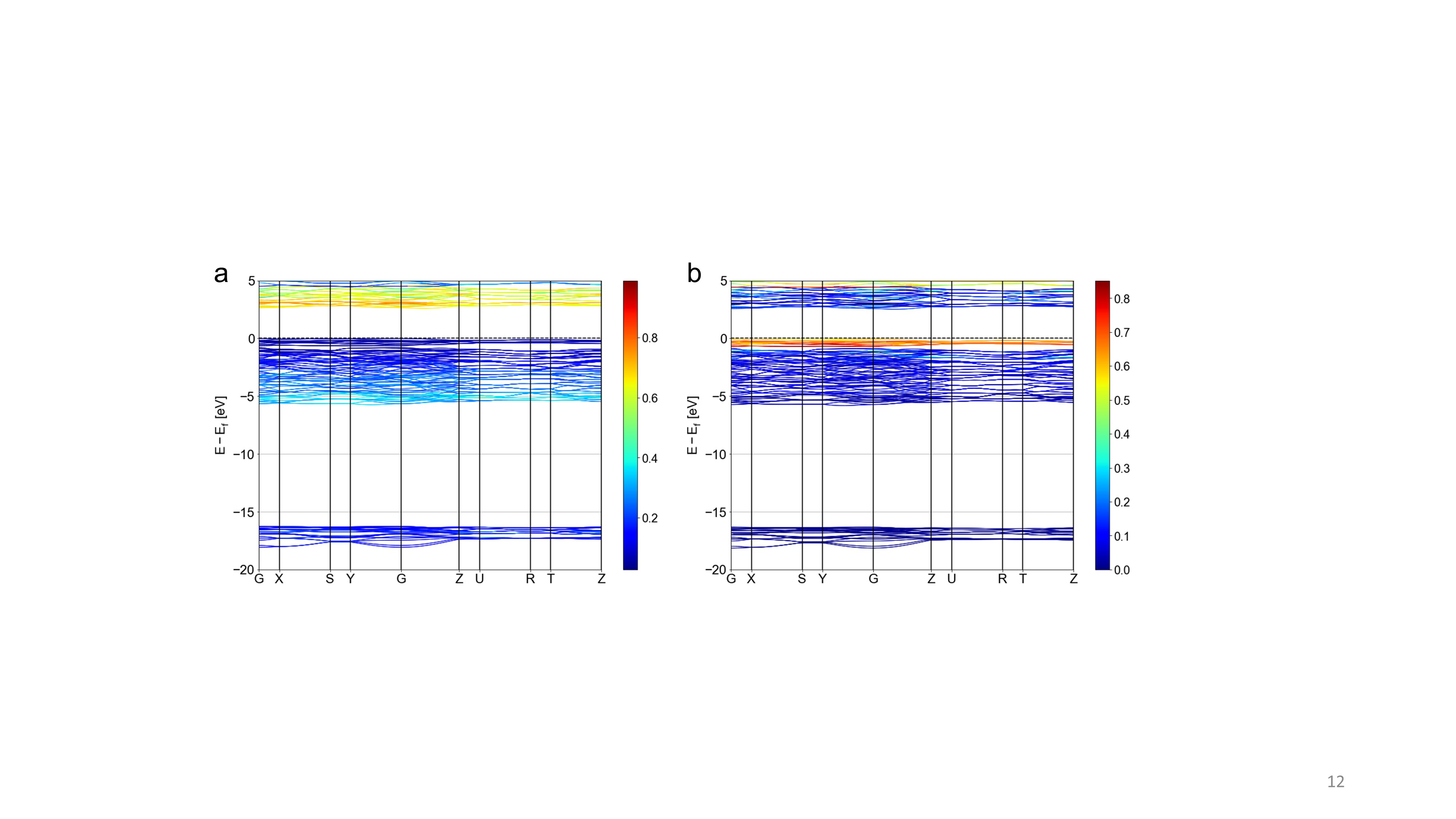
**

**Figure S30**. Band structure of FeNb_2_O_6_ calculated in GGA+U approximations. Eigenvalues are reported for k-values spanning G-X-S-Y-G-Z-U-R-T-Z direction in the Brillouin zone. The zero of the energy is the calculated top of the valence band. A color code is used to highlight contribution from Fe- and Nb-states.


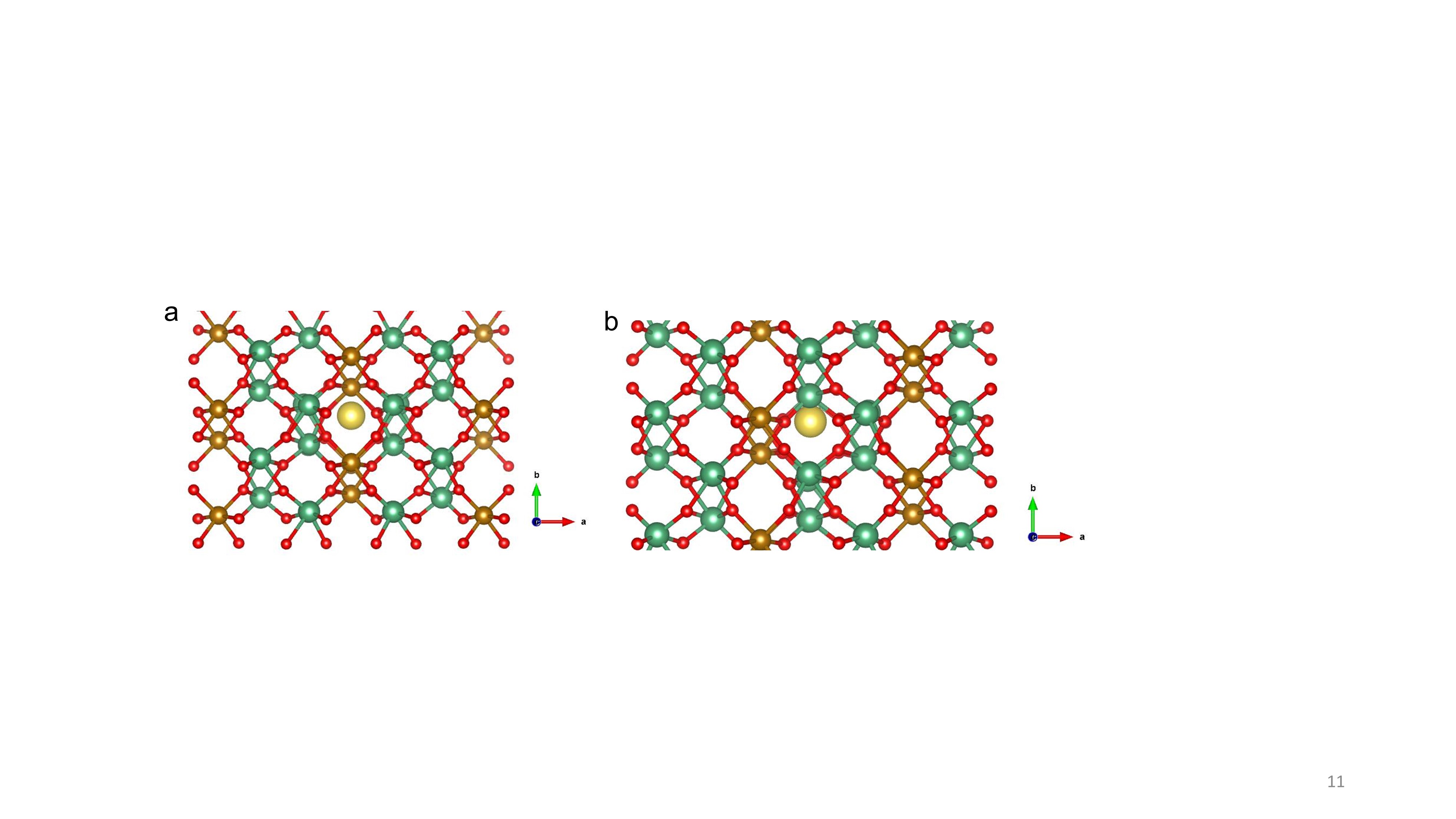


**Figure S31.** DFT relaxed configurations for the minimum energy structures of Na^+^ interstitial in a Fe (a) and Nb (b) *bc* plane. Fe, Nb, O and Na atoms are depicted as orange, green, red and yellow spheres, respectively.


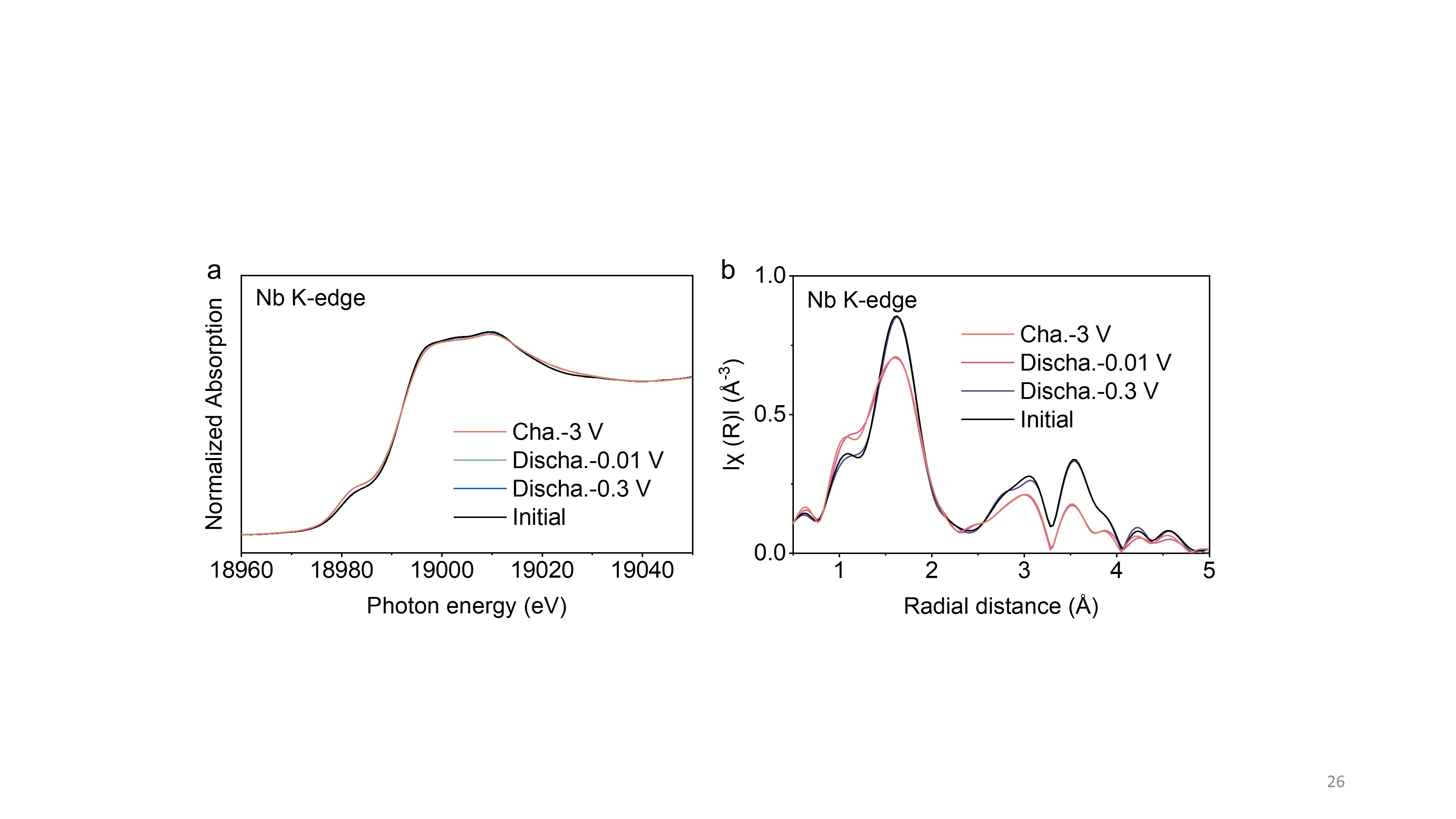


**Figure S32.** Ex situ Nb K-edge (a) XANES and (b) EXAFS spectra of Nb_2_O_5_@C at different voltage states.

**Table S1.** Rietveld refinement of the XRD pattern of FeNb_2_O_6_@C.

| Atom | x | y | z | *g* |
| --- | --- | --- | --- | --- |
| Fe | 0 | 0.331 | 0.250 | 1 |
| Nb | 0.339 | 0.319 | 0.251 | 1 |
| O | 0.096 | 0.104 | 0.073 | 1 |
| O | 0.419 | 0.116 | 0.099 | 1 |
| O | 0.756 | 0.124 | 0.079 | 1 |

Space group: *Pbcn*, *a* = 14.2459(5) Å, *b* = 5.73571(18) Å, *c* = 5.04636(14) Å, *α* = *β* = *γ* = 90°,

Vol = 412.340(16) Å^3^, (Z = 4), *R_wp_* = 3.47%.

**Table S2.** Rietveld refinement of the XRD pattern of FeNb_2_O_6_.

| Atom | x | y | z | *g* |
| --- | --- | --- | --- | --- |
| Fe | 0 | 0.331 | 0.250 | 1 |
| Nb | 0.339 | 0.319 | 0.251 | 1 |
| O | 0.096 | 0.104 | 0.073 | 1 |
| O | 0.419 | 0.116 | 0.099 | 1 |
| O | 0.756 | 0.124 | 0.079 | 1 |

Space group: *Pbcn*, *a* = 14.159(5) Å, *b* = 5.7020(21) Å, *c* = 5.0156(19) Å, *α* = *β* = *γ* = 90°,

Vol = 405.9(4) Å^3^, (Z = 4), *R_wp_* = 6.31%.

**Table 3**. Refinement results of the PDF of FeNb_2_O_6_ over the range 1.6 < r < 13.0 Å.

| Atom | *x* | *y* | *z* | Uiso |
| --- | --- | --- | --- | --- |
| Fe | 0 | 0.331 | 0.25 | 0.02 |
| Fe | 0.5 | 0.831 | 0.25 | 0.02 |
| Fe | 0 | 0.669 | 0.75 | 0.02 |
| Fe | 0.5 | 0.169 | 0.75 | 0.02 |
| Nb | 0.339 | 0.319 | 0.251 | 0.0213 |
| Nb | 0.161 | 0.819 | 0.251 | 0.0213 |
| Nb | 0.339 | 0.681 | 0.751 | 0.0213 |
| Nb | 0.161 | 0.181 | 0.751 | 0.0213 |
| Nb | 0.661 | 0.681 | 0.749 | 0.0213 |
| Nb | 0.839 | 0.181 | 0.749 | 0.0213 |
| Nb | 0.661 | 0.319 | 0.249 | 0.0213 |
| Nb | 0.839 | 0.819 | 0.249 | 0.0213 |
| O | 0.096 | 0.104 | 0.073 | 0.0127 |
| O | 0.404 | 0.604 | 0.073 | 0.0127 |
| O | 0.096 | 0.896 | 0.573 | 0.0127 |
| O | 0.404 | 0.396 | 0.573 | 0.0127 |
| O | 0.904 | 0.896 | 0.927 | 0.0127 |
| O | 0.596 | 0.396 | 0.927 | 0.0127 |
| O | 0.904 | 0.104 | 0.427 | 0.0127 |
| O | 0.596 | 0.604 | 0.427 | 0.0127 |
| O | 0.419 | 0.116 | 0.099 | 0.0127 |
| O | 0.081 | 0.616 | 0.099 | 0.0127 |
| O | 0.419 | 0.884 | 0.599 | 0.0127 |
| O | 0.081 | 0.384 | 0.599 | 0.0127 |
| O | 0.581 | 0.884 | 0.901 | 0.0127 |
| O | 0.919 | 0.384 | 0.901 | 0.0127 |
| O | 0.581 | 0.116 | 0.401 | 0.0127 |
| O | 0.919 | 0.616 | 0.401 | 0.0127 |
| O | 0.756 | 0.124 | 0.079 | 0.0127 |
| O | 0.744 | 0.624 | 0.079 | 0.0127 |
| O | 0.756 | 0.876 | 0.579 | 0.0127 |
| O | 0.744 | 0.376 | 0.579 | 0.0127 |
| O | 0.244 | 0.876 | 0.921 | 0.0127 |
| O | 0.256 | 0.376 | 0.921 | 0.0127 |
| O | 0.244 | 0.124 | 0.421 | 0.0127 |
| O | 0.256 | 0.624 | 0.421 | 0.0127 |

Space group: *Pbcn*, *a* = 14.2459 Å, *b* = 5.7357 Å, *c* = 5.0464 Å, *α* = *β* = *γ* = 90°,

Vol = 412.3424 Å^3^. *x*, *y*, and *z*: atomic coordinate, *U*_iso_: isotropic atomic displacement parameter. *R_wp_* = 10.1%.

**Table S4.** Curvefit parameters of the Fe K-edge EXAFS of FeNb_2_O_6_@C.

| Path | *d* / Å | *N* |  | *R* / Å | σ^2^ / Å |
| --- | --- | --- | --- | --- | --- |
| Fe-O1 | 2.09 | 2 |  | 2.08(1) | 0.007(0) |
| Fe-O2 | 2.14 | 4 |  | 2.12(1) | 0.007(0) |

S_0_^2^ of the first shell for this fit is 0.7. Δ*E*_0_ was refined as a global fit parameter. Data ranges: 2 ≤ *k* ≤ 12 Å^-1^, 1.1 ≤ *R* ≤ 2.5 Å. The number of variable parameters is 3, out of a total of 8.02 independent data points. R factor for the fitting is 0.7 %. The Debye-Waller factors were constrained as follows to reduce the number variables: σ^2^ (Fe-O1) = σ^2^ (Fe-O2).

**Table S5.** Curvefit parameters of the Nb K-edge EXAFS of FeNb_2_O_6_@C.

| Path | *d* / Å | *N* |  | *R* / Å | σ^2^ / Å |
| --- | --- | --- | --- | --- | --- |
| Nb-O1 | 1.92 | 1.5 |  | 1.94(1) | 0.017(2) |
| Nb-O2 | 1.96 | 2.0 |  | 1.98(1) | 0.017(2) |
| Nb-O3 | 2.08 | 1.5 |  | 2.10(1) | 0.017(2) |
| Nb-O4 | 2.28 | 1 |  | 2.30(1) | 0.017(2) |
| Nb-Nb1 | 3.27 | 1.3 |  | 3.32(1) | 0.002(1) |
| Nb-Nb2 | 3.67 | 2 |  | 3.72(1) | 0.002(1) |
| Nb-Fe1 | 3.52 | 1.3 |  | 3.50(1) | 0.001(1) |
| Nb-Fe2 | 3.62 | 1.7 |  | 3.60(1) | 0.001(1) |
| Nb-Fe3 | 3.73 | 2.9 |  | 4.70(1) | 0.001(1) |

S_0_^2^ of the first shell for this fit is 1.0. Δ*E*_0_ was refined as a global fit parameter. Data ranges: 2 ≤ *k* ≤ 12 Å^-1^, 1.1 ≤ *R* ≤ 3.7 Å. The number of variable parameters is 7, out of a total of 16.5 independent data points. R factor for the fitting is 1.9 %. The Debye-Waller factors were constrained as follows to reduce the number variables: σ^2^ (Nb-O1) = σ^2^ (Nb-O2) = σ^2^ (Nb-O3) = σ^2^ (Nb-O4) = σ^2^ (Nb-O5), σ^2^(Nb-Nb1) = σ^2^(Nb-Nb2) and σ^2^(Nb-Fe1) = σ^2^(Nb-Fe2) = σ^2^(Nb-Fe3).

**Table S6.** Comparison of the electrochemical performance of *T*-Nb_2_O_5_@C with that of other previously reported Nb_2_O_5_-based electrodes.

| Material | Voltage range (V vs. Li^+^/Li) | Initial reversible capacity (mAh g^-1^) | Rate performance | Capacity after long cycling |
| --- | --- | --- | --- | --- |
| Mesoporous Nb_2_O_5_/C composite ^25^ | 0.01-2.5 | 175 (at 50 mA g^-1^) | ~80 mAh g^-1^ at 0.5 A g^-1^ | ~100 mAh g^-1^ after 300 cycles |
|  |  |  | 60 mAh g^-1^ at 1 A g^-1^ |  |
| Pomegranate-like Nb_2_O_5_ /C@NC nanocrystal ^26^ | 0.01-3.0 | 201 (at 100 mA g^-1^) | 109 mAh g^-1^ at 1 A g^-1^ | 110 mAh g^-1^ after 500 cycles |
|  |  |  | 82 mAh g^-1^ at 2 A g^-1^ |  |
| Nb_2_O_5_ carbon nanofiber ^27^ | 0.01-3.0 | 181 (at 100 mA g^-1^) | ~100 mAh g^-1^ at 1 A g^-1^ | ~150 mAh g^-1^ after 100 cycles |
|  |  |  | ~90 mAh g^-1^ at 2 A g^-1^ |  |
| Nano-solid spherical Nb_2_O_5_/C composite ^28^ | 0.01-3.0 | 210 (at 100 mA g^-1^) | 112 mAh g^-1^ at 1 A g^-1^ | 144 mAh g^-1^ after 2000 cycles |
|  |  |  | 78 mAh g^-1^ at 2 A g^-1^ |  |
| *T*-Nb_2_O_5_@C (This work) | 0.01-3.0 | 180.5 (at 20 mA g^-1^) | 93.5 mAh g^-1^ at 1 A g^-1^ | ~90 mAh g^-1^ after 2600 cycles |
|  |  |  | 82 mAh g^-1^ at 2 A g^-1^ |  |

**References:**

1. M. Filowitz, R. K. C. Ho, W. G. Klemperer, W. Shum, *Inorg. Chem*. **1979**, *18*, 93.
2. B. H Toby, R. B. Von Dreele, *J. Appl. Cryst*. **2013**, *46*, 544.
3. J. Kieffer, V. Valls, N. Blancb, C. Hennig, *J. Synchrotron Rad*. **2020**, *27*, 558.
4. P. Juhás, T. Davis, C. L. Farrow, S. J. L Billinge, *J. Appl. Cryst*. **2013**, *46*, 560.
5. X. Yang, P. Juhas, C. L. Farrow, S. J. L. Billinge, arXiv, **2014**, *1402*, 3163.
6. A. Guilherme Buzanich, M. Radtke, K. V. Yusenko, T. M. Stawski, A. Kulow, C. T. Cakir, B. Röder, C. Naese, R. Britzke, M. Sintschuk, F. Emmerling, *J. Chem. Phys.* **2023**, *158*, 244202.
7. M. Newville, *J. Phys. Conf. Ser.* **2013**, *430*, 012007.
8. G. Kresse, J. Hafner, *J. Phys.: Condens. Matt.* **1994**, *6*, 8245.
9. G. Kresse, D. Joubert, *Phys. Rev*. **1999**, *59*, 1758.
10. G. Kresse, J. Hafner, *Phys. Rev. B* **1993**, *47*, 558.
11. G. Kresse, J. Furthmüller, *Comput. Mat. Sci*. **1996**, *6*, 15.
12. G. Kresse, J. Furthmüller, *Phys. Rev. B* **1996**, *54*, 11169.
13. J. P. Perdew, K. Burke, M. Ernzerhof, *Phys. Rev. Lett.* **1996**, *77*, 3865.
14. S. L. Dudarev, G. A. Botton, S. Y. Savrasov, C. J. Humphreys, A. P. Sutton, *Phys. Rev. B* **1998**, *57*, 1505.
15. M. E. Arroyo y de Dompablo, Yueh-Lin Lee, D. Morgan, *Chemistry of Materials* **2010** *22*, 906.
16. G. Mills, H. Jonsson, G. K. Schenter, *Surf. Sci*. **1995**, *324*, 305.
17. P. Senguttuvan, G. Rousse, M. E. Arroyo y de Dompablo, H. Vezin, J. M. Tarascon, M. R. Palacin, *J. Am. Chem. Soc.* **2013**, *135*, 3897.
18. C. Xu, Y. Xu, C. Tang, Q. Wei, J. Meng, L. Huang, L. Zhou, G. Zhang, L. He, L. Mai, *Nano Energy* **2016**, *28*, 224.
19. Y. Sun, L. Zhao, H. Pan, X. Lu, L. Gu, Y.-S. Hu, H. Li, M. Armand, Y. Ikuhara, L. Chen, X. Huang, *Nat. Commun*. **2013**, *4*, 1870.
20. D. Wu, X. Li, B. Xu, N. Twu, L. Liu, G. Ceder, *Energy Environ. Sci.* **2015**, *8*, 195.
21. K.-T. Kim, G. Ali, K. Y. Chung, C. S. Yoon, H. Yashiro, Y.-K. Sun, J. Lu, K. Amine, S. T. Myung, *Nano Lett*. **2014**, *14*, 416.
22. Y. Wang, X. Yu, S. Xu, J. Bai, R. Xiao, Y.-S. Hu, H. Li, X. Q. Yang, L. Chen, X. Huang, *Nat. Commun*. **2013**, *4*, 2365.
23. H. Pan, X. Lu, X. Yu, Y.-S. Hu, H. Li, X.-Q. Yang, L. Chen, *Adv. Energy Mater*. **2013**, *3*, 1186.
24. Y. Huang, X. Li, J. Luo, K. Wang, Q. Zhang, Y. Qiu, S. Sun, S. Liu, J. Han, Y. Huang, *ACS Appl. Mater. Interfaces* **2017**, *9*, 8696.
25. H. Kim, E. Lim, C. Jo, G. Yoon, J. Hwang, S. Jeong, J. Lee, K. Kang, *Nano Energy*, **2015**, *16*, 62.
26. [J. Yuan](https://scholar.google.com.hk/citations?user=MqVInk8AAAAJ&hl=zh-CN&oi=sra), X. Li, J. Liu, S. Zuo, X. Li, F. Li, Y. Gan, H. He, [X. Xu](https://scholar.google.com.hk/citations?user=RnszcloAAAAJ&hl=zh-CN&oi=sra), X. Zhang, J. Meng, *J. Colloid Interface Sci.*, **2022**, *613*, 84.
27. X. Zhu, L. Lu, Q. Deng, G. Mai, Z. Mei, H. Ji, L. Yao, *ACS Appl. Nano Mater.* **2025**, *8*, 200.
28. H. He, Y. Gan, M. Mu, J. Yuan, C. Zhang, X. Zhang, X. Li, X. Ma, H. Yu, J. Mou, J. Liu, *J. Solid State Electrochem*. **2023**, *27*, 2337.
